# Targeting PRMTs with small-molecule inhibitors: a comprehensive review

**DOI:** 10.1039/d6md00493h

**Published:** 2026-07-27

**Authors:** Monica Viviano, Alessandra Cipriano, Benito D'Ascoli, Ciro Milite, Sabrina Castellano, Gianluca Sbardella

**Affiliations:** a Department of Pharmacy, University of Salerno Via Giovanni Paolo II 132 84084 Fisciano, SA Italy gsbardella@unisa.it; b PhD Program in Drug Discovery and Development, University of Salerno Italy

## Abstract

Protein arginine methyltransferases (PRMTs) constitute a family of *S*-adenosyl-l-methionine (SAM)-dependent enzymes responsible for the methylation of arginine residues on histone and non-histone proteins. Through this post-translational modification, PRMTs regulate diverse cellular processes, including chromatin dynamics, transcriptional control, RNA metabolism, and the DNA damage response. Increasing evidence links aberrant PRMT activity to tumourigenesis and other pathological conditions, positioning these enzymes as attractive targets for pharmacological intervention. In recent years, extensive medicinal chemistry campaigns have led to the identification of a wide range of small-molecule PRMT modulators. Distinct inhibitor classes have been developed to exploit different binding regions within the catalytic machinery, including SAM-competitive ligands, substrate-pocket binders, and bisubstrate analogues capable of simultaneously engaging both sites. For some isoforms, particularly PRMT5, these strategies have yielded compounds with strong biochemical potency, robust cellular activity, and encouraging pharmacological profiles. In parallel, inhibitors targeting PRMT1, PRMT4 (CARM1), PRMT6, and PRMT7 have emerged as valuable chemical probes to investigate the biological roles of these enzymes. More recently, innovative approaches—such as methylthioadenosine (MTA)-cooperative inhibitors designed to exploit methylthioadenosine phosphorylase (*MTAP*)-deleted tumours and targeted protein degradation (TPD) strategies—have further expanded the therapeutic landscape. This review summarizes the current state of small-molecule PRMT modulation, highlighting representative chemotypes, binding modes, and structure–activity relationships (SARs) reported across individual PRMT isoforms. Particular attention is given to compounds that have advanced into preclinical or clinical evaluation, alongside emerging strategies aimed at improving selectivity and therapeutic applicability. Collectively, these advances illustrate the rapid evolution of PRMT-directed medicinal chemistry and its growing relevance to the development of targeted epigenetic therapies.

## Abstract 2

A comprehensive overview of PRMT small-molecule inhibitors and degraders, covering structural classification, allosteric and MTA-cooperative strategies, and novel targeted degradation modalities.

## Graphical abstract

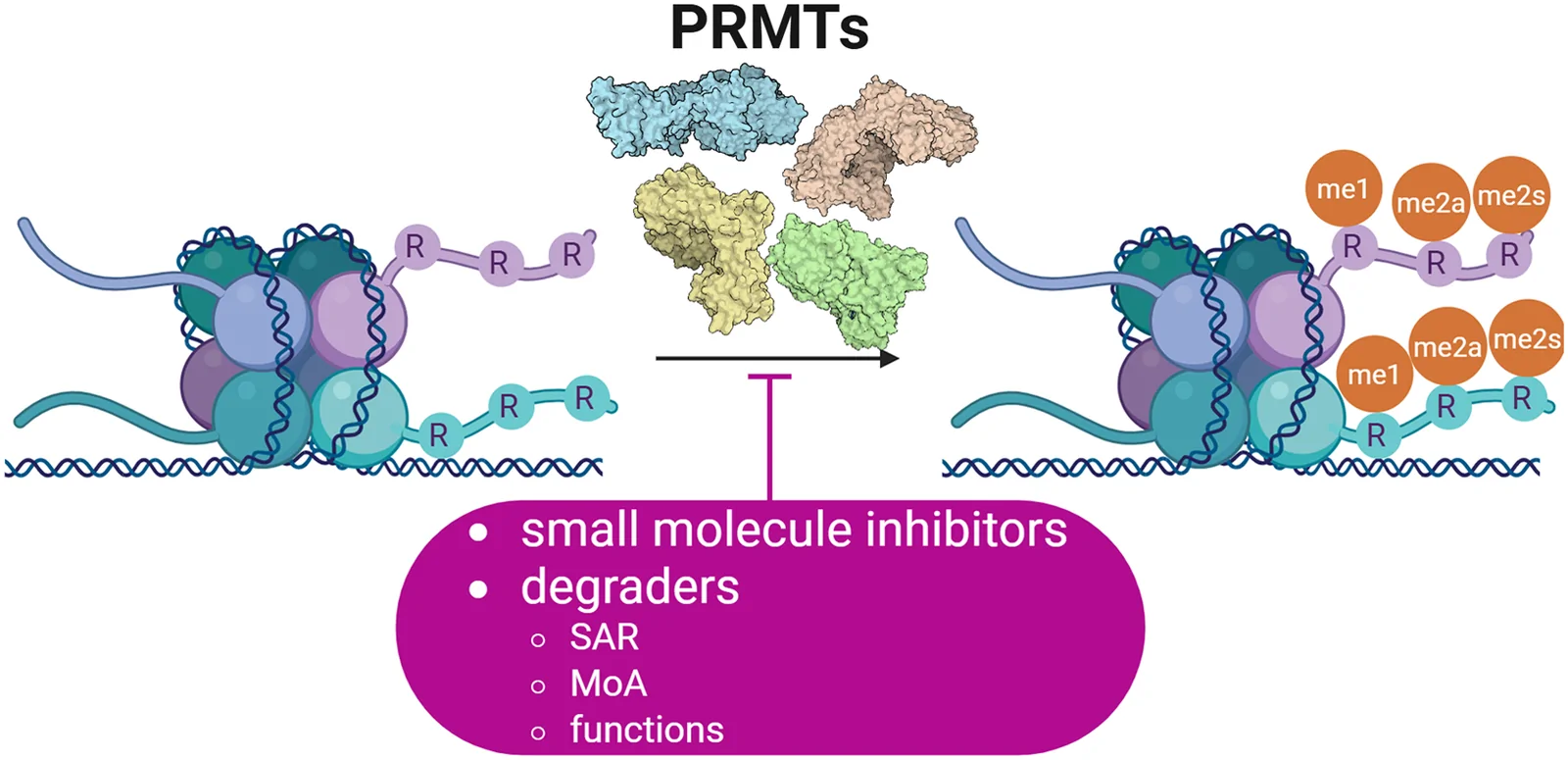

## Introduction

Protein arginine methylation is a widespread post-translational modification (PTM) and epigenetic regulatory mechanism with a cellular prevalence comparable to phosphorylation and ubiquitination.^1^ In mammalian cells, it plays fundamental roles in DNA damage repair, RNA processing, signal transduction and translational control.^2–4^ This modification is catalysed by the protein arginine methyltransferase (PRMT) family, which transfers a methyl group from *S*-adenosyl-l-methionine (SAM) to the guanidinium group of arginine residues in substrate proteins.^5^

**Fig. 1:**
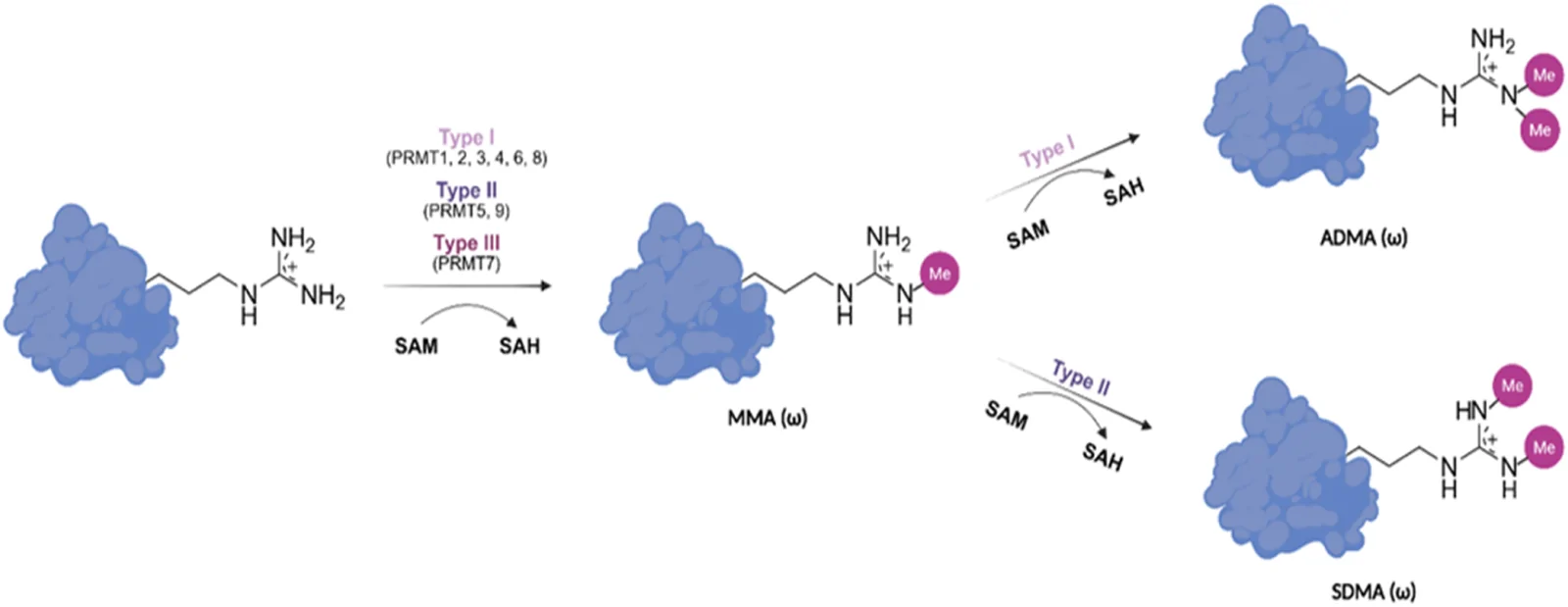
Schematic representation of arginine methylation catalysecatalysed by protein arginine methyltransferases (PRMTs). PRMT enzymes transfer methyl groups from *S*-adenosylmethionine (SAM) to the guanidino nitrogen atoms of arginine residues, generating *S*-adenosylhomocysteine (SAH) as a by-product. Type I, II and III PRMTs first catalysecatalyse the formation of monomethylarginine (MMA). Type I PRMTs subsequently generate asymmetric dimethylarginine (ADMA), whereas type II PRMTs produce symmetric dimethylarginine (SDMA). Type III PRMTs catalysecatalyse monomethylation. These distinct methylarginine marks regulate protein–protein interactions, transcriptional programs and signalling pathways, and provide the biochemical basis for developing PRMT subtype-selective inhibitors.

To date, nine mammalian PRMT isoforms (PRMT1–9) have been identified.^1^ Based on the chemical nature of their methylation products, PRMTs are classified into three functional types. Type I enzymes (PRMT1, 2, 3, 4, 6 and 8) catalyse the formation of monomethylarginine (MMA) and asymmetric dimethylarginine (ADMA); type II enzymes (PRMT5 and PRMT9) generate MMA and symmetric dimethylarginine (SDMA); PRMT7, the sole type III enzyme, produces exclusively MMA (Fig. 1).^6,7^ Among these marks, ADMA is the most abundant in mammalian cells, whereas SDMA and MMA account for approximately 10% and 1% of total arginine methylation, respectively, underscoring the dominant contribution of type I PRMTs to the regulation of cellular processes.^8^

Structurally, PRMTs share a highly conserved catalytic core composed of two principal domains: an N-terminal Rossmann fold (the SAM-binding domain) and a C-terminal β-barrel domain responsible for substrate recognition. Extending from the β-barrel is an α-helical dimerization arm that interacts with the SAM-binding domain of the adjacent monomer, an interaction essential for assembling the catalytically competent PRMT dimer.^6^ This conserved methyltransferase architecture provides the structural framework for methyl transfer chemistry across the entire family. Despite this structural conservation, individual PRMTs display notable variations in domain organization. Several members contain additional N-terminal modules (*e.g.*, PRMT2, PRMT3 and PRMT4), whereas PRMT7 and PRMT9 possess a duplicated catalytic domain that enhances substrate recognition and contributes to isoform-specific regulatory features.^9^ Beyond catalytic diversity, PRMT-dependent methyl marks serve as molecular docking platforms for effector proteins.^10^ Tudor-domain-containing proteins represent the principal class of “readers” that recognize methylated arginine motifs, although additional non-Tudor interactors have been described. Through these writer–reader networks, PRMTs modulate transcriptional regulation, RNA metabolism, DNA repair and signalling cascades.^11^ Given their pervasive involvement in cellular homeostasis and tumour biology, PRMT enzymes have emerged as attractive targets for small-molecule intervention. This review summarizes the structural features and biological roles of individual PRMT family members, highlighting recent progress in medicinal chemistry aimed at discovering and refining potent and selective modulators.

## Type I PRMTs

### PRMT1

PRMT1 is the predominant type I protein arginine methyltransferase and the principal cellular source of ADMA, accounting for ∼85% of total arginine methylation activity in mammalian tissues.^12^ Identified through homology to the yeast enzyme Hmt1/Rmt1, PRMT1 methylates glycine- and arginine-rich (GAR) motifs, histones and RNA-binding proteins such as hnRNPA1 (heterogeneous nuclear RiboNucleoProtein A1).^13^ Genetic studies have established its essential role *in vivo*, as germline *Prmt1* deletion causes early embryonic lethality, whereas conditional knockout models facilitate tissue-specific functional analysis.^14,15^

PRMT1 regulates transcriptional and post-transcriptional programs by methylating diverse substrates involved in chromatin regulation, RNA metabolism and oncogenic signalling.^16,17^ PRMT1-dependent methylation stabilizes EZH2 in breast cancer, enhances TWIST1-driven epithelial–mesenchymal transition (EMT) in lung cancer, promotes GLI1 oncogenic activity in pancreatic ductal adenocarcinoma, and supports androgen receptor-dependent transcription in prostate cancer.^18–21^ PRMT1 also contributes to genome stability by regulating DNA damage response factors, including BRCA1, MRE11 and 53BP1.^22^ Notably, tumours harbouring mutations in RNA splicing factors exhibit heightened dependence on type I PRMT activity, suggesting potential patient-stratification biomarkers for PRMT1-targeted therapies.^23^ Beyond oncology, PRMT1 dysregulation is implicated in metabolic, cardiovascular and inflammatory disorders.^24,25^ Collectively, these findings establish PRMT1 as a central regulator of cellular homeostasis and a premier target for medicinal chemistry campaigns.

### Pan-type I PRMT inhibitors with strong PRMT1 engagement

**Chart 1:**
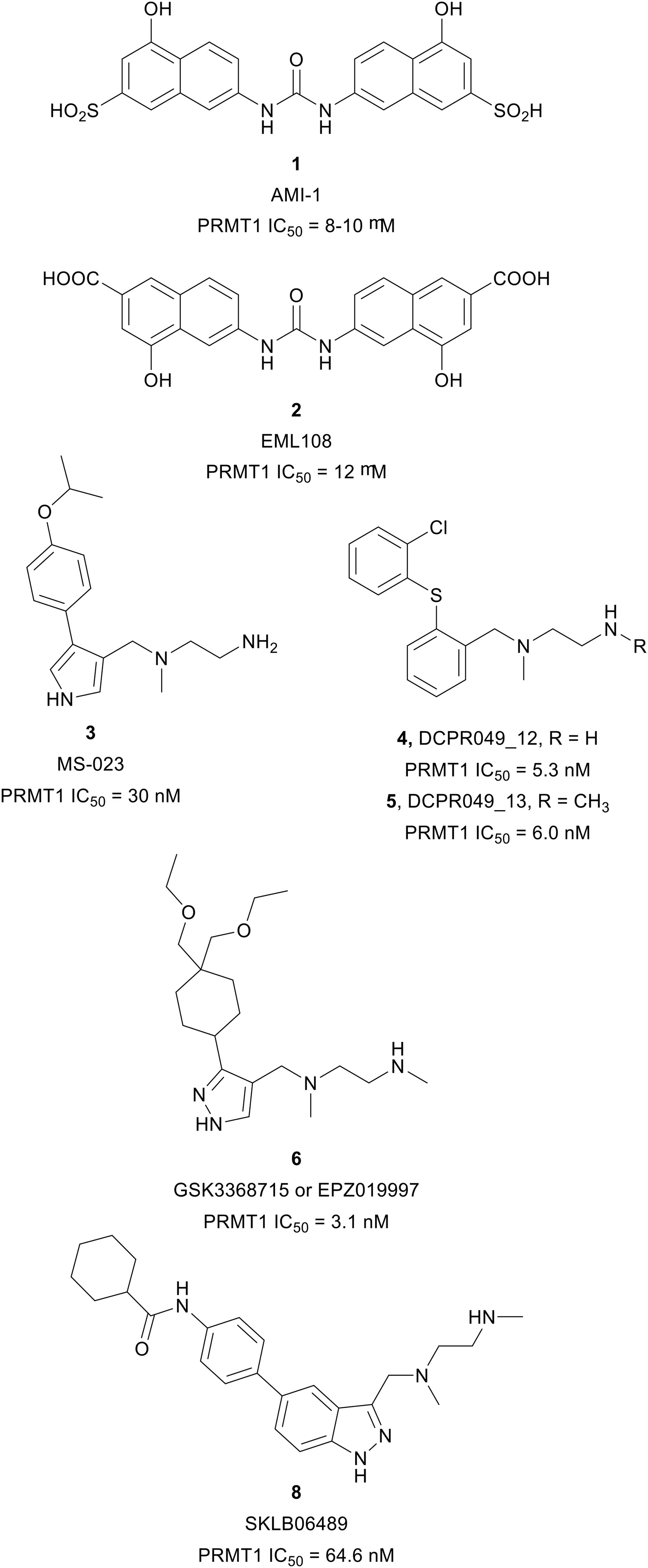
Representative structures of pan-type I PRMT inhibitors. All IC_50_ values were determined in biochemical assays unless otherwise stated. Early compounds such as 1 (AMI-1) and 2 (EML108) feature highly polar, symmetric scaffolds with limited potency. Subsequent development led to more druglike inhibitors, including 3 (MS-023) and thiophene-based derivatives (4–5), with improved activity. Advanced compounds such as 6 (GSK3368715 or EPZ019997) and 8 (SKLB0489) exhibit enhanced potency and cellular efficacy, illustrating the progression toward optimized pan-type I PRMT inhibitors.

The earliest small molecules identified as PRMT1 inhibitors are characterized as pan-type I PRMT inhibitors due to their lack of selectivity against other type I enzymes, most notably PRMT6 and PRMT8 (Chart 1). The suramin-like sulfonated urea compound 1 (AMI-1) was identified through high-throughput biochemical screening and represents one of the first reported small-molecule, substrate-competitive inhibitors of PRMT enzymes. Although it inhibits PRMT1 with micromolar potency (IC_50_ ≈ 8–10 μM), 1 lacks isoform selectivity (IC_50_ = 101 μM and 74 μM against PRMT3 and PRMT4, respectively), limiting its utility to that of an early chemical probe.^26^ This compound paved the way for the development of the isosteric bis-4-hydroxy-2-naphthoic acid 2, which inhibits the arginine methylation of cellular proteins in whole-cell assays with activities comparable to—or exceeding—those of 1. Notably, 2 and its derivatives demonstrate selective inhibition of arginine methyltransferases (IC_50_ = 12 μM, 90 μM and 53 μM against PRMT1, PRMT3 and PRMT4, respectively) while remaining essentially inactive against the lysine methyltransferase SET7/9.^27^

The ethylenediamine scaffold has been extensively exploited to develop potent pan-type I PRMT inhibitors. These compounds were rationally designed to engage Asp84, an amino acid essential to the enzyme's catalytic machinery, as well as the substrate arginine-binding channel. The first-in-class compound 3 (MS-023) features high potency against the entire type I family (IC_50_ = 30 nM, 119 nM, 83 nM, 4 nM and 5 nM against PRMT1, PRMT3, PRMT4, PRMT6 and PRMT8, respectively). It acts as a reversible, substrate-competitive inhibitor and has been widely used to characterize type I PRMT blockade. This compound is cell-active and effectively suppresses cellular ADMA in cells, facilitating functional studies in splicing and DNA-damage contexts.^28^

Following the discovery of 3, several highly potent type I PRMT analogues were identified. In 2017, using structure-based virtual screening, Luo and co-workers identified 4 (DCPR049_12) and 5 (DCPR049_13), which exhibit low-nanomolar potency (IC_50_ = 5.3 nM and 6.0 nM, respectively) and high selectivity against type I PRMTs (for compound 4, IC_50_ = 22 nM, 63 nM, >100 μM, 1.2 nM and 1.1 nM against PRMT3, PRMT4, PRMT6 and PRMT8, respectively) over 10 other methyltransferases and acetyltransferases. In multiple leukemia cell lines, these compounds, able to occupy the substrate binding site of PRMT1, display robust cellular engagement, inducing cell-cycle arrest and apoptosis.^29^ The ethylendiamino derivative 6 (GSK3368715, also known as EPZ019997) potently inhibits PRMT1 (IC_50_ = 3.1 nM) and other type I PRMTs (IC_50_ = 48 nM, 1148 nM, 5.7 nM and 1.7 nM against PRMT3, PRMT4, PRMT6 and PRMT8, respectively). In 2018, compound 6 became the first type I PRMT inhibitor to enter clinical evaluation for cancer treatment.^30^ It advanced to phase I trials for solid tumours and diffuse large B-cell lymphoma (DLBCL; NCT03666988) but was eventually discontinued due to limited clinical efficacy.

First-generation pan-type PRMT inhibitors were primarily useful as proof-of-concept chemical probes but generally suffered from pharmacological properties. In contrast, second-generation pan-type I PRMT inhibitors include compounds optimized for oral bioavailability and significantly improved pharmacokinetics profiles. Compound 7 (CTS-2190, structure not disclosed) is a multi-target PRMT1/3/4/6 inhibitor with a PRMT1 IC_50_ of 1.5 μM. It is currently undergoing phase I/II evaluation (NCT06224387) for refractory solid tumours, demonstrating efficient ADMA suppression and favourable tolerability.^31^ More recently, the pyrazole-derived 8 (SKLB06489) extended this class, inhibiting PRMT1 with an IC_50_ of 64.6 nM as well as PRMT6 and PRMT8 (IC_50_ = 4.2 and 51.3 nM, respectively), while demonstrating robust oral efficacy in triple-negative breast cancer (TNBC) models. Collectively, pan-type I agents achieve nanomolar biochemical potency against PRMT1 and remain the most clinically advanced PRMT1-active chemotypes.

### Selective PRMT1 inhibitors

**Chart 2:**
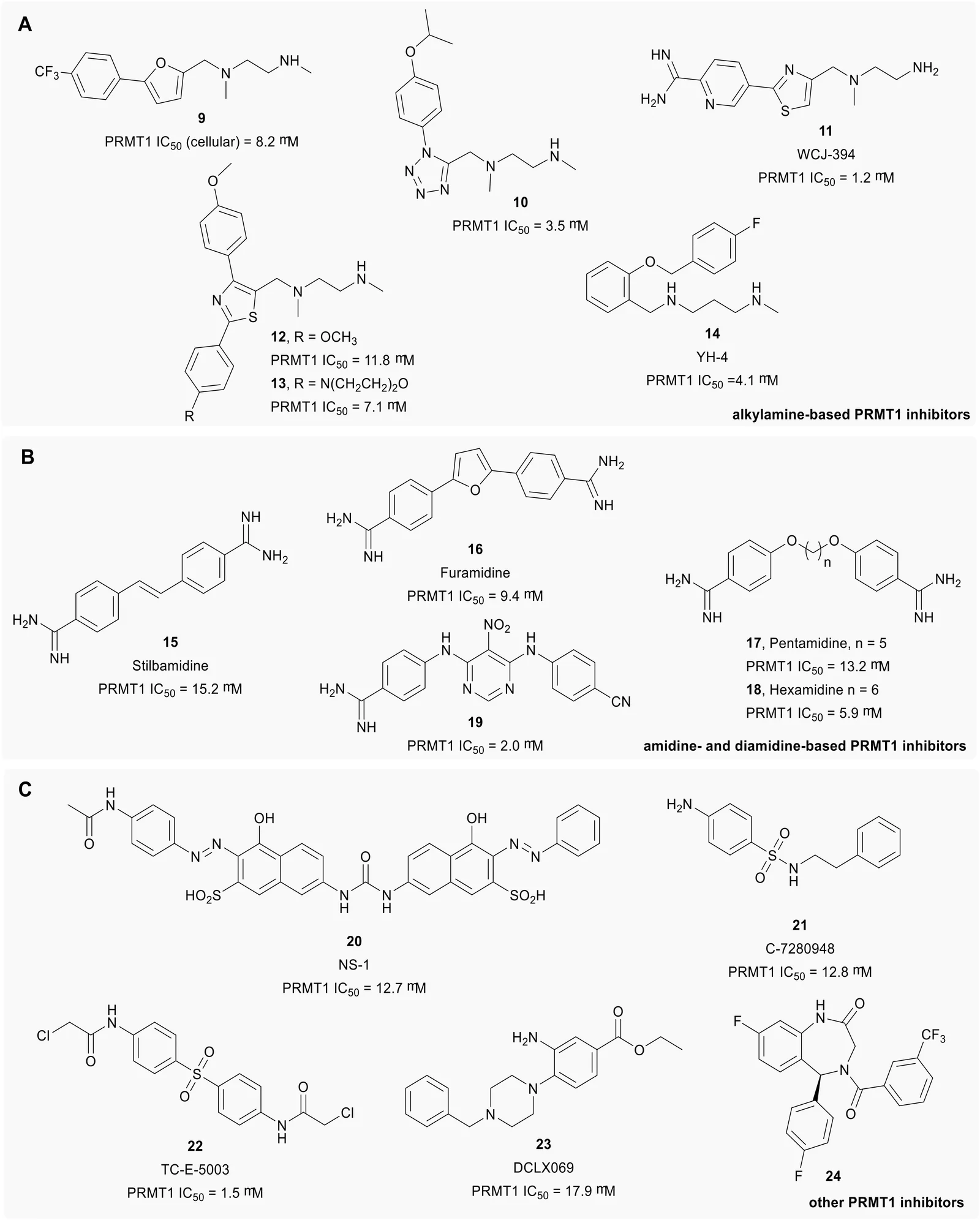
Representative structures of PRMT1 inhibitors, classified according to their core chemical features. All IC_50_ values were determined in biochemical assays unless otherwise stated. (A) Alkylamine-based inhibitors (compounds 9–14) bearing motifs able to mimic the arginine guanidinium group and enable substrate-competitive binding through electrostatic and hydrogen-bonding interactions within the peptide-binding channel. (B) Amidine- and diamidine-based inhibitors (compounds 15–19), in which the amidine functionalities act as guanidinium mimics, supporting substrate-site engagement and improved potency. (C) Structurally diverse PRMT1 inhibitors (compounds 20–24) identified through various discovery approaches, including high-throughput and virtual screening, encompassing both highly polar scaffolds with limited selectivity and more druglike molecules optimized for cellular activity.

In parallel with pan-type I inhibitors, a distinct class of selective PRMT1 inhibitors has emerged from structure-based design, virtual screening and scaffold refinement campaigns (Chart 2).^32–34^ These compounds predominantly act through substrate competitive binding mechanisms, exploiting subtle physicochemical and geometric features of the PRMT1 peptide binding channel to achieve isoform selectivity.

The development of selective PRMT1 inhibitors remains inherently challenging owing to the high structural conservation of the catalytic domains across type I PRMTs. In particular, both the SAM-binding site and the substrate arginine-binding channel display remarkable sequence and structural similarity, leaving only subtle differences in pocket topology and physicochemical properties that can be exploited to achieve isoform discrimination. Moreover, PRMT1 is the predominant type I PRMT in mammalian cells, accounting for most of the asymmetric arginine dimethylation, and shares numerous substrates and signalling pathways with other family members. Consequently, achieving biochemical selectivity does not necessarily translate into functional selectivity in cellular systems, where overlapping substrate specificity and compensatory methylation by related PRMTs may attenuate the biological effects of selective inhibition. These considerations explain why the discovery of truly isoform-selective PRMT1 inhibitors with robust cellular efficacy remains considerably more challenging than the optimization of biochemical potency alone.

Early efforts toward selective PRMT1 inhibition focused on the chemical optimization of pan-type I PRMT inhibitors featuring an ethylenediamine pharmacophore (Chart 2A). Homology model-guided design by Wang and co-workers led to furan-based compound 9 (IC_50_ = 8.2 μM), in which the protonated ethylenediamine moiety anchors into the substrate-binding pocket *via* electrostatic and hydrogen-bonding interactions with Asp84 and Glu152.^33^ Subsequent bioisosteric replacement of the furan heterocycle with a tetrazole yielded compound 10, which displays improved potency (IC_50_ = 3.5 μM) and at least 30-fold selectivity over PRMT5, while successfully reducing cellular ADMA and attenuating Wnt/β-catenin signalling.^35^ Further optimization *via* the introduction of additional hydrogen-bond donors onto the aryl ring afforded 11 (WCJ-394), which exhibits similar inhibitory activities against PRMT1 (IC_50_ = 1.2 μM) PRMT4 (IC_50_ = 1.6 μM) and PRMT8 (IC_50_ = 5.7 μM), and selectively lowers cellular ADMA without altering SDMA levels, thereby inhibiting the migration of human lung adenocarcinoma A549 cells.^36^ Exploration of a previously unexploited lipophilic auxiliary sub-pocket produced thiazole derivatives such as 12 (ZJG51) and 13 (ZJG58). These compounds retain the Asp84-anchoring ethylenediamine motif and exhibit PRMT1 IC_50_ values of 11.8 and 7.1 μM, respectively, with negligible PRMT5 cross-reactivity (2.5% and 6% PRMT5 inhibition at 10 μM, respectively) and low-micromolar antiproliferative and anti-migratory activities in HeLa cells.^37^ More recently, compound 14 (YH-4) was reported as a potent, substrate-competitive PRMT1 inhibitor (IC_50_ = 4.1 μM), displaying 6-fold and 7-fold selectivity over PRMT6 and PRMT8, respectively, and >20-fold selectivity over PRMT3, PRMT4, PRMT5, and PRMT7, and capable of reducing cellular ADMA and H4R3me2a levels, inducing apoptosis, and synergizing with paclitaxel in TNBC xenograft models.^38^

A second major class of substrate-competitive PRMT1 inhibitors relies on amidine and diamidine motifs designed to mimic the guanidinium group of substrate arginines (Chart 2B). Structure-based screening and scaffold hopping from antiprotozoal drugs identified 15 (stilbamidine, PRMT1 IC_50_ = 15.2 μM) as an early inhibitor with moderate selectivity for PRMT1 over PRMT5 (3-fold) and PRMT6 (10-fold), together with approximately 26-fold selectivity over PRMT4,^39^ followed by more potent diamidines such as 16 (furamidine), which displays a PRMT1 IC_50_ of 9.4 μM and >10-fold selectivity over PRMT4, PRMT5 and PRMT6.^40^ Drug-repurposing approaches using 3D shape and feature similarity (SHAFTS) subsequently revealed alkyl bis(oxy)dibenzimidamides 17 (pentamidine) and 18 (hexamidine) as substrate-competitive PRMT1 ligands (IC_50_ = 13.2 μM and 5.9 μM, respectively) that suppress proliferation in PRMT1-overexpressing cancer cells. Both compounds displayed limited inhibitory activity toward PRMT5 while maintaining activity against PRMT3 and PRMT4, suggesting a degree of selectivity for type I PRMTs over type II PRMTs.^41^ Pyrimidine–benzimidamidine derivatives, such as compound 19, further validate the amidine group as a versatile arginine mimic, yielding low-micromolar PRMT1 inhibition (IC_50_ = 2.0 μM) and non-competitive kinetics *versus* SAM, corroborating a substrate-channel mechanism. In addition, compound 19 displayed a marked preference for PRMT1, with negligible activity against PRMT5 and PRMT6 (IC_50_ > 100 μM) and only moderate inhibition of PRMT4 (IC_50_ = 10 μM).^42^

Diverse discovery strategies continue to expand the chemical diversity of PRMT1 ligands (Chart 2C). Virtual screening approaches led to compound 20 (NS-1), a naphthalene-sulfonate structurally related to compound 1 (Chart 1) that inhibits PRMT1 with an IC_50_ of 12.7 μM and exhibits approximately 3-fold selectivity over PRMT6 and ∼160-fold selectivity over PRMT4, while retaining comparable potency toward PRMT3, suggesting a preference for type I PRMTs. Detailed kinetic and biophysical assays indicate that 20 acts as a substrate-competitive inhibitor, primarily by interfering with peptide substrate binding rather than directly occupying the core catalytic pocket.^43^

Mixed SAM/substrate-competitive binding modes have been observed for arylsulfonamides, including compound 21 (C-7280948, IC_50_ = 12.8 μM), which occupies SAM-adjacent regions while maintaining key contacts with aromatic residues (Met146, His45) in the peptide channel.^32^ Optimization yielded compound 22 (TC-E-5003), a bis-chloroacetyl dapsone derivative acting as a partially covalent PRMT1 inhibitor in the low-micromolar range (IC_50_ = 1.5 μM), and displaying selectivity over PRMT4 and the lysine methyltransferase SET7/9. The compound also exhibited antiproliferative activity in hormone-dependent malignancies, including androgen-dependent prostate cancer (LNCaP) and oestrogen receptor-positive breast cancer cells (MCF-7).^44^

Virtual screening also identified compound 23 (DCLX069), a piperazine-based scaffold that inhibits PRMT1 with micromolar potency (IC_50_ = 17.9 μM). Compound 23 displayed a favourable selectivity profile, showing minimal inhibition of PRMT4 and PRMT6 even at 100 μM, consistent with a marked preference for PRMT1. Although its biochemical activity is modest, 23 successfully suppresses proliferation across breast, liver, and acute myeloid leukaemia (AML) cell lines, indicating that targeting the SAM-binding site can translate into productive cellular effects. Crucially, the compound displays a favourable physicochemical profile, including good membrane permeability and ligand efficiency, consistent with its moderate molecular flexibility. These features position 23 as an appealing starting point for further medicinal chemistry optimization.^45^

Very recently, artificial intelligence and machine learning (AI/ML)-aided screenings identified benzodiazepinones as putative scaffolds for selective PRMT1 inhibition. Compound 24 was predicted to bind stably to PRMT1 based on molecular docking and molecular dynamics (MD) simulations, displaying favourable *in silico* druglike and ADMET properties. However, experimental validation of its inhibitory activity or selectivity remains to be reported.^46^

Collectively, these studies highlight the diverse chemical structures explored for PRMT1 inhibition, emphasizing the strategic utility of guanidinium-mimicking motifs and target interactions within the peptide-binding channel to achieve selectivity. Despite these advances, many compounds still display moderate potency or limited selectivity, and translating biochemical inhibition into robust cellular activity remains a challenge. Further optimization guided by high-resolution structural details and rigorous biological validation will be essential to advance these scaffolds toward effective therapeutics.

### Dual PRMT1/PRMT4 binders

**Chart 3:**
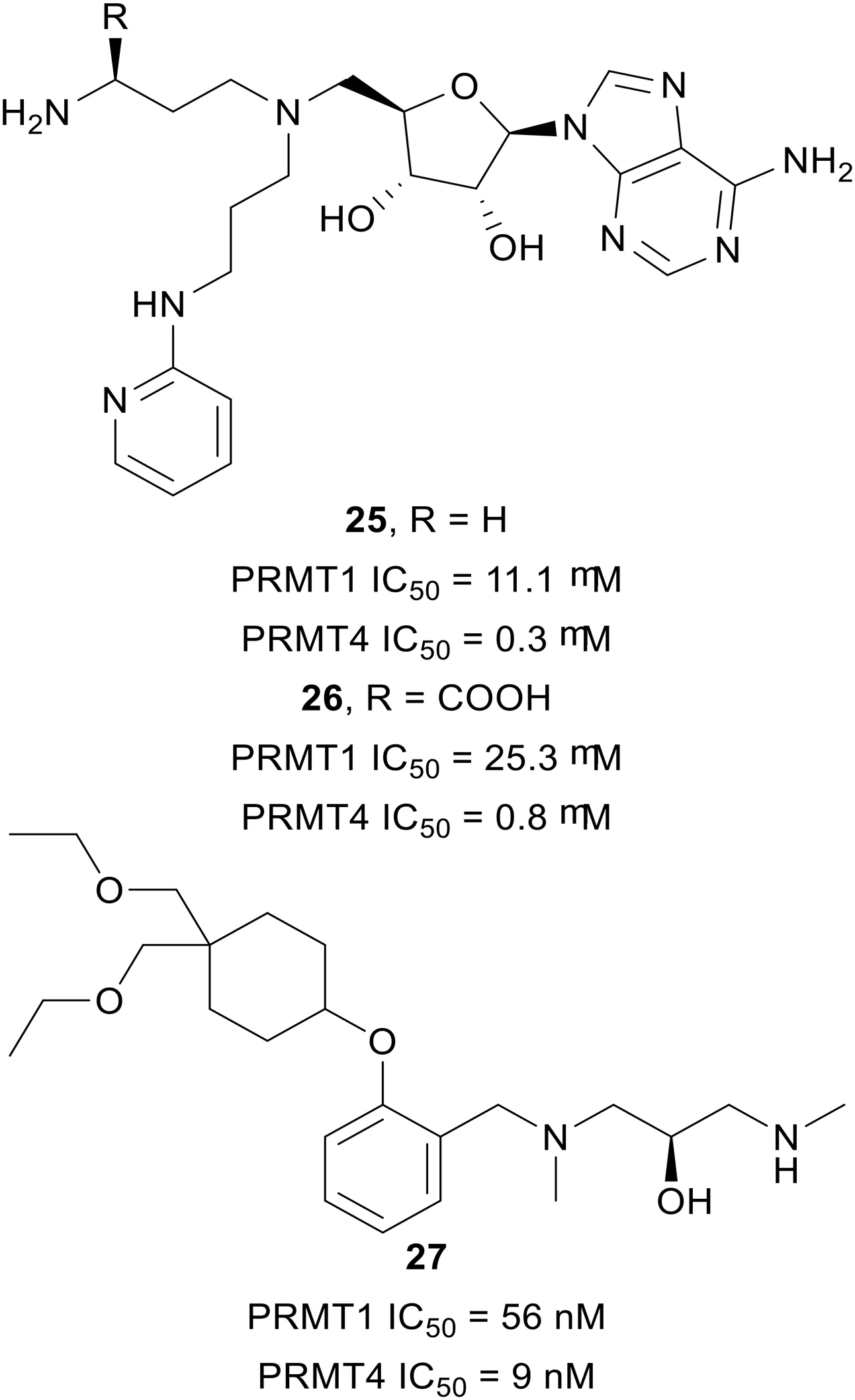
Representative structures of dual PRMT1/PRMT4 inhibitors. All IC_50_ values were determined in biochemical assays unless otherwise stated. Compounds 25 and 26 incorporate a 2-aminopyridine moiety acting as a guanidinium mimic, enabling binding within the substrate recognition site and conferring a marked preference for PRMT4 over PRMT1. Compound 27 illustrates further scaffold diversification toward dual-target engagement.

In the context of polypharmacology, simultaneously targeting distinct PRMT isoforms can be exploited to optimize therapeutic outcomes. Consequently, the development of dual target-directed ligands has emerged as an attractive strategy (Chart 3).^47^ Co-targeting PRMT1 and PRMT4 (CARM1) is of particular interest, as both isoforms are frequently co-overexpressed in several aggressive malignancies and cooperate to drive oncogenic transcriptional programs. Compounds 25 and 26, reported by Dreveny and co-workers in 2020, represent early examples of dual PRMT1/PRMT4 inhibitors, demonstrating that both enzymes can be successfully co-targeted within a single molecular scaffold. Consistent with their structure-based design, these compounds incorporate a 2-aminopyridine moiety as a guanidine mimic, which contributes to preferential binding toward PRMT4 over PRMT1. Accordingly, they display a marked selectivity for PRMT4, with IC_50_ values of 11.1 and 0.3 μM for compound 25, and 25.3 and 0.8 μM for compound 26, respectively.

Structural analysis revealed that this selectivity profile arises from subtle differences within the substrate-binding pocket—most notably involving Asn265 in PRMT4 *versus* Tyr160 in PRMT1. These distinct residues influence the conformation of a catalytically essential, neighboring glutamate residue and, in turn, modulate ligand binding. These structural findings highlight how minor variations in active site architecture among closely related PRMT isoforms can be strategically exploited to achieve tailored selectivity.^48^

In 2024, chemical modification of pan-type I PRMT inhibitors containing an ethylenediamine pharmacophore yielded compound 27, a dual inhibitor exhibiting nanomolar potency against both PRMT4 (IC_50_ = 9 nM) and PRMT1 (IC_50_ = 56 nM), alongside a favourable predicted druglikeness profile. Compound 27 is cell-active, induces apoptosis, and impairs the proliferation of colorectal cancer cells while selectively suppressing PRMT4-dependent methylation.^49^ Collectively, these results indicate that dual PRMT1/PRMT4 modulation is synthetically achievable and may offer a practical, single-agent alternative to combinatorial pharmacology in specific PRMT-driven oncological settings.

### PRMT3

PRMT3 is a type I arginine methyltransferase originally identified as a PRMT1-interacting protein.^50^ It is characterized by a unique N-terminal C_2_H_2_ zinc-finger domain that mediates binding to ribosomal protein S2 (rpS2), its principal physiological substrate. Consistent with this interaction, PRMT3 localizes predominantly within the cytoplasm, associates with free ribosomal subunits, and regulates translational processes—a function evolutionarily conserved from yeast to plants.^51^ Although *Prmt3* knockout mice are viable, they display transient developmental growth delays, indicating that while PRMT3 is not strictly essential for survival, it contributes significantly to physiological homeostasis.^52^

Beyond its ribosome-associated functions, PRMT3 methylates nuclear substrates such as c-Myc^53^ and HIF-1α.^54^ It also catalyses histone H4R3 asymmetric dimethylation^55^ and topoisomerase TOP3B methylation *in vitro*,^56^ supporting a broader regulatory scope.

Aberrant expression or altered activity of PRMT3 has been documented in several malignancies. In colorectal cancer, PRMT3 promotes tumorigenesis by stabilizing c-Myc and enhancing HIF-1α methylation, thereby reinforcing oncogenic and hypoxia-adaptive transcriptional programs.^53,54^ In breast cancer, PRMT3-driven H4R3me2a deposition activates endoplasmic reticulum stress signalling, supporting cell proliferation and metastatic potential.^57^ Moreover, in hepatocellular carcinoma, PRMT3 directly methylates lactate dehydrogenase A (LDHA) at Arg112, enhancing its enzymatic activity and promoting tumour cell proliferation; this axis has also been implicated in resistance to oxaliplatin-based chemotherapy.^58^ Beyond oncology, PRMT3 is overexpressed in non-alcoholic fatty liver disease (NAFLD), where it directly interacts with liver X receptor α (LXRα) in a methylation-independent manner, enhancing LXRα transcriptional activity and promoting hepatic lipid accumulation.^59^

Collectively, these findings establish PRMT3 as a multifunctional regulator of translational control, transcriptional adaptation and metabolic rewiring, whose context-dependent roles in tumorigenesis and metabolic disease provide a clear rationale for selective pharmacological targeting.

### PRMT3 inhibitors

Recent medicinal chemistry efforts have focused on developing selective PRMT3 inhibitors to enable rigorous chemical validation of PRMT3-dependent vulnerabilities. Early development strategies centred on substrate-site ligands that mimic peptide engagement within the arginine channel. Although these agents demonstrated robust inhibition, they often suffered from modest selectivity profiles and off-target toxicities, such as platelet depletion, limiting their translational potential.

These constraints prompted the exploration of noncanonical binding sites, leading to the identification of allosteric, or “metastable”, PRMT3 inhibitors. In this context, “metastable” refers to the ability of these ligands to bind and stabilize a transient or less-populated conformational state of the enzyme, modulating its activity through long-range conformational control rather than direct competition with the substrate.

**Chart 4:**
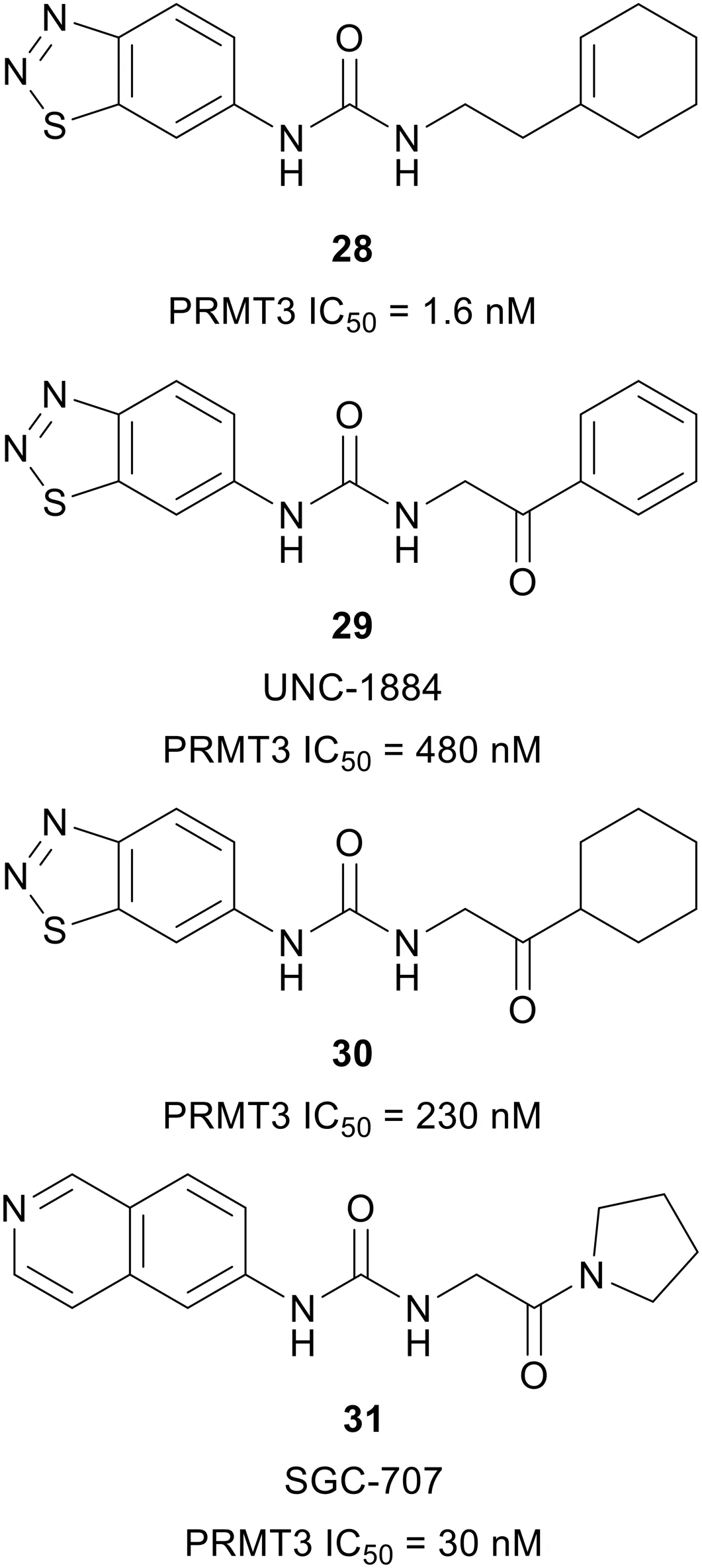
Representative structures of allosteric PRMT3 inhibitors based on the alkyl–heteroaryl urea scaffold. All IC_50_ values were determined in biochemical assays unless otherwise stated. Compounds 28–30 feature a benzothiadiazole core linked to a urea moiety, which enables binding at the PRMT3 dimer interface and perturbation of the αX helix, a key element for SAM engagement. Structural variations in the terminal substituents modulate potency and physicochemical properties. Compound 28 represents the first-in-class inhibitor of this series, while compound 31 (SGC-707) illustrates a more advanced analogue with improved potency and suitability as a chemical probe for selective PRMT3 inhibition.

To date, the most prominent class of selective PRMT3 inhibitors comprises the alkyl–heteroaryl ureas depicted in Chart 4. The first-in-class compound 28 was discovered *via* high-throughput screening and demonstrated micro-molar potency (IC_50_ = 1.6 μM). Biophysical analyses, including surface plasmon resonance (SPR) and steady-state kinetics, confirmed direct binding (*K*_D_ = 9.5 μM).

Crystallographic studies revealed an unexpected binding mode at the dimer interface that perturbs the αX helix, which is essential for productive SAM engagement. Structural analysis indicated key hydrogen-bonding interactions between the benzothiadiazole nitrogens and Thr466, while the urea linker forms essential hydrogen bonds and the cyclohexenylethyl moiety extends out of the binding pocket.

Importantly, compound 28 displayed a high degree of isoform selectivity, showing no detectable inhibition against other type I PRMTs (PRMT1, PRMT4 and PRMT8), no activity toward protein lysine methyltransferases, and only weak inhibition of PRMT5. This profile reflects the exploitation of a PRMT3-specific allosteric pocket rather than the highly conserved catalytic site. Despite these advantages, the compound suffered from poor metabolic stability, limiting its utility in cellular systems and highlighting the need for further optimization.^60^

To enhance affinity while preserving the metastable binding mode, the alkyl chain of the parent compound 28 was systematically modified. Derivatives 29 (UNC-1884) and 30, featuring an additional carbonyl group, showed improved potencies, with IC_50_ values of 480 nM and 230 nM, respectively. The X-ray co-crystal structure of PRMT3 in complex with compound 29 (PDB: 4HSG) confirmed allosteric engagement at the same atypical pocket, reinforcing the notion that PRMT3 harbours druggable dimer-interface cavities distinct from canonical substrate or SAM sites. Notably, compound 29 exhibited a highly selective profile, showing at least 40-fold selectivity over lysine methyltransferases G9a, GLP, and SUV39H2, while no measurable activity was detected against PRMT5 or a broader panel of epigenetic enzymes, including SETD7, SETD8, SETDB1, SUV420H1, SUV420H2, MLL1, SMYD2, SMYD3, DOT1L, and DNMT1. Collectively, these findings highlight the potential of exploiting the unique allosteric pocket of PRMT3 to achieve both high potency and remarkable selectivity.^61^

Replacement of the benzothiadiazole core with an isoquinoline ring system yielded compound 31 (SGC-707), which displayed high potency (IC_50_ = 30 nM; sub-100 nM affinity in biophysical assays) along with excellent isoform selectivity across panels of PRMTs, PKMTs, DNMTs, RNA methyltransferases, and non-epigenetic proteins. Structural insights attribute its enhanced potency to optimal packing of the isoquinoline ring system within the PRMT3 cavity and fine-tuned interactions of the pyrrolidinamide moiety with Lys392. Compound 31 achieved robust cellular target engagement, reducing the methylation of endogenous and exogenous H4R3 in HEK293 and A549 cells, and currently represents the most advanced preclinical PRMT3 chemical probe with favourable pharmacokinetic properties.^62^

### PRMT4/CARM1

PRMT4 (also known as coactivator-associated arginine methyltransferase 1, CARM1) has emerged as a compelling epigenetic drug target owing to its central involvement in transcriptional regulation and its heterogeneous expression across diverse tumour types.^63^ PRMT4 was originally identified through a yeast two-hybrid protein–protein interaction screen as a binding partner of GRIP1, a member of the p160 steroid receptor coactivator family, and was subsequently shown to possess intrinsic transcriptional coactivator activity.^64^ This dual role as both an enzyme and a transcriptional regulator underlies its designation as a coactivator-associated methyltransferase, positioning it as a key amplifier of signal-dependent gene expression.

Genetic studies have underscored the essential physiological functions of PRMT4. Germline deletion of *Prmt4* in mice is incompatible with postnatal survival, with PRMT4-null animals dying shortly after birth—a phenotype distinct from the early embryonic lethality observed upon the loss of PRMT1 or PRMT5.^65^ The generation of conditional *Prmt4* knockout models has enabled tissue-specific and temporal interrogation of PRMT4 function in adult organisms, while enzyme-dead knock-in models have provided critical mechanistic insights. These latter studies demonstrated that the biological functions of PRMT4 are largely dependent on its catalytic activity, with limited contribution from non-enzymatic scaffolding roles.^66^ This distinction is particularly relevant for predicting the *in vivo* consequences of small-molecule inhibitors that selectively target PRMT4 methyltransferase activity.

PRMT4 regulates transcriptional programs by methylating a diverse set of transcription factors and coactivators, including nuclear receptor complexes, SRC family members, p300/CBP, BRD4, and MED12.^67–70^ In addition, PRMT4 modulates the activity of several lineage-defining and oncogenic transcription factors, such as RUNX proteins, members of the NFI family, PAX7, and SOX9.^71–73^ Through these interactions, PRMT4 contributes to the control of differentiation, cell identity, and oncogenic transcriptional states, supporting the concept of “transcription factor addiction” in certain cancers where tumour cells become highly dependent on specific transcriptional programs for survival and proliferation.^74^

Beyond transcriptional regulation, PRMT4 plays a role in post-transcriptional gene control by modulating alternative pre-mRNA splicing.^75^ In this context, emerging evidence suggests that tumours harbouring mutations in RNA splicing factors may exhibit heightened sensitivity to PRMT4 inhibition, paralleling observations made for PRMT1 and PRMT5.^23^ Such genetic contexts may therefore provide predictive biomarkers for therapeutic response to PRMT4-targeted agents.

### PRMT4 inhibitors

Despite strong biological and genetic validation, the development of potent and selective PRMT4 inhibitors has proven challenging. A major obstacle has been the high degree of structural conservation within the SAM-binding cavity across PRMT family members, which imposes stringent constraints on achieving isoform selectivity.^27,76–81^ Progress over the past decade—driven by structure-based design, high-throughput screening, and chemoproteomic profiling—has nonetheless produced chemically diverse ligands showing low-nanomolar inhibition and robust target engagement in cells.^82,83^

**Chart 5:**
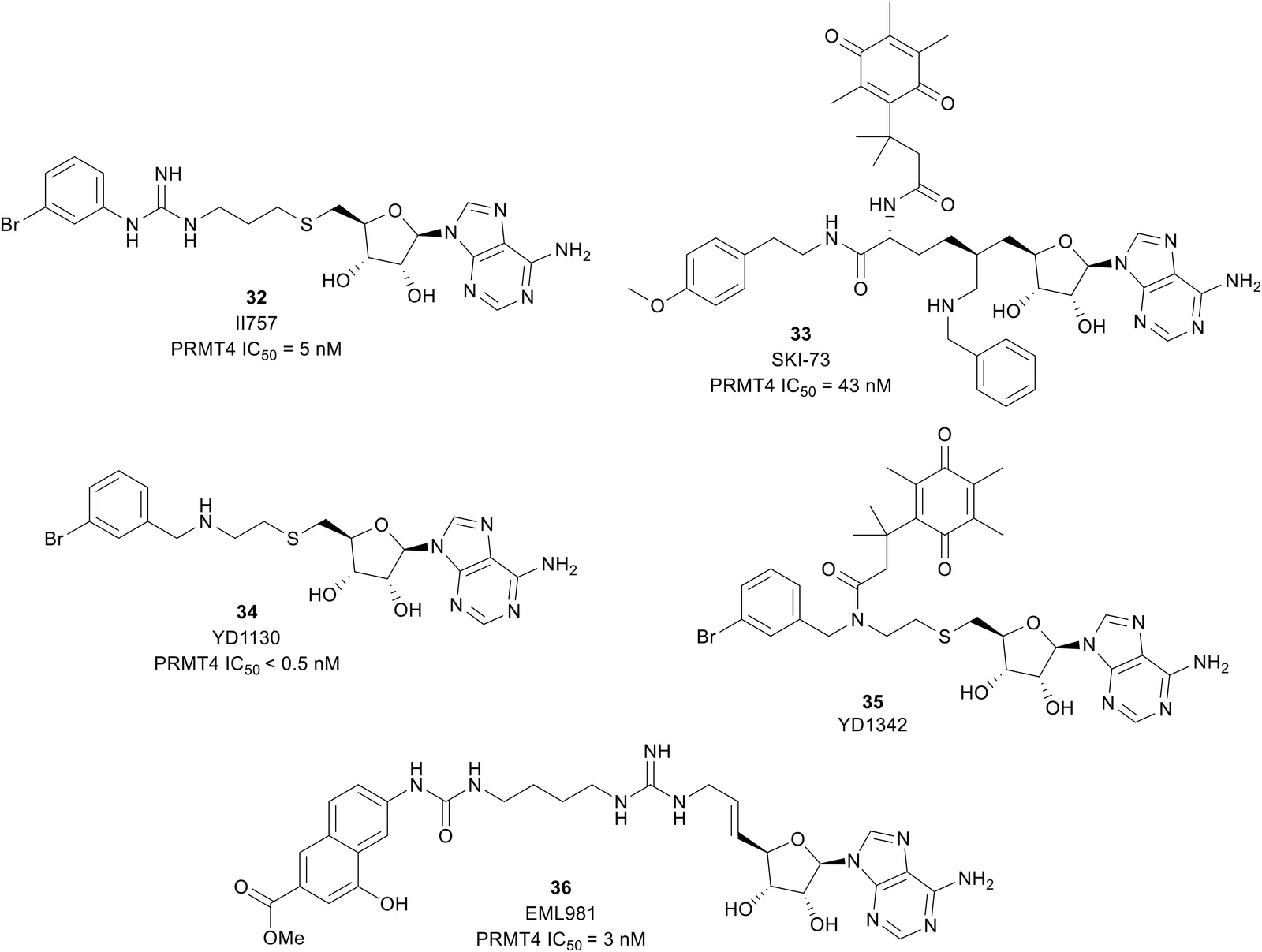
Representative structures of SAH/SAM-inspired PRMT4 inhibitors. All IC_50_ values were determined in biochemical assays unless otherwise stated. Compounds 32–35 are based on SAH/SAM-inspired scaffolds, with 32 showing high potency but limited selectivity, while prodrug strategies (33, 35) improved cellular activity. Compounds 34 and 35 exhibit sub-nanomolar potency and high (>1000-fold) selectivity over other PRMTs. Compound 36 (EML981) represents a structurally distinct, highly selective inhibitor derived from scaffold deconstruction approaches.

Historically, the majority of PRMT4 inhibitors have been developed as inhibitors derived from SAH/SAM-inspired scaffolds (Chart 5). These compounds combine a 5′-thioadenosine or related adenosine-based core, which engages the SAM-binding site, with guanidinium or amino acid-derived motifs that extend into the substrate-binding channel, thereby simultaneously mimicking both substrates of the methyltransferase reaction. Within this class, compound 32 (II757) displayed broad inhibitory activity across the PRMT family, with IC_50_ values ranging from 5 to 555 nM for eight PRMT isoforms and particularly high potency toward PRMT4 (IC_50_ = 5 nM). Although conceived as a bisubstrate-inspired ligand, kinetic analyses, together with molecular docking, indicated that its inhibitory activity primarily arises from occupation of the conserved SAM-binding cavity. The appended guanidino substituent contributes to target recognition but does not establish productive interactions within the substrate arginine-recognition channel. This binding mode explains both its broad activity across PRMT isoforms and its preference for PRMTs over other methyltransferase families, making 32 a valuable chemical probe and an attractive starting scaffold for the development of more selective derivatives.^84^

Subsequent medicinal chemistry efforts focused on metabolically activated prodrugs, leading to compound 33 (SKI-73, IC_50_ = 43 nM), which preferentially targeted invasive breast cancer subpopulations and displayed a favourable selectivity profile, being more than 10-fold selective over the remaining seven human PRMTs and 26 methyltransferases belonging to other enzyme classes. 33 acts as a cell-permeable prodrug that undergoes intracellular conversion into long-lived active metabolites that retain a SAM-competitive mode of inhibition, resulting in sustained target engagement and prolonged inhibition of PRMT4-dependent methylation programs associated with breast cancer invasiveness.^85^ More recently, an important advance emerged with 34 (YD1130) and its prodrug 35 (YD1342), which exhibited sub-nanomolar potency (IC_50_ < 0.5 nM) and over 1000-fold selectivity over other PRMTs. Mechanistically, these adenosine-derived inhibitors act through a bisubstrate mode of action. Moreover, the ability of 35 to suppress the methylation of PRMT4 substrates, including BAF155, translated into pronounced antitumour efficacy in animal models.^86^

Complementary scaffold mining has expanded the chemical diversity of PRMT4 inhibitors beyond adenine-mimetic templates. Using a deconstruction/reconstruction strategy, 36 (EML981) was identified as a highly potent bisubstrate PRMT4 inhibitor (IC_50_ = 3 nM), exhibiting >100-fold selectivity over PRMT1, PRMT3, PRMT5, PRMT6, PRMT7, and PRMT8, while its overall cellular permeability remained modest.^77^

**Chart 6:**
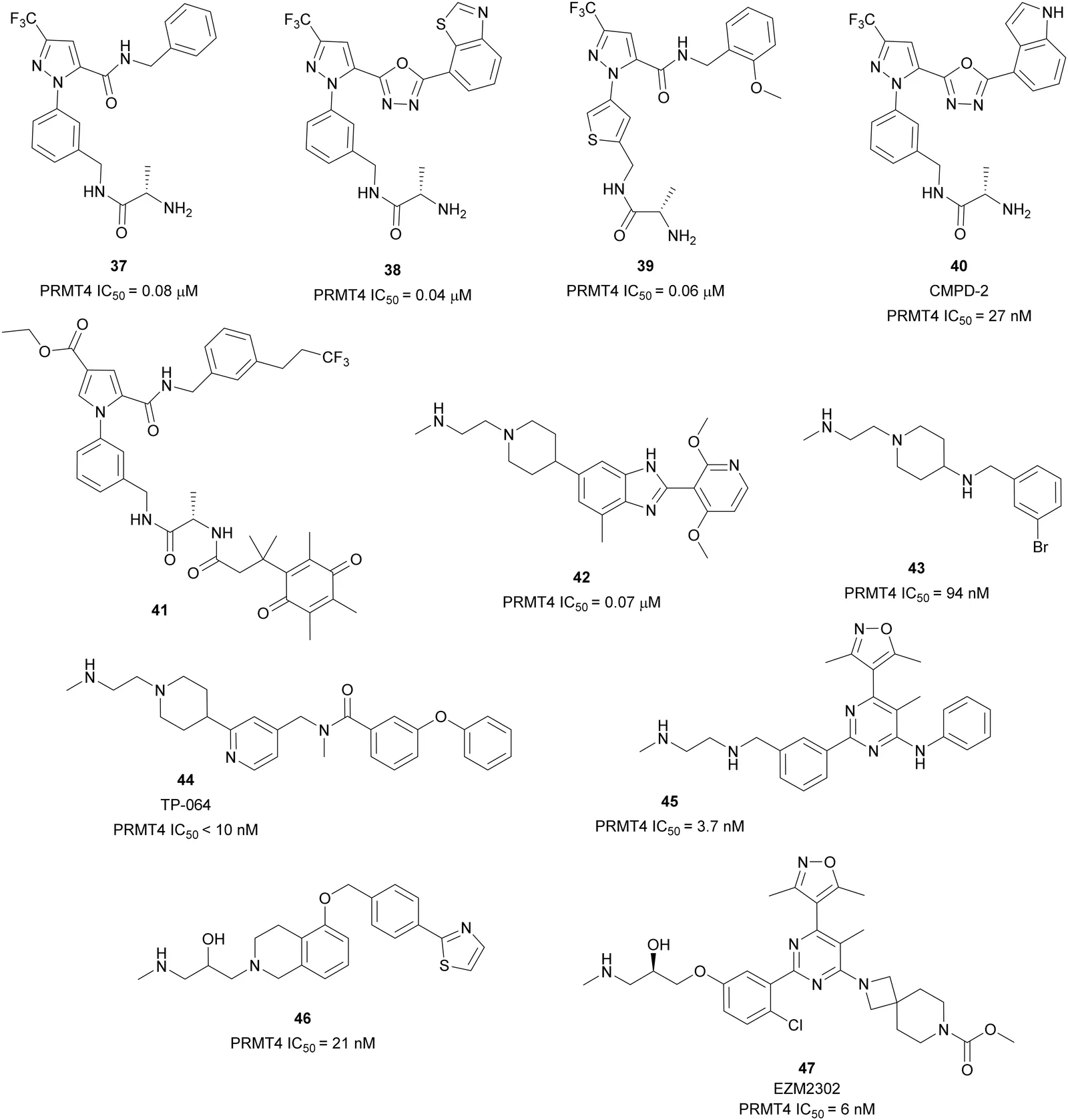
Representative structures of advanced PRMT4 inhibitors developed through structure-based design and optimization strategies. All IC_50_ values were determined in biochemical assays unless otherwise stated. These compounds illustrate the evolution from early SAM-mimetic scaffolds toward more refined chemotypes with improved potency, selectivity, and cellular activity. Structural modifications around the core pharmacophore enabled enhanced engagement within the cofactor-binding site and improved pharmacological profiles, supporting their use as chemical probes and potential therapeutic candidates.

Compounds capable of acting as substrate-competitive PRMT4 inhibitors are depicted in Chart 6. They belong to different chemical classes, but all feature an arginine-mimicking motif. Screening campaigns identified the alanine amide moiety as a valuable arginine mimic for the development of potent and selective PRMT4 inhibitors. Iterative SAR campaigns yielded pyrazole derivatives with good potency and favourable physicochemical properties, including compounds 37 (IC_50_ = 0.08 μM), 38 (IC_50_ = 0.04 μM),^87^39 (IC_50_ = 0.06 μM), and 40 (CMPD-2, IC_50_ = 27 nM).^88^ However, limited activity in cell-based assays was reported due to poor cellular uptake. To address this issue, subsequent refinements introduced pyrrole moieties and prodrug elements (*e.g.*, compound 41) to improve exposure and cellular methylation readouts.^89^ Several PRMT4 inhibitors exploit an ethylenediamine core as an arginine mimic.^90^ For example, substrate-competitive compound 42 (IC_50_ = 0.07 μM) exhibited remarkable selectivity for PRMT4,^91^ showing little activity against PRMT1 and PRMT3 (IC_50_ > 25 μM for both enzymes), corresponding to more than 350-fold selectivity over these closely related type I PRMTs. Subsequently, compound 43 exhibited nanomolar potency against PRMT4 (IC_50_ = 94 nM) and a favourable selectivity profile, being approximately 20-fold more potent toward PRMT4 than PRMT6, while showing negligible inhibition of PRMT1, PRMT3, PRMT5, PRMT7, and PRMT8. Kinetic analyses indicated a noncompetitive inhibition pattern with respect to both SAM and the peptide substrate. Nevertheless, structural and mechanistic evidence suggests that the compound binds to the substrate-recognition pocket of PRMT4 rather than to a distinct allosteric site.^92^ Several derivatives within this class showed high potency and remarkable cellular activity. Compound 44 (TP-064, IC_50_ < 10 nM) exhibited potent and highly selective inhibition of PRMT4, displaying more than 100-fold selectivity over the other PRMT family members, with only moderate activity against the closely related PRMT6 (IC_50_ = 1.3 μM) and weaker inhibition of PRMT8 (IC_50_ = 8.1 μM). In addition, no significant activity was detected against a panel of 24 lysine or DNA methyltransferases at concentrations up to 10 μM. Kinetic analyses revealed a noncompetitive inhibition pattern with respect to both SAM and the peptide substrate, consistent with binding to the substrate-recognition pocket. Biologically, 44 produced target-dependent antiproliferative effects in multiple myeloma cells and synergized *in vivo* with inhibitors of the lysine methyltransferase DOT1L.^93^ Subsequently, compounds 45 and 46 showed excellent potency (IC_50_ = 3.7 nM and 21 nM, respectively). Both act through substrate-pocket recognition and display excellent PRMT4 selectivity (>100-fold over the remaining PRMT family members), translating into antitumour activity in both hematologic malignancies and solid tumours.^94,95^

A major milestone was reached by exploiting a 1-amino-3-phenoxy-propan-2-ol scaffold as an arginine mimic. Indeed, compound 47 (EZM2302 or GSK3359088), an orally bioavailable nanomolar inhibitor (IC_50_ = 6 nM), combined high selectivity over the remaining PRMT family members and other methyltransferases, with compelling ADME profiles. Structural and kinetic studies demonstrated that 47 binds within the substrate arginine-binding pocket, exhibiting a noncompetitive inhibition pattern with respect to both SAM and the peptide substrate and forming a long-lived inhibitory PRMT4–SAH complex. These properties translated into robust activity in preclinical models of multiple myeloma and solid tumours.^96^

Despite the breadth of preclinical tool compounds and convincing *in vivo* data across multiple scaffolds, no PRMT4-targeted agent has yet entered clinical development or obtained IND clearance from regulatory agencies. In fact, all known PRMT4 inhibitors remain confined to the discovery and preclinical domains, and none have progressed into human safety, pharmacokinetic, or efficacy studies.^97^ Representative molecules—including 47—continue to serve as high-quality chemical probes to dissect PRMT4 biology in tumourigenesis, lineage plasticity, epigenetic regulation, and immune responses. However, achieving clinically viable pharmacokinetic properties, sufficient oral bioavailability, and robust solid-tumour efficacy remain critical challenges.^94–96,98^

Beyond medicinal chemistry, the lack of clinical translation likely reflects the biological complexity of PRMT4 itself. Unlike PRMT5, whose activity is closely associated with well-defined genetic vulnerabilities such as MTAP deletion (see PRMT5 section below), PRMT4 functions in a highly context-dependent manner and regulates multiple transcriptional programs that vary substantially across tumour types. Consequently, identifying patient populations most likely to benefit from PRMT4 inhibition remains challenging. Moreover, potential adaptive signalling mechanisms and the absence of validated predictive biomarkers may further limit the clinical development of otherwise highly potent and selective inhibitors. These considerations suggest that future progress will depend not only on further optimization of pharmacokinetic properties, but also on improved biological stratification and rational combination strategies.

Looking ahead, rational lead optimization, biomarker-based patient stratification, and combination strategies—particularly with immunotherapies, where PRMT4 blockade has been reported to enhance T-cell activity and sensitize tumours to checkpoint inhibition—are expected to accelerate the translation of PRMT4 inhibitors toward clinical investigation.^74,97^ Continued integration of prodrug design, structure-guided medicinal chemistry, and systems biology approaches will likely be required to convert potent biochemical inhibitors into therapeutically deployable agents.

### PRMT6

PRMT6 was identified through sequence homology with other type I PRMTs^99^ and is ubiquitously expressed with predominant nuclear localization. It primarily catalyses the asymmetric dimethylation of histone H3 at Arg2 (H3R2me2a) and methylates non-histone substrates, such as DNA polymerase β, thereby modulating chromatin organization and DNA-associated processes.^100,101^

Although PRMT6 is not required for mouse viability or development, its loss triggers premature senescence in embryonic fibroblasts, highlighting its role in governing the proliferative lifespan of cells.^102^

Conversely, transgenic overexpression of PRMT6 promotes mammary gland hyperproliferation and cooperates with oncogenic drivers such as HER2 to accelerate tumour growth, supporting a context-dependent pro-oncogenic function.^103^

At the mechanistic level, PRMT6 regulates gene expression through the deposition of H3R2me2a, which antagonizes H3K4 trimethylation and suppresses the transcription of tumour suppressor genes, including *p21* (*WAF1*) and *p16* (*INK4A*).^104^ This chromatin mark also impairs the recruitment of UHRF1 and DNMT1, contributing to alterations in DNA methylation patterns that can be restored upon PRMT6 depletion or inhibition.^105^ Dysregulated PRMT6 expression has been reported in gastric and lung cancers, where it promotes aggressive behaviour through epigenetic silencing^106^ and metabolic reprogramming *via* the enhanced activity of 6-phosphogluconate dehydrogenase and α-enolase.^107^ In addition, PRMT6 contributes to the regulation of genes involved in differentiation and stemness, further highlighting its role in cell fate determination.^108,109^

### PRMT6 inhibitors

**Chart 7:**
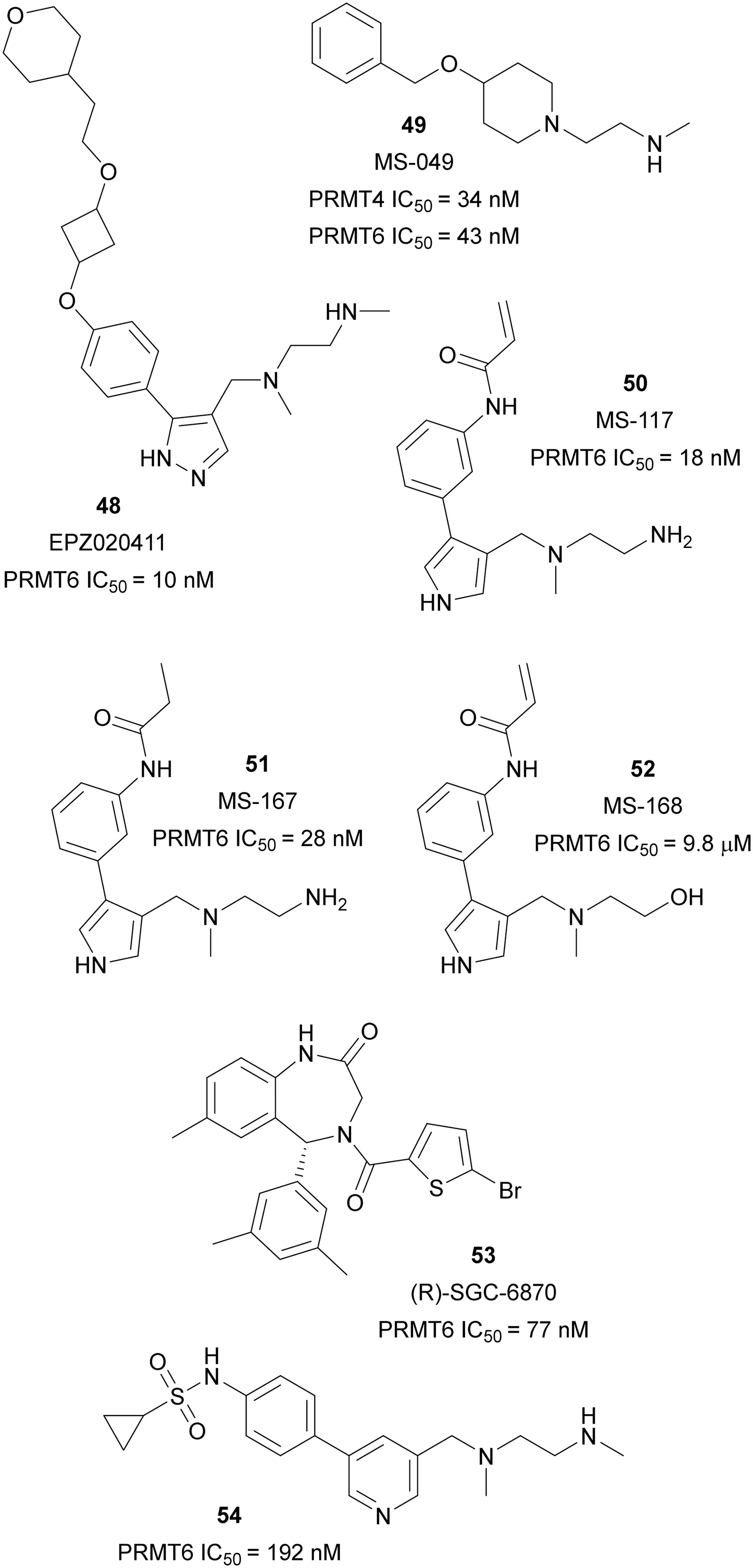
Representative structures of PRMT6 inhibitors spanning substrate-competitive and allosteric chemotypes. All IC_50_ values were determined in biochemical assays unless otherwise stated. Compounds 48 (EPZ020411) and 49 (MS-049) exemplify early substrate-competitive inhibitors featuring arginine-mimicking motifs, often associated with limited isoform selectivity. Covalent inhibitor 50 (MS-117) and related analogues 51 (MS-167) and 52 (MS-168) illustrate structure-based optimization targeting Cys50, highlighting the role of electrophilic warheads and key binding functionalities in modulating potency and mechanism. Compound 53 (SGC-6870) represents a highly selective allosteric PRMT6 inhibitor based on a benzodiazepinone scaffold, while compound 54 exemplifies further diversification of PRMT6-targeting chemotypes.

Despite its established pro-tumourigenic activities, PRMT6 has yet to yield a selective, single-target clinical candidate. Current clinical-grade pharmacological engagement of PRMT6 relies largely on pan-type I PRMT inhibitors that concurrently inhibit PRMT1 and related isoforms,^30^ thereby obscuring the specific *in vivo* contributions of PRMT6. Moreover, its extranuclear functions, including potential roles in intercellular signalling and tumour microenvironment modulation, remain poorly defined and require deeper mechanistic validation. Nevertheless, sustained medicinal chemistry efforts over the past decade have produced a chemically diverse portfolio of PRMT6 inhibitors (Chart 7). This array, encompassing substrate-pocket binders, dual-target ligands, covalent modifiers, and emerging allosteric chemotypes, collectively reinforces the tractability of PRMT6 as a viable epigenetic target.

The first selective small-molecule inhibitors of PRMT6 were substrate-competitive ligands exploiting an ethylenediamine core as an arginine mimic. The first reported derivative, compound 48 (EPZ020411), featured an aryl–pyrazole scaffold and showed potent PRMT6 inhibition (IC_50_ = 10 nM). Crystallographic studies revealed that the inhibitor binds within the substrate arginine-binding pocket, where the ethylenediamine moiety establishes an extensive hydrogen-bonding network with catalytic residues while coexisting with SAH in the SAM-binding site. Importantly, it displayed a measurable degree of selectivity over closely related type I PRMTs, with IC_50_ values of 119 nM and 223 nM against PRMT1 and PRMT8, respectively, and >100-fold selectivity over other PRMT isoforms and protein methyltransferases. However, despite favourable subcutaneous bioavailability in rats (∼66%), poor oral permeability has limited its *in vivo* utility.^110^

Subsequent structure-based optimization expanded the repertoire of substrate-directed PRMT6 inhibitors while underscoring the challenge of achieving strict isoform selectivity, owing to the high structural conservation of the substrate-binding pocket across type I PRMTs. For instance, compound 49 (MS-049) emerged as a potent dual PRMT4/PRMT6 inhibitor (IC_50_ = 34 and 43 nM, respectively), while maintaining >300-fold selectivity over PRMT1 and PRMT3 and showing no activity toward PRMT5 or PRMT7.^111^ These findings illustrate how subtle differences within the substrate-binding site can be exploited to preferentially target closely related PRMT isoforms, although complete discrimination remains difficult. Consistent with this concept, clinically advanced pan-type I inhibitors, such as 6 (Chart 1), also inhibit PRMT6 together with PRMT1 and PRMT8, providing *in vivo* validation of PRMT6 pharmacology despite the inability to resolve isoform-specific contributions.^30^

A pivotal mechanistic insight was the identification of Cys50 within the PRMT6 substrate channel. As a unique nucleophilic residue absent in other type I PRMTs, Cys50 enabled the design of electrophile-containing ligands for site-specific covalent modification of PRMT6.^112^

Starting from the co-crystal structure of PRMT6 with 3 (PDB: 5E8R), a type I PRMT inhibitor, and exploiting an acrylamide moiety as an electrophilic warhead, compound 50 (MS-117) was discovered. It is characterized by potent and irreversible PRMT6 inhibition (IC_50_ = 18 nM), together with marked selectivity over other PRMT isoforms, particularly PRMT3 (∼167-fold) and PRMT4 (∼27-fold).^112^ The propionamide analogue 51 (MS-167) retained comparable potency (IC_50_ = 28 nM) and a similar selectivity profile, remaining approximately 70-fold and >500-fold selective over PRMT4 and PRMT3, respectively, although only 5–6-fold selective over the closely related PRMT8 and PRMT1. Unlike 50, however, it behaved as a reversible inhibitor, confirming the role of the acrylamide moiety in covalent engagement. In contrast, substitution of the terminal primary amine with a primary alcohol in compound 52 (MS-168) resulted in a dramatic loss of activity (IC_50_ ≈ 9.8 μM), despite preservation of the electrophilic warhead.^112^ This finding highlights the critical contribution of non-covalent interactions—particularly those mediated by the amine group—for proper binding orientation and productive covalent modification. Collectively, these compounds exhibited low cytotoxicity and represent valuable chemical probes for investigating PRMT6 biology.

As with PRMT1 inhibitors (see compound 24), benzodiazepinones have been disclosed as putative scaffolds for developing selective PRMT6 inhibitors. Compound 53 ((*R*)-SGC-6870) inhibited PRMT6 with nanomolar potency (IC_50_ = 77 nM) and displayed pronounced enantioselectivity, as the corresponding (*S*)-enantiomer was essentially inactive (IC_50_ > 50 μM), representing a >600-fold difference in potency. This marked enantioselectivity underscores the stringent stereochemical requirements of the allosteric pocket. Compound 53 also exhibited an outstanding selectivity profile, showing >130-fold selectivity over all other PRMT family members and no detectable inhibition of 21 protein lysine methyltransferases, three DNA methyltransferases, one RNA methyltransferase, or a broad panel of non-epigenetic targets at concentrations up to 10 μM. Mechanistically, 53 showed time-dependent, noncompetitive inhibition consistent with a metastable allosteric mechanism, characterized by slow-binding kinetics rather than covalent modification. Crystallographic studies (PDB: 6W6D) revealed binding to a unique induced pocket distinct from both the substrate and SAM-binding sites, generated by conformational rearrangement of a flexible loop (Gly158-Met166). The ligand is stabilized by hydrogen bonds between the diazepine lactam and the backbone of Gly158 and Gly160, along with T-shaped π–π interactions involving the thiophene and Tyr159, and the dimethylphenyl moiety and Trp156. In cellular assays, 53 reduced H3R2me2a and H4R3me2a levels with submicromolar potency (IC_50_ ≈ 0.9 and 0.6 μM, respectively) while exhibiting minimal cytotoxicity, establishing it as the first high-quality, selective allosteric chemical probe for PRMT6.^113^

Recent efforts revisited the substrate channel with improved selectivity constraints. Specifically, pyrimidinyl-substituted ligands like 54 displayed submicromolar biochemical potency (IC_50_ = 192 nM) together with a markedly improved selectivity profile, exhibiting >25-fold selectivity over PRMT1 and PRMT8 and >50-fold selectivity over PRMT3, PRMT4, PRMT5, and PRMT7. Mechanistic studies demonstrated a non-SAM-competitive mode of inhibition, consistent with binding to the substrate-recognition pocket rather than the SAM-binding site. Molecular modeling further suggested that the enhanced selectivity arises from a unique hydrogen-bond interaction with the PRMT6-specific residue Glu49, highlighting how subtle differences within the substrate channel can be exploited for precision targeting of PRMT6.^114^

Collectively, PRMT6 inhibitors now encompass substrate-competitive, SAM-competitive, dual-target, covalent, allosteric, and degradation modalities, reflecting an increasingly diversified medicinal chemistry landscape. While no selective single-target PRMT6 inhibitor has advanced into clinical evaluation, the availability of potent biochemical and cellular probes—including 48, 51, and 53—has accelerated biological investigation and clarified the therapeutic rationale for PRMT6 in cancer.^110,112,113^ Outstanding challenges include improving oral bioavailability, achieving clean isoform selectivity, resolving PRMT6-dependent vulnerabilities, and evaluating combination strategies—particularly with DNA damage response inhibitors, immunotherapies, and metabolic modulators. Future progress will likely depend on integrating structural biology, mechanism-aware medicinal chemistry, and biomarker stratification to convert PRMT6 from a validated chemical probe target into a clinically actionable epigenetic dependency.

### PRMT8

PRMT8 is the closest homolog of PRMT1 within the type I PRMT family but differs significantly in its tissue distribution and subcellular localization.^115^ Unlike other PRMTs, PRMT8 contains a unique N-terminal myristoylation motif that promotes plasma membrane association, and its expression under physiological conditions is largely restricted to the brain and nervous system.^115^ Genetic studies have shown that PRMT8-null mice are viable and fertile, displaying no major developmental defects, although neurological abnormalities such as limb-clasping behaviour indicate a specific role in neural function.^116^

Distinct from other PRMT family members, PRMT8 exhibits dual enzymatic activities: in addition to its type I arginine methyltransferase function, it possesses intrinsic phospholipase activity capable of hydrolysing phosphatidylcholine.^116^ Both catalytic functions appear to be required for proper neuronal development and synaptic maturation. Mechanistically, PRMT8 regulates synaptic actin dynamics and dendritic spine maturation through the methylation of substrates such as G3BP1 and the modulation of Rac1–PAK1 signalling pathways.^117^

Although predominantly brain-specific,^118^ PRMT8 expression has also been detected in embryonic^119,120^ and cancer stem cells,^121^ as well as in several tumour types, including breast, ovarian, cervical, and gastric cancers.^122^ Its functional role in cancer remains context-dependent: PRMT8 has been implicated in stemness maintenance through the stabilization of factors such as Sox2, yet clinical correlations vary across tumour types, suggesting that PRMT8 may function either as a context-specific driver or as a proliferation-associated marker.^121^ In addition, PRMT8 contributes to neuroprotective and anti-inflammatory processes, including the regulation of microglial activation and cerebral ischemia responses.^123^

Collectively, PRMT8 represents a brain-enriched, multifunctional PRMT with distinct structural and catalytic properties, whose biological roles extend from synaptic development to context-dependent tumour biology.

To date, no high-quality, selective small-molecule inhibitors of PRMT8 have been described. Nevertheless, several ethylenediamine-based compounds originally developed against other type I PRMTs—such as 3 and 49—exhibit measurable inhibitory activity toward PRMT8. This cross-reactivity is consistent with the high degree of sequence and structural conservation shared by PRMT8 with PRMT1 and PRMT6, which inherently limits opportunities for traditional isoform-selective inhibition within the canonical active site.

## Type II PRMTs

### PRMT5

The predominant type II PRMT family member is PRMT5, which is responsible for the majority of cellular SDMA marks in mammals.^124^ Unlike type I PRMTs, this protein exerts its function as part of high-molecular-weight multi-protein complexes that invariably contain methylosome protein 50 (WDR77, also known as MEP50). This association enhances both protein stability and methyltransferase activity. In addition, MEP50 participates in recruiting partner proteins and substrates to the catalytic domain of PRMT5. Thus, the hetero-octameric PRMT5:MEP50 assembly constitutes the functional biological module, which can associate with additional cellular proteins in a context-dependent manner to form larger multicomponent complexes that specifically methylate a diverse range of substrates.^125^

PRMT5 preferentially methylates arginine residues sandwiched between two neighbouring glycines within characteristic “GRG” (Gly-Arg-Gly) motifs.^126^ Arginine methylation occurs on histone substrates, including H2R3, H3R2, H3R8, and H4R3, driving or repressing gene expression depending on the specific residue modified.^127–129^ PRMT5 also catalyses the symmetric dimethylation of arginine residues on non-histone substrates, such as RNA-binding Sm proteins, the 40S ribosomal protein S10 (RPS10), and the transcription factor E2F1.^126,130–132^ Through these targets, PRMT5 is implicated in a diverse set of cellular processes, including cell cycle progression, cell proliferation, and the DNA damage response.^133–135^

Increasing evidence reveals that PRMT5 plays a critical role in regulating tumour cell proliferation and transformation, acting as an oncogene in many human cancers. In most cases, elevated levels of the protein correlate strongly with poor prognosis.^136–140^ Interestingly, while *Prmt5* knockdown or depletion reduces cell proliferation in certain malignancies (*e.g.*, lung cancer),^133^ it can either accelerate or reduce growth in metastatic melanoma cell lines. These findings suggest that cell lineage and genetic background are critical determinants in predicting the therapeutic value of targeting PRMT5.^141^

Beyond cancer, PRMT5 is involved in cardiovascular and inflammatory events. For example, PRMT5 has been observed to be an important regulator of myocardial hypertrophic signalling, suggesting a role in modulating cardiomyocyte hypertrophy.^142^ In addition, PRMT5-mediated arginine methylation of NF-κB enhances the expression of CXCL10, a chemokine involved in recruiting Th1 effector cells to sites of infection and inflammation.^143^ In the context of inflammation, PRMT5 can act as a positive regulator, contributing to the induction of several NF-κB target genes as well as genes encoding cytokines, chemokines, and growth factors, including IL-1α, IL-8, and TNF receptor-associated factor 1 (TRAF-1).^144^ PRMT5 also plays a critical role in antibody responses, as well as in the development, activation, and proliferation of B cells.^145^ Furthermore, PRMT5 was found to be an autoantibody target of the autoimmune response in patients with systemic sclerosis, and high levels of anti-PRMT5 antibodies are significantly associated with the presence of interstitial lung disease in rheumatoid arthritis.^145,146^

### PRMT5 inhibitors

The broad involvement of PRMT5 in numerous pathological conditions has prompted the development of a vast number of inhibitors. With several compounds currently undergoing clinical evaluation, PRMT5 is the most advanced isoform in the PRMT family from a clinical translation standpoint. PRMT5 inhibitors are classified into distinct classes based on their binding sites or specific mechanisms of action. Most of these compounds belong to the “first-generation inhibitors”, which include SAM-competitive, substrate-competitive, and dual-binding inhibitors. Despite the extensive efforts devoted to the discovery of PRMT5 modulators and their advancement into clinical trial, many of these compounds have been limited by dose-limiting toxicities, with adverse events including neutropenia, thrombocytopenia, and anaemia. Moreover, clinical outcomes have highlighted the difficulty of translating promising preclinical activity into therapeutic benefit, reflecting the context-dependent role of PRMT5 in cancer cell survival beyond its canonical function in arginine methylation.

In an attempt to overcome these drawbacks, “second-generation” methylthioadenosine (MTA)-cooperative inhibitors have gained significant attention due to their unique mechanism. These compounds take advantage of the intrinsically reduced activity of PRMT5 in cancer cells lacking *MTAP*, thereby enhancing their sensitivity to further pharmacological inhibition of PRMT5 and enabling tumour-selective targeting.

#### SAM-competitive inhibitors

**Chart 8:**
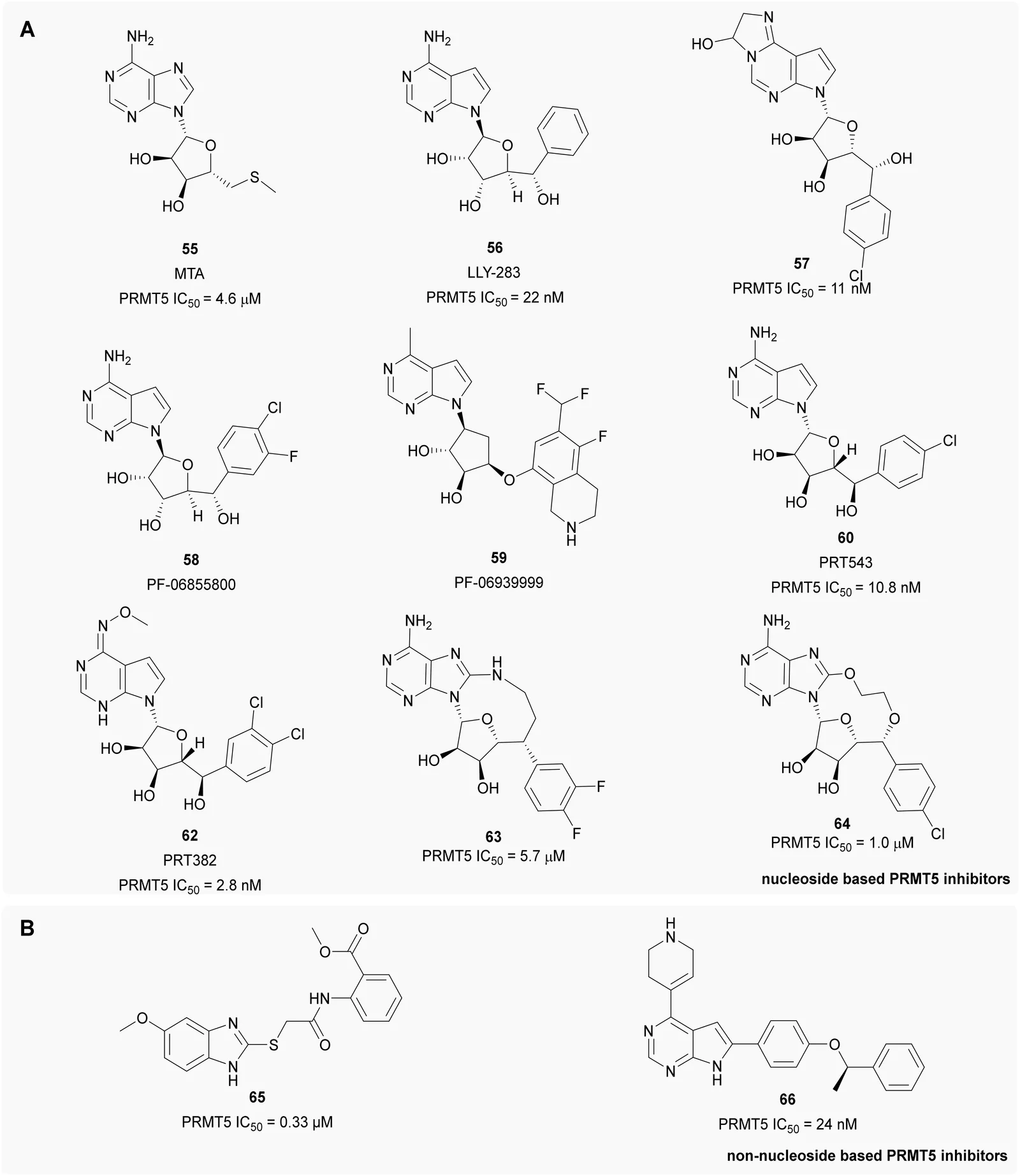
Representative structures of SAM-competitive PRMT5 inhibitors, classified according to their core chemical features. All IC_50_ values were determined in biochemical assays unless otherwise stated. (A) Nucleoside-based inhibitors (compounds 55–64) bearing motifs adenosine moiety designed to occupy the SAM binding pocket and compete with the SAM. (B) Non-nucleoside PRMT5 inhibitors (compounds 65 and 66), targeting the SAM binding pocket without containing an adenosine mimic scaffold.

As with all PRMTs, diverse SAM-competitive inhibitors have been developed (Chart 8). These molecules typically feature a common adenosine core designed to occupy the cofactor binding pocket and compete with endogenous SAM. However, the remarkable structural similarity of these compounds, combined with the highly homologous nature of the SAM-dependent methyltransferase domain across the family, often yields compounds that successfully target the protein but lack clear isoform selectivity.^147^

Nevertheless, the discovery of 5′-methylthioadenosine (55, MTA) as a potent and selective PRMT5 inhibitor provided a valuable starting point for developing SAM-mimetic compounds with high PRMT5 selectivity. Despite its strong structural resemblance to SAM, 55 selectively inhibits PRMT5 with low micro-molar potency (IC_50_ = 4.6 μM) without affecting the activity of 18 other histone methyltransferases tested, including diverse PRMTs such as PRMT1, PRMT4 and PRMT8 (IC_50_ > 60 μM). As expected, the crystal structure (PDB: 4GQB) revealed that the adenine and ribose moieties of 55 adopt the same binding mode as SAM within the PRMT5:MEP50 complex with an H4 peptide. However, the compound induces a subtle reorganization of the SAM binding pocket. This shift facilitates unique interactions between residues specific to PRMT5 (Tyr334-Lys333) and Glu435, a highly conserved catalytic residue across the PRMT family, partially explaining the selectivity of the compound.^148^

Considering these findings, different SAM-mimetic inhibitors have been developed (Chart 8A). In 2018, Eli Lilly and the Structural Genomics Consortium identified compound 56 (LLY-283) as a selective PRMT5 inhibitor with no significant activity against other arginine methyltransferases, including closely related PRMT7 isoform as well as type I PRMTs. The compound showed high inhibitory activity *in vitro* against the PRMT5:MEP50 complex (IC_50_ = 22 nM) and a very strong affinity against the protein target (*K*_D_ = 6 nM). Compound 56 inserts into the SAM binding pocket, as revealed by the crystal structure with the PRMT5:MEP50 complex (PDB: 6CKC). The adenine moiety establishes hydrogen bonds with the side-chain carboxylate of Asp419 and the main-chain amide of Met420. On the other hand, the ribose forms a pair of hydrogen bonds with the side chain of Glu392, as well as a hydrogen bond with the side chain of Tyr324. Interestingly, SAM competition assays did not support a classical competitive mode of action with respect to the SAM; instead, the compound appears to act noncompetitively with respect to both the peptide substrate and SAM. Compound 56 showed good antiproliferative activity across several cancer cell lines. Administration in A375 mouse xenografts resulted in significant tumour growth inhibition compared to vehicle-treated controls,^149^ and similar results were observed upon co-administration of 56 in mice bearing ovarian tumour xenografts resistant to cisplatin, where it significantly enhanced the cytotoxic efficacy of the chemotherapy drug.^150^

Starting from 56 as a parent compound and leveraging structural insights from compound 55 in complex with PRMT5:MEP50, a series of covalent PRMT5 inhibitors was developed. The co-crystal structure of 55 in complex with PRMT5:MEP50 highlighted the presence of a cysteine residue (Cys449) within the active site. Because this residue is unique to PRMT5 and absent in other PRMT family members, it represents a strategic target for developing selective covalent inhibitors. Therefore, a series of hemiaminals was developed that convert to reactive aldehydes under physiological conditions and engage Cys449 to form covalent adducts. Among these, compound 57 emerged as a promising covalent PRMT5 inhibitor, showing high inhibitory potency against the PRMT5:MEP50 complex (IC_50_ = 11 nM) and good selectivity over PRMT1 and PRMT4, with no detected activity on these isoforms.^151^

This success prompted the development of several SAM-competitive PRMT5 inhibitors structurally analogous to compound 56 (compounds 58–64). Pfizer Inc. developed the tool compound 58 (PF-06855800, *K*_i_ = 11 pmol L^−1^) which was subsequently optimized into compound 59 (PF-06939999), possessing high inhibitory activity (*K*_i_ values fell below the assay's lower limit of quantitation of <5 pmol L^−1^) and drug-like physicochemical properties. Both compounds demonstrated high selectivity across a panel of methyltransferases with compound 58 showing at least one million-fold selectivity for PRMT5 over the enzymes of the panel, while compound 59 showed no activity above 20% inhibition over protein methyltransferase and kinases. Antitumour effects were demonstrated *in vivo* by orally administering the inhibitor to mouse xenograft models of non-small-cell lung cancer (NSCLC).^152^ Based on these premises, compound 59 entered a phase I clinical study in 2019 (NCT03854227) in patients with advanced or metastatic head and neck squamous cell carcinoma (HNSCC), NSCLC, or oesophageal, endometrial, cervical, or bladder cancer. Overall, 59 demonstrated a tolerable safety profile, with 4 of 28 patients reporting dose-limiting toxicities. However, clear predictive biomarkers were not identified, indicating that additional preclinical studies are required to establish reliable markers for patient stratification. In 2022, the study was terminated following a strategic evaluation within the Pfizer oncology portfolio.^153^

Prelude Therapeutics Inc. disclosed the clinical candidate 60 (PRT543), a nanomolar inhibitor (IC_50_ = 10.8 nM) that showed high selectivity when tested against a broad panel of methyltransferases, with residual enzyme activity remaining above 60% for PRMT3 and PRMT4 and above 90% for PRMT1, PRMT6 and PRMT8. It effectively suppressed the methyltransferase activity of the PRMT5:MEP50 complex by competing with SAM and MTA, but not with the substrate. Compound 60 produced marked inhibition of cell proliferation across a wide panel of cancer cell lines and significantly reduced tumour growth in AML xenograft models. In 2019, 60 entered a phase I dose-escalation/expansion trial in patients with advanced cancers who had exhausted available treatment options (NCT03886831). Efficacy in monotherapy was modest, and diverse adverse effects including anemia and thrombocytopenia emerged.^154^ The same company developed 61 (PRT811), a potent (IC_50_ = 3.9 nM) and brain-penetrant PRMT5 inhibitor which displayed high selectivity over other isoforms, with residual activity ranging from 50% to 60% for PRMT7 and exceeding 60% for all type I PRMTs.^155^ The compound entered into a phase I clinical trial in subjects with advanced cancers and high-grade gliomas (NCT04089449).^156^ Compound 62 (PRT382) is another potent inhibitor (IC_50_ = 2.8 nM) of the human PRMT5:MEP50 complex, demonstrating an high selectivity against 38 methyltransferases as well as against PRMT7 and type I PRMTs (IC_50_ > 1 μM) and ability in reducing SDMA in the Granta-519 lymphoma cell line (IC_50_ = 27 nM). The compound demonstrated anti-proliferative efficacy across leukemia and lymphoma cell lines, alongside low clearance and high oral bioavailability in mice, rats, and dogs.^157^ In 2022, a series of cyclonucleosides was reported as PRMT5 inhibitors. Compounds 63 (9-membered ring) and 64 (10-membered ring) inhibited PRMT5-mediated monomethylation of H4R3 with IC_50_ values of 5.7 μM and 1.0 μM, respectively. Moreover, the co-crystal structure of PRMT5:MEP50 in complex with 63 (PDB: 7U30) confirmed its binding directly within the SAM binding pocket. However, no selectivity assessment against other PRMTs has been reported.^158^

In addition to nucleoside inhibitors, several attempts have been made to develop highly potent and selective non-nucleoside PRMT5 inhibitors targeting the SAM binding pocket (Chart 8B). In 2017, a structure-based virtual screening campaign and subsequent hit optimization yielded compound 65, characterized by a 5-methoxybenzimidazole core. The compound inhibited PRMT5 by competing with SAM with an IC_50_ of 0.33 μM and showed broad selectivity against PRMT7 and type I PRMTs (IC_50_ values greater than 100 μM) as well as antiproliferative effects against MV4–11 cells. Further studies investigated its antitumour activity in a cellular context related to the inhibition of PRMT5-mediated SmD3 methylation.^159^ In 2022, a structure-aided drug design approach led to the identification of a novel series of non-nucleoside SAM-competitive inhibitors. Among them, 66 selectively inhibited PRMT5 (IC_50_ = 24 nM), strongly reducing cellular proliferation across diverse cancer cell lines as a direct consequence of PRMT5 inhibition. The selectivity was evaluated in a different biochemical assay in which 66 showed an IC_50_ of 47 nM, a weak activity against PRTM1 (IC_50_ = 3 μM) and no activity against PRMT4 (IC_50_ up to 20 μM). Moreover, 66 showed good PK properties and robust activity in suppressing tumour growth in an A375 tumour xenograft model.^160^

#### Substrate-competitive inhibitors

**Chart 9:**
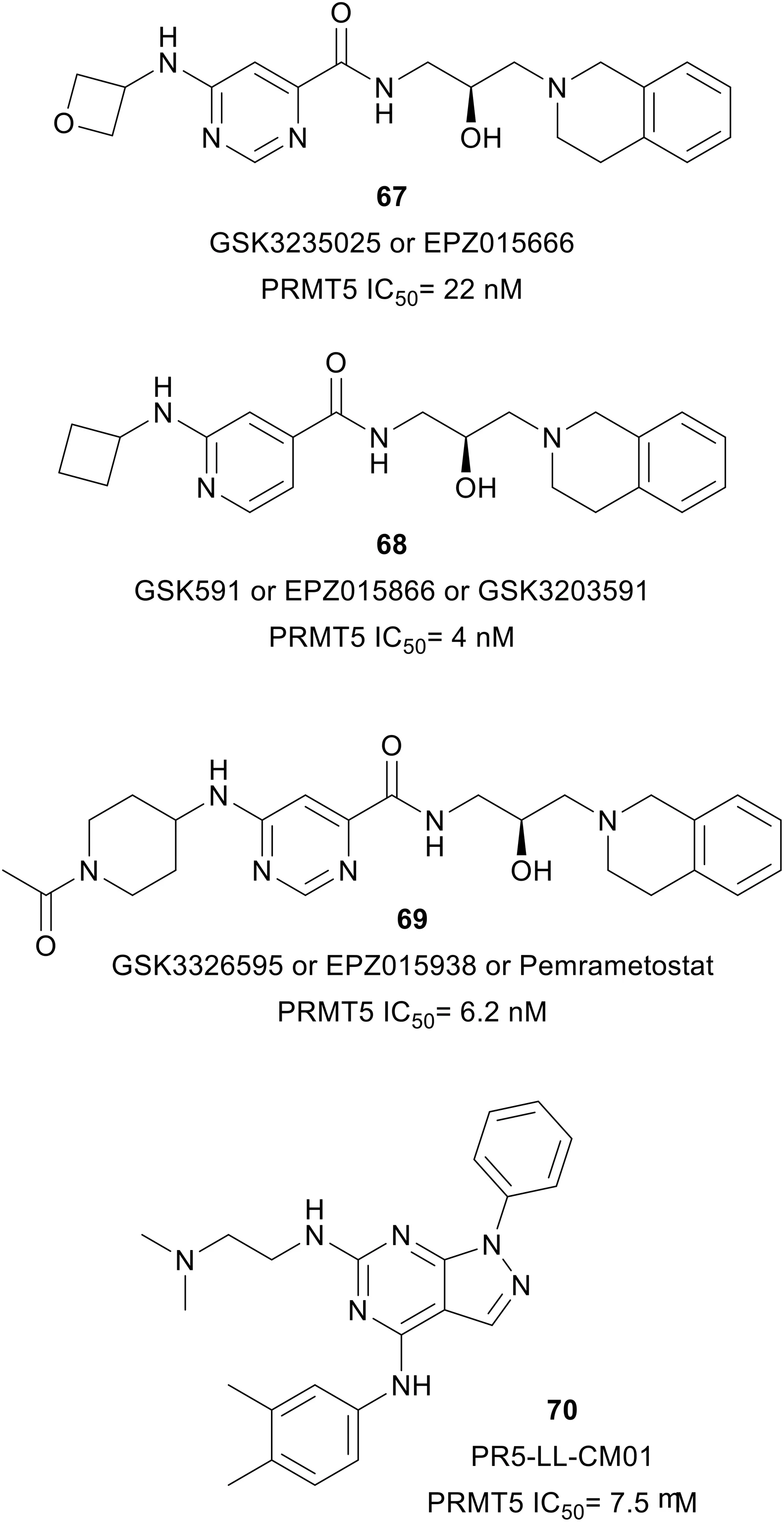
Representative structures of substrate-competitive PRMT5 inhibitors. All IC_50_ values were determined in biochemical assays unless otherwise stated. Compounds 67 (GSK3235025 or EPZ015666), 68 (EPZ015866 or GSK591 or GSK3203591) and 69 (GSK3326595 or EPZ015938 or pemrametostat) illustrate structures featuring a key THIQ-amino alcohol motif. Compound 70 (PR5-LL-CM01) represents a substrate competitive inhibitor characterized by a pyrazolo-pyrimidine scaffold.

In 2015, the tetrahydroisoquinoline (THIQ) derivative 67 (GSK3235025 or EPZ015666) was disclosed as the first highly cell-potent (IC_50_ = 22 nM) and orally bioavailable substrate-competitive, SAM-uncompetitive inhibitor of PRMT5 (Chart 9).

It demonstrated remarkable selectivity for PRMT5 when profiled against a panel of 20 protein methyltransferases, including other PRMTs, with no inhibition up to the maximum tested concentration of 50 μM. The crystal structure of 67 within the PRMT5:MEP50 complex in the presence of the cofactor SAM (PDB: 4X60) showed that the compound inserts into the substrate-binding site, including the pocket occupied by the peptide substrate backbone, supporting its non-competitive mechanism. Interestingly, a cation–π interaction between the phenyl ring of the THIQ moiety and the partially positively charged methyl group of SAM emerged as a critical stabilizing feature. Cellular target engagement of PRMT5 was validated by CETSA and by treating various mantle cell lymphoma (MCL) cell lines. Oral administration of 67 in multiple MCL xenograft models demonstrated dose-dependent antitumour activity as a direct consequence of PRMT5 inhibition.^161^

Subsequent medicinal chemistry optimization yielded inhibitors with improved cellular potency and enhanced drug-likeness. Initially, the key THIQ-amino alcohol scaffold was retained while exploring different substitutions on the alkoxyphenyl ring. Data obtained confirmed the importance of the cation–π interaction established by the THIQ core and led to the identification of the close analogue 68 (EPZ015866, GSK591, or GSK3203591) (Chart 9), which showed inhibitory activity in the low nanomolar range (IC_50_ = 4 nM) alongside >2000-fold selectivity for PRMT5 over a panel of 20 other protein methyltransferases, although its activity against PRMT9 was not evaluated.^162^ Further optimization led to compound 69 (GSK3326595, EPZ015938, or pemrametostat) (Chart 9). Analysis of the inhibition mechanism and crystallographic evidence proved that 69 is an uncompetitive inhibitor with respect to SAM and a competitive inhibitor for the peptide substrate, interacting with the protein in a binding mode similar to 67. The compound was found to be a potent inhibitor of the PRMT5:MEP50 complex with an IC_50_ of 6.2 nM and displayed high selectivity (>4000-fold) over all other methyltransferases tested, including PRMT9, the other type II PRMT. Cellular activity profiles across a panel of hematologic and solid cancer lines revealed that compounds 68 and 69 exert antitumour effects that vary based on tumour type, showing maximum sensitivity in lymphoma (including MCL and diffuse large B-cell lymphoma [DLBCL]), breast, and multiple myeloma cell lines, with growth IC_50_ values in the low nanomolar range. Interestingly, compound 69 exhibited reduced efficacy in a *p53*-mutant MCL xenograft model compared to the *p53* wild-type Z-138 model, demonstrating that PRMT5 inhibition functionally affects the *p53*/MDM4 axis. This strong correlation between anti-proliferative effects and differential sensitivity underscores the importance of identifying specific patient subpopulations most likely to benefit from PRMT5-targeted therapies.^163^

In 2016, 69 entered a first-in-human, open-label, multicentre, phase I clinical trial to evaluate safety, tolerability, and preliminary efficacy (NCT02783300) both as monotherapy and in combination with pembrolizumab across different cancer types. Overall, modest antitumour activity was detected with monotherapy. However, notable exceptions were observed in adenoid cystic carcinoma, sarcoma, and non-Hodgkin lymphoma at the recommended phase II dose, where preliminary signs of efficacy were reported. Similarly, the combination cohort with pembrolizumab demonstrated an overall modest response rate.^164^ In 2018, compound 69 was evaluated in an open-label, multicentre, phase I/II study (NCT03614728) to assess safety, tolerability, pharmacokinetics, pharmacodynamics, and clinical activity in patients with relapsed and/or refractory myelodysplastic syndrome (MDS), chronic myelomonocytic leukaemia (CMML), or hypoproliferative acute myeloid leukaemia (AML). Results obtained in part I of the study confirmed limited clinical activity in monotherapy within the patient population tested. The study was subsequently terminated, halting part II, which had been planned to evaluate 69 in combination with 5-azacitidine.^165^ In 2021, a phase II window-of-opportunity trial in early-stage breast cancer (NCT04676516) was initiated and completed a year later, though clinical results remain unreported.^166^

In 2017, an AlphaLISA high-throughput screen led to the discovery of the PRMT5 inhibitor 70 (PR5-LL-CM01), structurally characterized by a pyrazolo-pyrimidine core (Chart 9). The compound inhibits PRMT5 with an IC_50_ of 7.5 μM and shows no detectable activity—or at least a 10-fold higher IC_50_—against other PRMTs. Structural modelling experiments showed that 70 interacts with Glu444, a critical residue for catalytic activity. Notably, the residues involved in the binding interactions are distributed across the Rossmann fold and β-barrel domains, which constitute the core catalytic domain of the protein housing the binding sites for SAM and its substrates. The inhibitor was approximately 10- to 15-fold more potent than 67 in pancreatic ductal adenocarcinoma (PDAC) and colorectal cancer (CRC) models.^167^

#### Dual SAM-substrate competitive inhibitors

**Chart 10:**
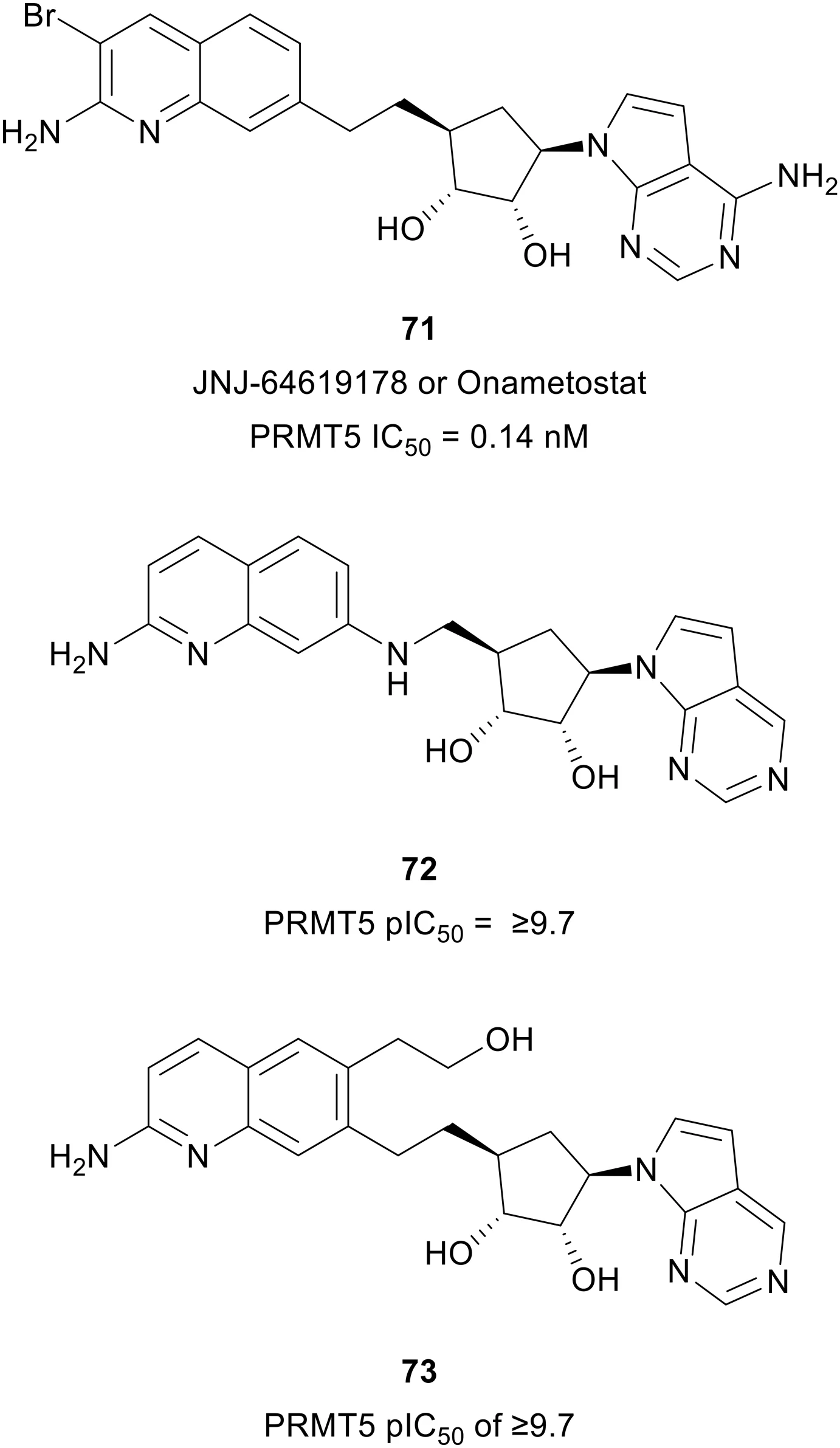
Representative structures of dual SAM-substrate binder PRMT5 inhibitors. All IC_50_ (or pIC_50_) values were determined in biochemical assays unless otherwise stated. Compound 71 (JNJ-64619178 or onametostat) demonstrated a dual SAM-substrate inhibition which led to an extended stabilization of PRMT5. It was also used as a starting point to achieve gut restriction inhibition, resulting in compounds 72 and 73 containing an unsubstituted C4 pyrrolo[2,3-*d*]pyrimidine nucleobase identified as metabolic soft spot to favour hepatic metabolism and achieve a high degree of gut restriction.

In 2017, compound 71 (JNJ-64619178 or onametostat, Chart 10) was disclosed as a potent PRMT5 inhibitor with a *K*_i,app_ of 0.77 nM, IC_50_ of 0.14 nM (ref. 168) and a high binding affinity (*K*_D_ < 0.63 nM), proving highly selective against a broad panel of PRMTs as well as lysine and DNA methyltransferases, achieving more than 80% inhibition of the PRMT5/MEP50 complex at 10 μM while inhibiting the closely related PRMT1 and PRMT7 by less than 15%. The co-crystal structure with the PRMT5:MEP50 complex revealed non-covalent binding within the SAM-binding domain, extending the bromo-aminoquinoline moiety toward the substrate-binding pocket and allowing a hydrogen bond with the catalytic Glu444 residue. This dual SAM-substrate inhibition led to a long residence time and extended stabilization of PRMT5. These effects are translated well into cells, demonstrating that short exposure is sufficient to efficiently inhibit the target.

Furthermore, the sustained inhibitory effects of 71 were observed *in vivo*, as continuous daily dosing in mice produced efficacy comparable to intermittent dosing schedules, suggesting that optimized regimens could be deployed in clinical trials to reduce bone marrow toxicities associated with PRMT5 inhibition.^169^ A year after its disclosure, 71 entered a phase I, open-label, multicenter study to evaluate its safety and identify a recommended phase II dose in patients with advanced malignant solid tumours or non-Hodgkin lymphomas carrying lower risk of myelodysplastic syndromes (NCT03573310). Compound 71 showed a manageable dose-dependent toxicity profile, with thrombocytopenia identified as the most critical collateral effect. Nevertheless, beyond moderate efficacy in patients with adenoid cystic carcinoma (ACC), overall clinical responses were modest.^170^

Compound 71 was also shown to induce high toxicity in adenomatous polyposis coli (APC) loss-of-function (LOF) colon organoids compared to wild-type organoids. Considering that APC-LOF is regarded as an early disease-initiating event in colorectal cancer development, compound 71 was selected as a starting point for developing gut-restricted PRMT5 inhibitors. To achieve gut restriction and minimize systemic PRMT5 exposure, a dual strategy was employed to reduce passive absorption while enhancing systemic clearance. This was achieved by incorporating an aldehyde oxidase-sensitive metabolic “soft spot”, favouring rapid hepatic metabolism while remaining stable in the intestinal mucosa. To identify a suitable metabolic soft spot, several analogues with high turnover in human hepatocytes were analysed, revealing that a pyrrolo[2,3-*d*]pyrimidine nucleobase bearing an unsubstituted C4 position was a successful structural feature. The resulting compounds 72 and 73 (Chart 10) showed robust PRMT5:MEP50 inhibitory activity (pIC_50_ of ≥9.7 for both compounds), inducing apoptosis in human colon organoids and demonstrating *in vivo* pharmacodynamic colon toxicity in mice without signs of systemic on-target toxicity. Compound 72 was tested across a variety of methyltransferases including PRMT7 and type I PRMTs and does not achieve more than 20% inhibition for any of them. Moreover, 72 proved effective in polyp-bearing mice, confirming that local concentrations from a poorly permeable compound are sufficient to exert the desired pharmacological response in gastro-intestinal restricted contexts.^171^

#### MTA-cooperative PRMT5 inhibitors

Results from the majority of clinical trials revealed that most first-generation PRMT5 inhibitors are associated with dose-limiting toxicities, primarily related to neutropenia, thrombocytopenia, and anaemia. Moreover, available data underscore the challenges of translating preclinical efficacy into clinical success, pointing to a complex interplay between PRMT5 activity and cancer cell survival that extends beyond simple arginine methylation and varies across tumour types.^164^ In this scenario, alternative strategies to target PRMT5 have been explored to achieve higher selectivity and a safer therapeutic profile. One such approach involves the development of MTA-cooperative PRMT5 inhibitors.

Functional genomic studies have revealed that approximately 10% to 15% of tumours are characterized by homozygous deletion of the *MTAP* gene due to its chromosomal proximity to the tumour suppressor locus *CDKN2A*. This co-deletion is particularly common in aggressive cancers, such as pancreatic cancer, non-small-cell lung cancer, and glioblastoma. Under normal conditions, MTAP catalyses the conversion of 55 (MTA) into adenine and methionine. In contrast, loss of MTAP leads to the intracellular accumulation of 55, which competes with SAM for binding to PRMT5. Consequently, PRMT5 activity is intrinsically reduced in *MTAP*-null cells, rendering them hypersensitive to further pharmacological inhibition of PRMT5 compared with cells harbouring an intact *MTAP* gene.^172^ This evidence has paved the way for a second generation of PRMT5 inhibitors designed to achieve selective PRMT5 inhibition by preferentially targeting *MTAP*-null tumour cells while sparing *MTAP*-proficient normal tissues.^172^

**Chart 11:**
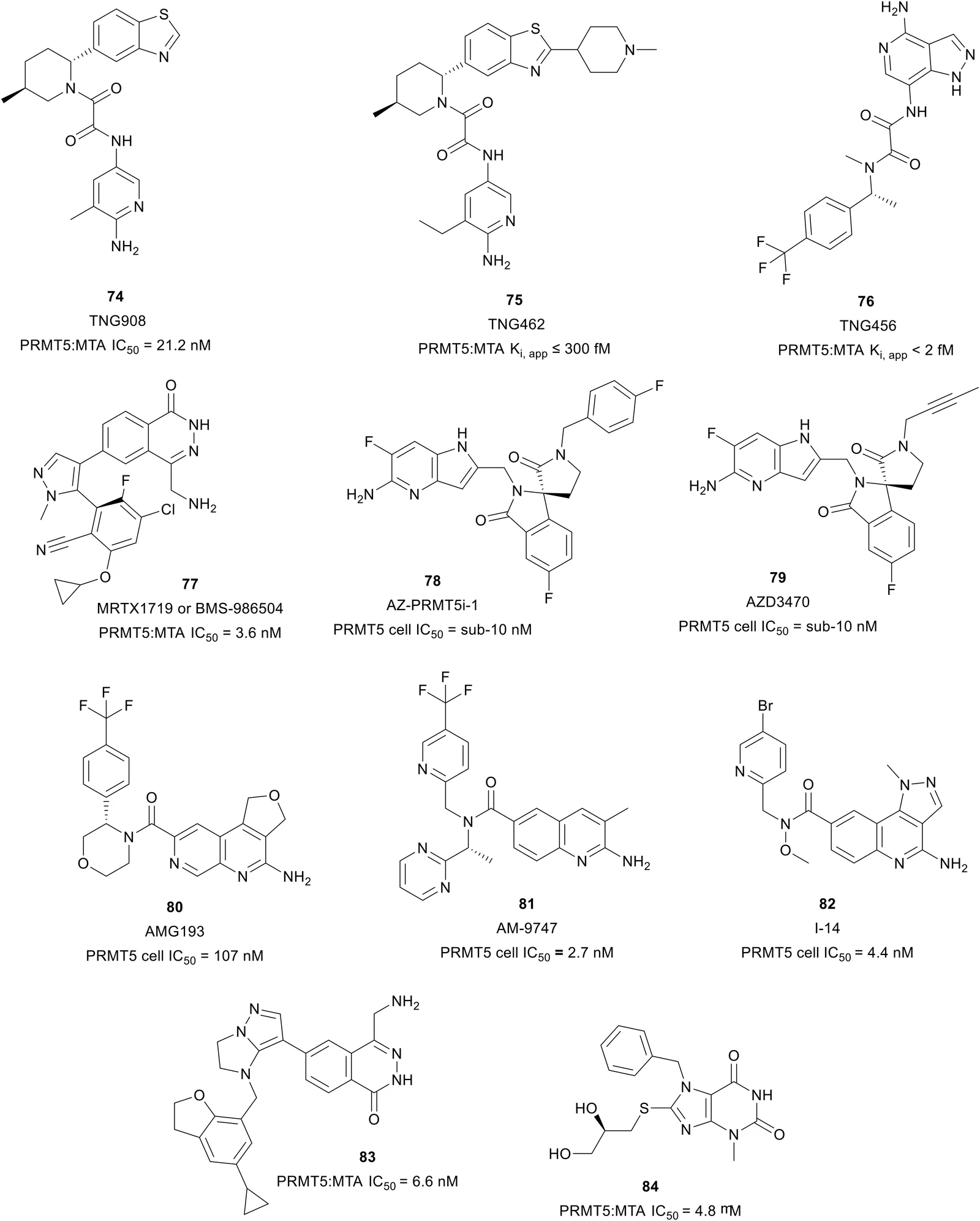
Representative structures of MTA-cooperative PRMT5 inhibitors (74–84). All IC_50_/*K*_i_ values were determined in biochemical assays unless otherwise stated. The compounds are characterized by diverse chemical scaffolds, but all are able to bind and stabilize the PRMT5:MTA complex in MTAP-null cancer cells in order to selectively target PRMT5 in selected cancer cells.

Based on these premises, a large number of MTA-cooperative PRMT5 inhibitors have been developed (Chart 11). From a structural standpoint, these compounds incorporate diverse chemical scaffolds, but all share the unique ability to bind and stabilize the PRMT5:MTA complex in *MTAP*-null cancer cells, thereby selectively inhibiting PRMT5 activity.^147,173^

In 2024, a high-throughput biochemical screening of a 560k Tango Therapeutics compound library, followed by ligand- and structure-based drug design, led to the identification of compound 74 (TNG908). This molecule binds PRMT5 with a *K*_D_ of 1.9 nM and the PRMT5:MTA complex with a 6-fold increase in potency (*K*_D_ = 0.3 nM). Accordingly, it strongly inhibited the PRMT5:MTA complex (IC_50_ = 21.2 nM, *K*_i,app_ = 0.26 nM) compared to PRMT5 alone (IC_50_ = 262 nM, *K*_i,app_ = 3.2 nM). Moreover, the selective inhibition of PRMT5 for MTAP-null cells was confirmed by profiling the compound against a panel of 38 methyltransferases, in which only PRMT5-containing complexes were inhibited more than 90% at 10 μM. Compound 74 showed a favourable ADME-PK profile across preclinical species, including rats, beagle dogs, and cynomolgus monkeys. Further *in vivo* studies highlighted on-target, dose- and concentration-dependent antitumour activity selectively in *MTAP*-null xenografts, suggesting that 74 could induce clinical responses while maintaining a promising therapeutic index.^174^74 entered a phase I/II clinical trial in patients with *MTAP*-null solid tumours (NCT05275478); however, the study was subsequently terminated.^175^ Subtle structural manipulation of 74 led to compound 75 (TNG462), which showed a biochemical potency below the limit of assay detection with an estimated *K*_i_ ≤ 300 fM toward the PRMT5:MTA complex and a 45-fold selectivity over *MTAP* wild-type cells. 75 demonstrated robust efficacy in cell-derived and patient-derived xenograft models with a high prevalence of *MTAP* deletion, including pancreatic, non-small-cell lung, mesothelioma, and bladder cancers.^176^ Compound 75 is currently undergoing phase I/II clinical evaluation (NCT05732831; NCT06922591). Tango Therapeutics developed another potent and selective MTA-cooperative PRMT5 inhibitor, compound 76 (TNG456) which, like compounds 75, exhibited potency below the detection limit of biochemical assays, in the presence (*K*_i,app_ ∼ 30 pM) or absence (*K*_i,app_ < 2 pM) of MTA. The selectivity profile was evaluated against a panel of 40 methyltransferases, including PRMT7 and type I PRMTs, and no significant inhibitory activity against any targets other than PRMT5:MEP50 was detected. The compound showed strong preclinical evidence of brain penetrance, rendering it highly suitable for patients with *MTAP*-null malignancies like gliomas or brain metastases.^177,178^76 entered clinical trials as monotherapy and in combination with abemaciclib in patients with solid tumours harbouring *MTAP* loss (NCT06810544).

In 2022, Mirati Therapeutics reported the identification of compound 77 (MRTX1719 or BMS-986504) through a fragment-based lead discovery approach. The compound binds PRMT5 much more tightly in the presence of MTA than SAM, showing sub-picomolar affinity (*K*_D_ = 0.140 pM) and a markedly slower dissociation rate from the PRMT5:MTA complex (a *k*_off_ half-life of 14 days) compared to the PRMT5:SAM state (*K*_D_ = 9.4 pM with a *k*_off_ half-life of 4.6 days). 77 inhibits the PRMT5:MTA complex with a 70-fold higher potency (IC_50_ = 3.6 nM) compared to PRMT5 alone (IC_50_ = 20.4 nM), acting *via* competition with the peptide substrate. The preference for PRMT5:MTA binding was consistent in a cellular context, efficiently blocking PRMT5-mediated SDMA protein modification and cell viability in *MTAP*-null compared with *MTAP* wild-type HCT116 cancer cells.^179,180^ Overall, the inhibitor exhibited encouraging antitumour activity, including tumour regressions, across different cancer models such as lung, pancreatic, and mesothelioma cell line-derived (CDX) or patient-derived xenografts (PDX).

Compound 77 is currently in a first-in-human, phase I/II clinical trial in patients with solid tumours harbouring *MTAP* gene deletions (NCT05245500). Partial responses observed in patients with advanced cancers demonstrated great therapeutic potential with none of the dose-limiting haematological toxicities often associated with canonical PRMT5 inhibitors.^180^ Other clinical trials are currently recruiting to evaluate the performance of the compound in different *MTAP*-null clinical settings, including: a randomized phase II/III study in combination with pembrolizumab and chemotherapy *versus* placebo in patients with first-line metastatic non-small-cell lung cancer (NCT07063745); a randomized phase II/III study comparing 77 in combination with nab-paclitaxel/gemcitabine *versus* placebo in participants with untreated metastatic pancreatic ductal adenocarcinoma (NCT07076121); and a phase I open-label, multi-centre study to evaluate pharmacokinetics, safety, and tolerability in Japanese and Chinese participants with advanced solid tumours (NCT07077434).

In 2024, AstraZeneca performed an HTS of an in-house collection to identify MTA-cooperative hits, which were further optimized using surface plasmon resonance (SPR) and X-ray crystallography to obtain the lead compound 78 (AZ-PRMT5i-1). It demonstrated sub-nanomolar cellular potency against PRMT5 (sub-10 nM), over 50-fold MTA-dependent cooperativity, and high selectivity against a panel of methyltransferases, including other PRMTs. Cellular assays demonstrated selective growth inhibition in *MTAP*-null cell lines, showing significantly weaker effects on their *MTAP*-proficient counterparts. Moreover, 78 drove significant PRMT5-mediated tumour growth inhibition in preclinical models.^181^ The compound was used as a tool compound to develop a clinical candidate, compound 79 (AZD3470), with high PRMT5 cell potency (sub-10 nM) which entered a phase I/II trial in patients with *MTAP*-null advanced solid or metastatic tumours (NCT06130553).^182^

Recently, Amgen described the clinical-stage MTA-PRMT5 cooperative inhibitor 80 (AMG-193), discovered by exploiting a DNA-encoded library (DEL) approach. The compound exhibited high potency in HCT116 *MTAP*-null cells (IC_50_ = 107 nM) and cellular activity across different types of *MTAP*-null cancer cells, inducing DNA damage, cell cycle arrest, and aberrant alternative mRNA splicing. Strong antitumour activity with no deleterious effects on normal hematopoietic cells was observed in several human cell lines (*e.g.*, NSCLC, pancreatic, renal, ovarian, gallbladder, and oesophageal cancers) and patient-derived xenograft models.^183^ Compound 80 is being tested alone or in combination with chemotherapy or immunotherapy in clinical trials (NCT06333951, NCT06360354, NCT05094336, NCT06593522), or in combination with IDE397, a potent inhibitor of methionine adenosyltransferase 2A (NCT05975073) in patients with advanced solid *MTAP*-deleted cancers.

The DEL approach also led to compound 81 (AM-9747), which is structurally related to 80. It demonstrated high potency (IC_50_ = 9.5 nM) in *MTAP*-null viability assays and 75-fold selectivity over corresponding wild-type *MTAP* lines. Moreover, 81 displayed 248-fold selectivity in inhibiting arginine dimethylation in HCT116 *MTAP*-null cells (IC_50_ = 2.7 nM) compared to wild-type counterparts (IC_50_ = 520 nM). Despite modest oral bioavailability in mice, once-daily dosing for 17 days resulted in dose-dependent tumour growth inhibition, including up to 81% and 20% tumour regression in CDX and PDX models, respectively.^184^ Favourable lead optimization of 81 included the insertion of an *N*-methylpyrazole ring into the quinoline scaffold to enhance affinity for the PRMT5:MTA complex and the introduction of a hydroxamic acid ester fragment on the amide group. The resulting compound 82 (I-14) exhibited excellent inhibitory biochemical activity against the PRMT5:MTA complex (IC_50_ = 4.4 nM), strongly reducing arginine dimethylation levels in HCT116 *MTAP*-null cells (IC_50_ = 0.89 nM) with 1459-fold selectivity over the *MTAP*-WT SDMA assay. It demonstrated potent antitumour efficacy in an MDA-MB-231-based TNBC xenograft model superior to compound 81, with effects directly related to the on-target inhibition of PRMT5. Despite certain aspects requiring deeper investigation, such as its ability to penetrate the blood–brain barrier and its broader applicability beyond TNBC, the compound has emerged as a highly valuable drug candidate.^185,186^

Very recently, other MTA-cooperative PRMT5 inhibitors have been disclosed. A structure-based drug design approach allowed the identification of novel PRMT5:MTA inhibitors characterized by a 2,3-dihydro-1*H* imidazo[1,2-*b*]pyrazole scaffold. Among these, compound 83 emerged as the most promising, demonstrating high potency against PRMT5 in presence of MTA (IC_50_ = 6.6 nM) compared to PRMT5 alone (IC_50_ = 2235 nM) and a good activity in a cellular context, selectively inhibiting PRMT5 in HCT116 *MTAP-null* cells (IC_50_ = 319 nM) compared to MTAP-wild type cells (IC_50_ > 5000 nM).^187^ Exploiting a structure-based virtual screening, compound 84 was developed as PRMT5:MTA inhibitor, demonstrating a strong binding affinity for PRMT5–MTA complex (*K*_D_ of 236 nM) and a higher inhibitory activity of PRMT5 in the presence of MTA (IC_50_ = 4.08 μM). Despite the lower activity compared to other inhibitors, this study disclosed an unreported 3-methylxanthine scaffold as valuable starting point for the development of new inhibitor.^188^

#### PRMT5 inhibitors with mixed inhibitory modalities

**Chart 12:**
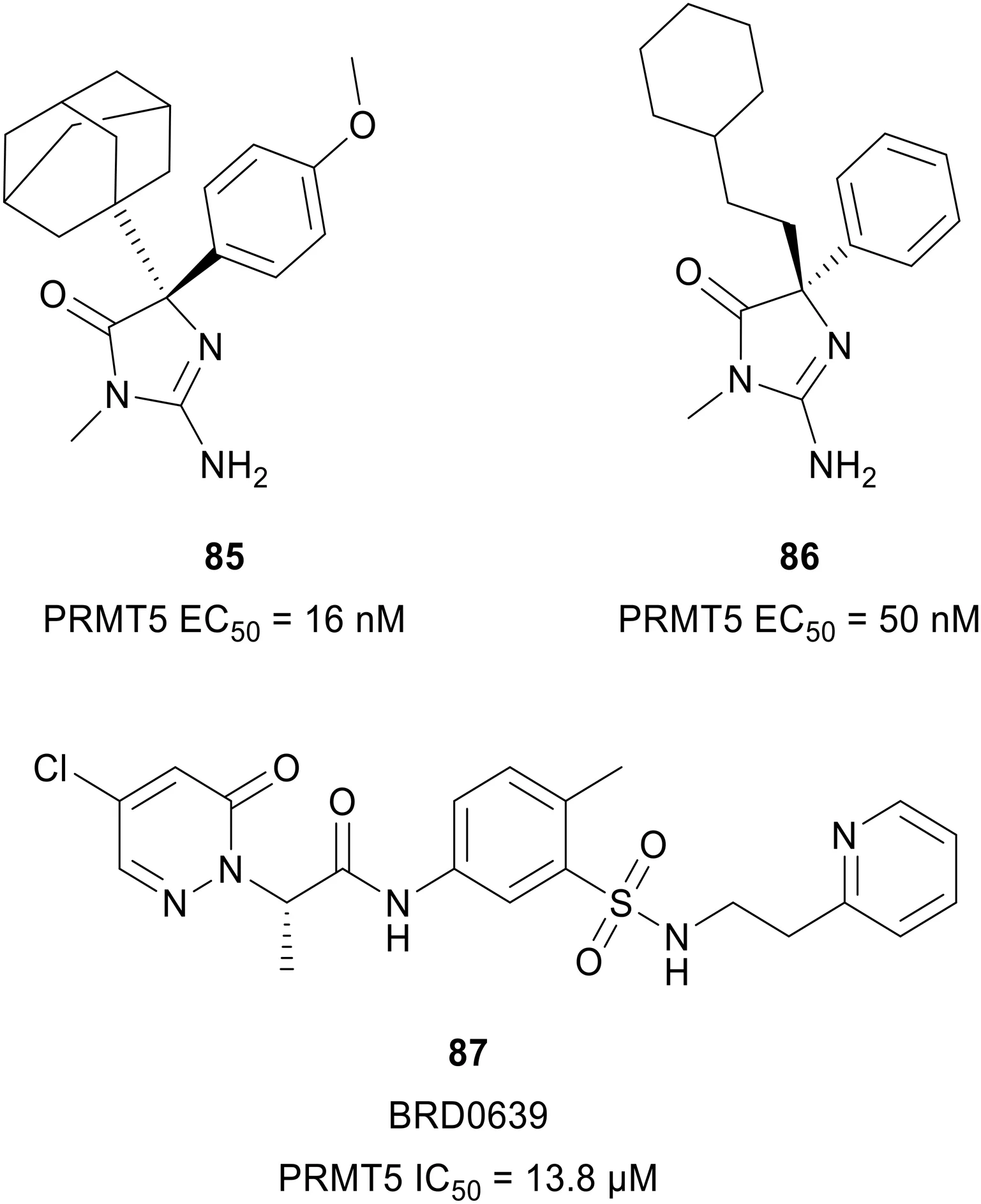
Representative PRMT5 inhibitors with mixed inhibitory modalities. All IC_50_ values were determined in biochemical assays unless otherwise stated. Compound 85, a BACE1 and BACE2 inhibitor, and compound 86 offered structural insights to the development of PRMT5 allosteric inhibitors. Compound 87 represents a first-in-class inhibitor of the interaction between PRMT5 and its substrate adaptor proteins (SAPs).

Besides the PRMT5 inhibitors described so far, other innovative classes of compounds have been discovered (Chart 12).

In 2020, a set of dual substrate- and SAM-competitive inhibitors was reported, offering unique structural insights for developing selective allosteric inhibitors. The co-crystal structure of the PRMT5:MEP50 complex with 85, a known BACE1 and BACE2 inhibitor also identified as a potent PRMT5 binder (EC_50_ = 16 nM), revealed profound structural modifications in PRMT5 conformation. Compound 85 was nearly 200-fold more potent than its corresponding (*S*)-enantiomer, although its selectivity over other PRMT isoforms or methyltransferases was not reported. Upon binding, an 11-amino-acid loop is displaced and shifts to simultaneously occupy portions of both the SAM and substrate-binding sites, stabilizing an inactive enzyme conformation in which large sections of the catalytic machinery are rearranged, thereby preventing the entry of both substrate and SAM. Moreover, 85 induced the opening of a small channel extending from the allosteric site toward the substrate-binding region, providing structural directions to further elaborate the compound. Structural insights of the related compound 86 highlighted its binding in an intermediate loop state between the substrate-bound conformation and the fully shifted state stabilized by 85, indicating that this loop region can adopt multiple distinct conformations depending on the specific allosteric modifier engaged.^189^

In 2021, McKinney and co-workers discovered a first-in-class small-molecule inhibitor targeting the interaction between PRMT5 and its substrate adaptor proteins (SAPs). Beyond PRMT5 itself, specific PRMT5 SAPs, including pICln, RIOK1, and its obligate partner WDR77, are involved in the synthetic lethal phenotypes observed in *MTAP*-null cancers, suggesting an alternative strategy to selectively modulate PRMT5 activity. Different hit-finding strategies were employed to screen large chemical libraries and identify molecules capable of binding the conserved PRMT5-binding motif (PBM) found in SAPs. A chemical series emerged as highly effective in directly disrupting SAP binding *via* the formation of a covalent bond between its halogenated pyridazinone core and the Cys278 residue within PRMT5. Hit-to-lead optimization yielded compound 87 (BRD0639), which inhibited PRMT5 with IC_50_ of 13.8 μM and successfully engaged the target in cells, effectively disrupting the PRMT5-RIOK1 binding with IC_50_ values of 7.5 μM and 16 μM in permeabilized and living cells, respectively. These data are poised to drive the design of next-generation PBM-dependent PRMT5 inhibitors that can selectively impair splicing functions mediated by precise SAP interactions.^190^

### PRMT9

PRMT9 is the second member of the type II PRMT family, though it has been less extensively characterized than PRMT5. Like PRMT7, PRMT9 contains an ancestral amino acid sequence duplication that forms a pseudo-dimeric structure harbouring a second putative, non-catalytic SAM-binding domain. However, unlike other PRMT family members, PRMT9 is uniquely characterized by three N-terminal tetratricopeptide repeat (TPR) motifs. Regarding substrate specificity, PRMT9 differs significantly from PRMT5, and the two type II enzymes do not display functional redundancy. PRMT9 is found *in vivo* within a stable cellular complex with splicing factors SF3B2 (SAP145) and SF3B4 (SAP49), functionally linking it to the regulation of alternative splicing. PRMT9 specifically methylates SF3B2 at Arg108 within a conserved Cys-Phe-Gly-Arg-Lys-Tyr-Leu motif, creating a molecular docking platform recognized by the Tudor domain of the survival of motor neuron (SMN) protein.^4,191^ Recently, a missense PRMT9 mutation (Gly189Arg) was identified in patients presenting with autosomal recessive intellectual disability (ARID). The Gly189Arg substitution abolishes PRMT9 catalytic activity toward SF3B2 Arg508 methylation and consequently triggers PRMT9 ubiquitination and degradation. PRMT9 loss in excitatory neurons resulted in aberrant synapse development and impaired learning and memory, establishing a causal link between PRMT9 loss-of-function and ARID pathology.^192^ Furthermore, analysis of bi-allelic PRMT9 loss-of-function variants in 35 individuals presenting with global developmental delay, intellectual disability, autism, epilepsy, and hypotonia has further implicated PRMT9 deficiency in autosomal-recessive forms of intellectual disability.^193^

PRMT9 has also been linked to various oncological contexts. For example, PRMT9 loss in acute myeloid leukaemia (AML) reduced the arginine methylation of key regulators involved in RNA translation and the DNA damage response, significantly impairing leukaemia stem cell survival. Moreover, PRMT9 inhibition activated the cyclic GMP-AMP synthase (cGAS) pathway, triggering a robust type I IFN response. Thus, targeting PRMT9 could simultaneously eradicate leukaemia stem cells and stimulate an anticancer immune response to enhance therapeutic outcomes.^194^ Conversely, PRMT9 has been reported to promote the invasion and metastasis of hepatocellular carcinoma (HCC), where higher PRMT9 levels correlate with poor overall survival in HCC patients.^195^

### PRMT9 inhibitors

**Chart 13:**
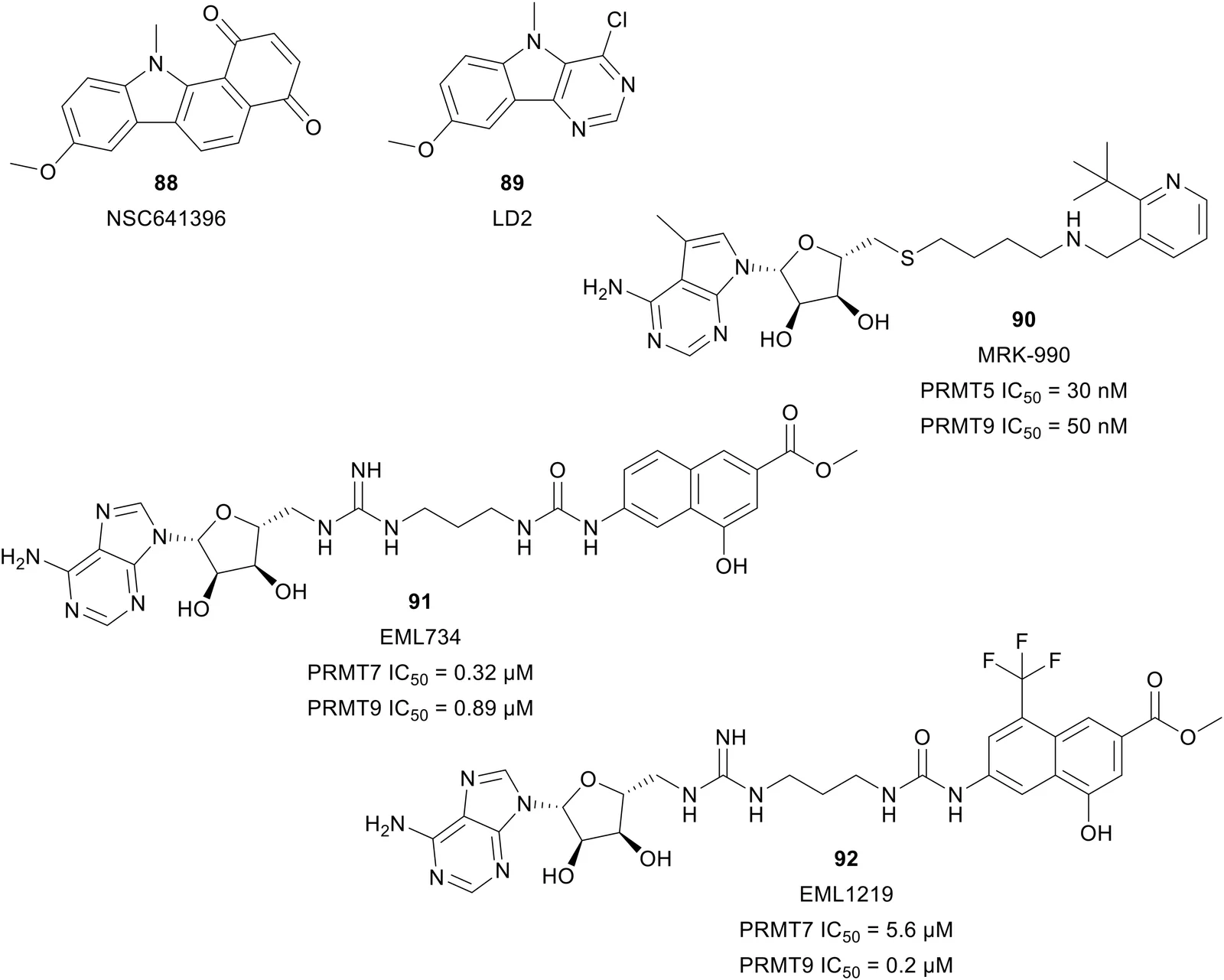
Representative compounds able to interact with PRMT9, including the ribonucleotide reductase inhibitor 88 (NSC641396) identified as PRMT9 binder and its analogue 89 (LD2), the dual PRMT5/PRMT9 inhibitor 90 (MRK-990), the dual PRMT7/9 inhibitor 91 (EML734) and the PRMT9 unselective inhibitor 92 (EML1219). These compounds illustrate the structural evolution of PRMT9-directed chemotypes toward improved affinity, selectivity, and pharmacokinetic properties. All IC_50_ values were determined in biochemical assays unless otherwise stated.

Unlike PRMT5, the development of selective PRMT9 inhibitors has proven exceptionally challenging, and to date, no highly selective single-target small molecules have reached late-stage validation. This timeline is partly due to a historical lack of structural data; a crystal structure of PRMT9 has only recently become available (PDB: 6PDM), which has limited structure-guided optimization campaigns. In 2024, a virtual screening of compounds from the National Cancer Institute (NCI) and ZINC libraries identified compound 88 (NSC641396, Chart 13)—previously reported as a ribonucleotide reductase inhibitor—as a direct PRMT9 binder.^194,196^ Subsequent Tanimoto-based two-dimensional similarity searches led to the identification of compound 89 (LD2, Chart 13), whose direct binding to PRMT9 was confirmed by nuclear magnetic resonance (NMR) and cellular thermal shift assays (CETSA). 89 preferentially targets PRMT9 while largely sparing other PRMT family members. A slight inhibition of PRMT5 was observed at the highest concentration tested (20 μM), and docking studies supported its preferential binding to PRMT9 over PRMT5, PRMT7, and CARM1. The compound preferentially inhibited the viability of cancer cells such as MV4–11, NB4, U937, MA9.6ITD, Molm13 and THP1 with IC_50_ values ranging from 2 to 7 μM.

Notably, it successfully reduced PRMT9 activity in cells, promoting robust anti-AML activity.^194^ Merck Sharp & Dohme (MSD), in collaboration with the Structural Genomics Consortium (SGC), reported the dual PRMT5/PRMT9 chemical probe 90 (MRK-990, Chart 13), which is active against both PRMT9 (IC_50_ = 10 nM) and PRMT5 (IC_50_ = 30 nM), while activity on other isoform is not reported. In an in-cell western assay, the compound inhibited the methylation of SAP145 (PRMT9 substrate) with an IC_50_ of 145 nM and symmetric dimethylarginine marks (PRMT5) with an IC_50_ of 519 nM.^197^

In 2023, a dual PRMT7/9 inhibitor was described alongside the optimization of an effective biochemical assay format to systematically evaluate PRMT9 inhibitor kinetics. Taking advantage of this assay format, a series of compounds characterized by a methyl 4-hydroxy-2-naphthoate core linked to diverse arginine-mimetic functionalities was screened against PRMT9. Compound 91 (EML734, Chart 13) emerged as a PRMT7/9 inhibitor (IC_50_ = 0.89 μM and 0.32 μM, respectively), displaying a good selectivity profile against other PRMTs (IC_50_ = 32.27 μM, 57.19 μM, 13.84 μM, 52.13 μM, 72.77 μM and 8.29 μM over PRMT1, PRMT4, PRMT5, PRMT6 and PRMT8 respectively). Docking studies performed with the crystal structures of PRMT9 and PRMT7 provided further insights into the binding mode of 91. In PRMT9, the adenine ring occupies the SAM binding pocket, while the arginine-mimetic group is involved in direct ionic contacts with the negatively charged side chains of the double-E loop. In addition, the 4-hydroxy-2-naphthoate group is accommodated within a lipophilic pocket, establishing a favourable π–π interaction with the side chain of Trp152. In PRMT7, the adenosine moiety recapitulates similar interactions within the SAM pocket. The main differences are observed for the terminal 4-hydroxy-2-naphthoate group, which in the case of PRMT7 is oriented toward a more solvent-exposed and hydrophilic protein region.

To validate this structural hypothesis, the trifluoromethylated analogue 92 (EML1219, Chart 13) was designed to strengthen the π–π contacts with Trp152 without altering the overall binding conformation. Inhibitory activity against PRMT7 was maintained (IC_50_ = 5.6 μM), while a 4-fold increase in inhibitory activity against PRMT9 was achieved (IC_50_ = 0.2 μM) with a strong binding affinity (*K*_D_ = 188 nM) toward this isoform. However, selectivity over other PRMTs, particularly PRMT5 (IC_50_ = 1 μM) but also PRMT3, PRMT4 and PRMT8 (IC_50_ = 14.9 μM, 1.46 μM, 1.97 μM, respectively) was reduced. Moreover, selectivity against non-SET domain methyltransferases like DOT1L, was also compromised. Both inhibitors were evaluated in MCF-7 and MDA-MB-436 breast cancer cell lines, but no immediate phenotypic cellular activity was detected, as expected given the low cell permeability typically associated with these highly polar scaffolds. On the other hand, label-free quantification (LFQ) mass spectrometry confirmed that 91 is capable of modulating PRMT activity patterns in more complex cellular lysates.^198^

## Type III PRMTs

### PRMT7

PRMT7 is the unique type III arginine methyltransferase, specifically catalysing only the monomethylation of terminal guanidino nitrogens on arginine residues.^199^ Unlike other PRMT family members, the enzyme preferentially targets motifs composed of a pair of arginines separated by a single residue (RXR motif) surrounded by clusters of lysine residues. Methylation occurs primarily on Arg29, Arg31, and Arg33 of histone H2B, as well as on Arg17 and Arg19 of histone H4.^200^ Moreover, PRMT7 also promotes the methylation of non-histone substrates, including mammalian translation initiation factor 2α (eIF2α), HSP70 proteins, DVL3, and G3BP2.^201–203^ PRMT7 has been implicated in regulating cellular responses to DNA damage by modulating the transcription of key DNA repair genes *via* H2AR3 and H4R3 promoter methylation.^204^ Furthermore, PRMT7 promotes the monomethylation of MAVS (mitochondrial antiviral-signalling) protein at its arginine residues, negatively regulating its antiviral signalling cascade *in vitro* and *in vivo*, thereby controlling the innate antiviral immune response.^205^

PRMT7 has also been implicated in cardiac hypertrophy and fibrosis, primarily through modulation of the Wnt/β-catenin signalling pathway^206^ as well as in recessive congenital disorders characterized by obesity, skeletal malformations, and intellectual disability.^207^ In oncological contexts, PRMT7 is overexpressed across different malignant tumours, driving cell proliferation, progression, and metastasis.^208^ Mechanistically, PRMT7 Arg531 automethylation induces an epithelial–mesenchymal transition (EMT) program in breast cancer cells, thereby promoting enhanced migration and invasiveness.^209^ High levels of PRMT7 have also been identified in clear cell renal cell carcinoma tissues, where it acts as an oncogene to promote tumour growth by modulating the β-catenin/c-Myc axis.^210^ Similarly, PRMT7 expression in lung cancer tissues has been shown to play a significant role in promoting NSCLC cell invasion.^211^ Interestingly, PRMT7 inhibition combined with immune checkpoint therapies resulted in robust anti-tumour T-cell immunity and pronounced tumour growth inhibition by increasing immune cell infiltration, offering a valuable therapeutic rationale for sensitizing refractory melanomas to checkpoint blockade.^212^

### PRMT7 inhibitors

**Chart 14:**
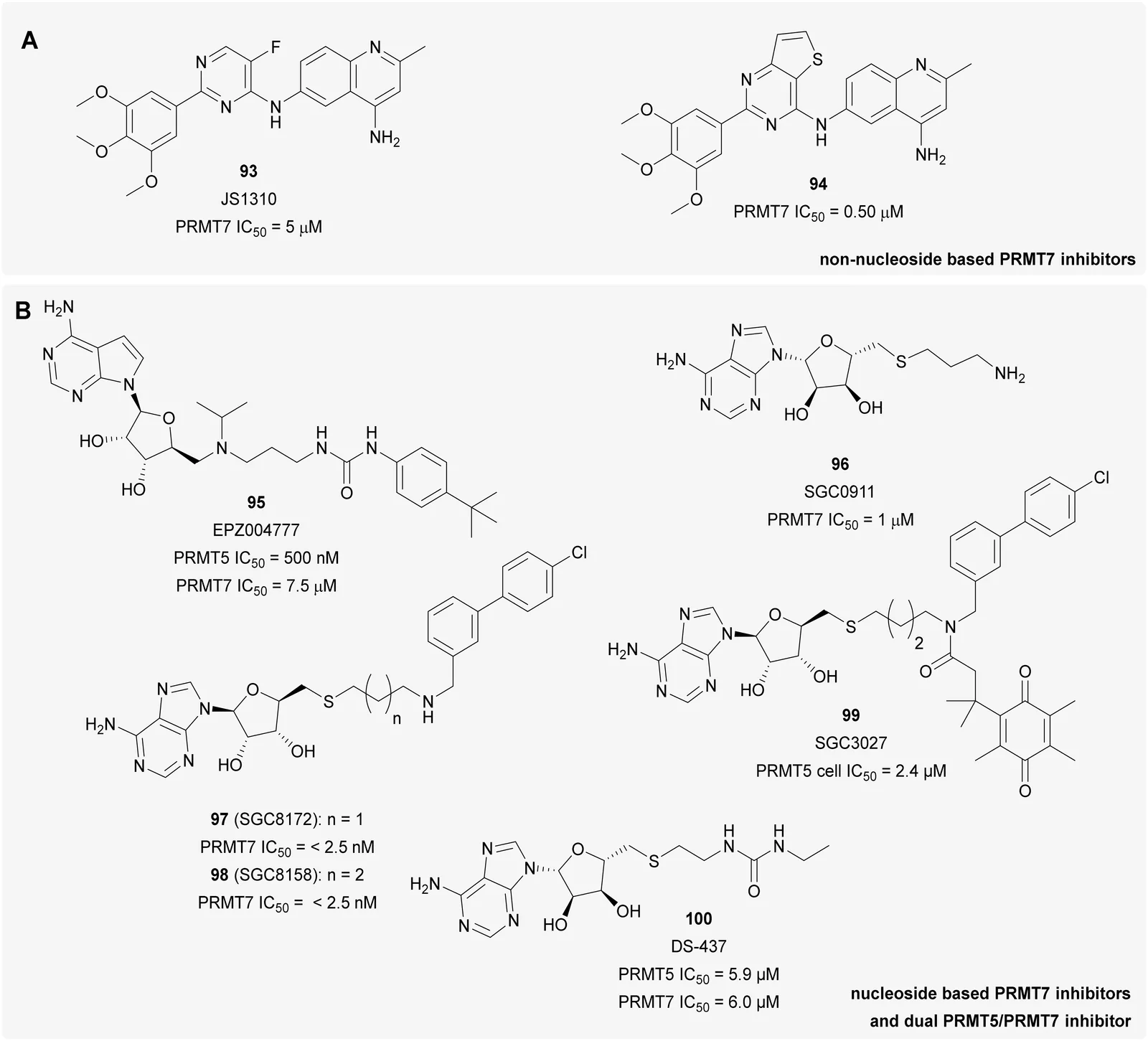
Representative PRMT7 inhibitors classified according to their core structural features. All IC_50_ values were determined in biochemical assays unless otherwise stated. (A) Non-nucleoside PRMT7 inhibitors, including the micromolar PRMT7 inhibitor 93 (JS1310) and the inhibitor 94 (B) representative nucleoside-derived PRMT7 inhibitors, including the pan-methyltransferase inhibitor 95, the SAM-mimetic scaffold 96, subsequently optimized into compounds 97, 98, and the prodrug 99, as well as compound 100, a dual PRMT5/PRMT7 inhibitor. These compounds illustrate the progressive optimization of PRMT7-targeting chemotypes toward improved potency and selectivity.

To date, only a few selective PRMT7 inhibitors have been reported, alongside a small number of dual-target inhibitors (Chart 14).

Non-nucleoside-based PRMT7 inhibitors have been developed, although they generally exhibit low selectivity or modest potency (Chart 14A). In 2022, compound 93 (JS1310) was reported as a selective PRMT7 inhibitor, exhibiting micromolar potency against PRMT7 (IC_50_ = 5 μM) while showing marked selectivity over type I PRMTs (PRMT1, PRMT3, PRMT4, PRMT6, and PRMT8 IC_50_ > 100 μM) and the type II methyltransferase PRMT5 (IC_50_ = 50 μM).

This selectivity profile was confirmed in cellular assays using primary leukemia cells isolated from the spleens of CML model mice, where the compound induced a clear decrease in the symmetric arginine dimethylation of histone H2A (largely catalysed down-stream or in concert with PRMT7 marks) but did not affect the asymmetric dimethylation of histone H4, which is primarily catalysed by PRMT1.^213^ Its scaffold served as the basis for the development of additional non-nucleoside PRMT7 inhibitors. Structure-based docking studies guided the design of a series of analogues through multiple optimization strategies, including replacement of the pyrimidine ring, introduction of different substituents, modification of the amine linker, and structural refinement of the 4-aminoquinoline and trimethoxyphenyl moieties. Among the tested compounds, 94 emerged as the most potent PRMT7 inhibitor (IC_50_ = 0.50 μM) and displayed a 22-fold higher binding affinity for PRMT7 than 93, as indicated by their respective *K*_D_ values of 0.32 and 7.12 μM, respectively. In addition, the compound proved to be highly selective with no appreciable inhibition on PRMT1, PRMT3, PRMT4, PRMT5, PRMT6 and PRMT8 (IC_50_ > 100 μM). Moreover, it showed potent antitumour activity in prostate cancer models by inhibiting cell proliferation, migration, and invasion, inducing cell cycle arrest and apoptosis through PRMT7 inhibition. It also reduced tumour growth in a DU-145 xenograft model with low toxicity.^214^

As with other PRMT family members, nucleoside-based inhibitors (Chart 14B) have been actively pursued, including pan-methyltransferase inhibitors such as SAH^215^ or the SAM analogue 95 (EPZ004777). Originally discovered as a potent inhibitor of the lysine methyltransferase DOT1L, 95 was reported also as a PRMT5 inhibitor (IC_50_ = 500 nM) and was shown to competitively bind also PRMT7 with an IC_50_ of 7.5 μM.^216^

In 2020, a focused screening of a SAM-mimetic small-molecule library against PRMT7 led to the identification of 96 (SGC0911), which showed an IC_50_ of 1 μM. Further optimization yielded compound 97 (SGC8172) with improved biochemical potency (IC_50_ = 2.5 nM) but lacking clear isoform selectivity, showing good potency also against PRMT9 (IC_50_ = 138 nM), PRMT5–MEP50 (IC_50_ = 44 nM) and comparable potency against PRMT4 (IC_50_ = 3.6 nM). Increasing the linker length between the terminal amine moiety and the central adenosine core yielded compound 98 (SGC8158), a highly potent PRMT7 inhibitor (IC_50_ < 2.5 nM) that displayed excellent selectivity over a panel of 35 methyltransferases and kinases, including PRMT1, PRMT3, PRMT6, PRMT8 (IC_50_ = 1–16 μM), as well as PRMT4, PRMT5 PRMT9 (IC_50_ ∼ 120 nM), with a strong binding affinity for PRMT7 (*K*_D_ = 6.4 nM). This molecule acts as a classical SAM-competitive inhibitor that does not compete with the peptide substrate. These data were structurally validated by co-crystallographic studies of full-length *Mus musculus* PRMT7 (MmPRMT7, which shares 93% sequence identity with the human isoform) in complex with the ligand. The ribosyl core of 98 occupies the same structural coordinates as SAH within the SAM pocket, while its biphenylmethylamine auxiliary extends toward the conserved THW loop region.

However, consistent with its highly polar SAM-like structure, 98 was essentially inactive in intact cellular assays due to poor membrane permeability. To overcome this limitation, a trimethyl lock (TML) prodrug strategy was explored, yielding the corresponding prodrug 99 (SGC3027), in which the primary/secondary amine functionality was masked with a quinonebutanoic acid promoiety. Upon entering the cell, 99 undergoes reduction by endogenous cellular reductases followed by rapid intracellular lactonization, successfully releasing the active component 98. In C2C12 cells, 99 efficiently inhibited HSP70 methylation with an IC_50_ of 2.4 μM, a phenotype validated across several common cell lines. Importantly, HSP70 methylation was unaffected by the compound in *Prmt7* knockout MEFs, confirming strict on-target cellular specificity.^217^

Additionally, dual-target PRMT7 inhibitors have been disclosed, including the PRMT7/PRMT9 dual ligand 91 (Chart 13) and compound 100 (DS-437), a dual PRMT5/PRMT7 inhibitor. 100 inhibits the methylation of an H4 peptide by the PRMT5–MEP50 complex with an IC_50_ of 5.9 μM and the methylation of an H2B substrate by PRMT7 with an IC_50_ value of 6.0 μM. Consistently, 100 displayed highly similar binding affinities for both PRMT5:MEP50 and PRMT7, with *K*_D_ values of 25 and 37 μM, respectively. Structural data suggest the compound partially occupies the SAM-binding site, while a local conformational rearrangement of the H4R3 substrate residue accommodates the terminal ethyl-urea moiety without impacting the binding of the peptide substrate backbone.^216^

Overall, these studies establish PRMT7 as a pharmacologically tractable target and demonstrate that high biochemical potency and selectivity can be achieved through both nucleoside- and non-nucleoside-based scaffolds. Nevertheless, achieving an optimal balance among potency, isoform selectivity, cellular permeability, and *in vivo* efficacy remains challenging. Further medicinal chemistry optimization and rigorous validation of cellular target engagement will therefore be essential to translate PRMT7 inhibitors from chemical probes into therapeutically relevant candidates.

## Targeted protein degradation and PRMTs

The significant progress achieved in developing small-molecule inhibitors is occasionally counterbalanced by clinical challenges, including off-target toxicities, emerging resistance mechanisms, and the persistence of traditionally challenging catalytic pockets. Over the past decade, targeted protein degradation (TPD) has emerged as a powerful modality to deactivate selective target proteins by promoting their destruction *via* intrinsic cellular clearance pathways, such as the ubiquitin–proteasome system (UPS) or the lysosome.^218^ The primary class of TPD molecules developed comprises proteolysis-targeting chimeras (PROTACs™). Structurally, these heterobifunctional molecules consist of a ligand that recruits the protein of interest (POI) and a second ligand capable of capturing an E3 ubiquitin ligase—typically the von Hippel–Lindau (VHL) tumour suppressor or cereblon (CRBN)—tethered together *via* an optimized chemical linker. PROTACs induce the proximity of the POI to the E3 ligase machinery, promoting POI polyubiquitination and subsequent degradation by the 26S proteasome.^219^

Other specialized TPD modalities include lysosome-targeting chimeras (LYTACs) and autophagy-targeting chimeras (AUTACs). Furthermore, in addition to bifunctional molecules that directly recruit defined E3 ligases, hydrophobic tagging (HyT) strategies have been disclosed. These molecules consist of a lipophilic small-molecule tag (*e.g.*, adamantane) conjugated to a POI ligand. Through the bulky lipophilic moiety, the HyT destabilizes the target or mimics partially misfolded protein states, triggering molecular chaperone recognition and subsequent proteasomal clearance.^220^

Despite the distinct biochemical mechanisms exploited, these chimeric molecules offer several advantages over traditional pharmacology, including a catalytic mechanism of action and event-driven pharmacology. These attributes can minimize off-target toxicities and circumvent drug resistance profiles associated with classical occupancy-driven inhibition, while offering a viable path to target non-catalytic scaffolding interactions.^218^ In this scenario, diverse degraders have been actively developed across the PRMT family, significantly expanding the epigenetic drug discovery landscape.

### Type-I PRMT degraders

**Chart 15:**
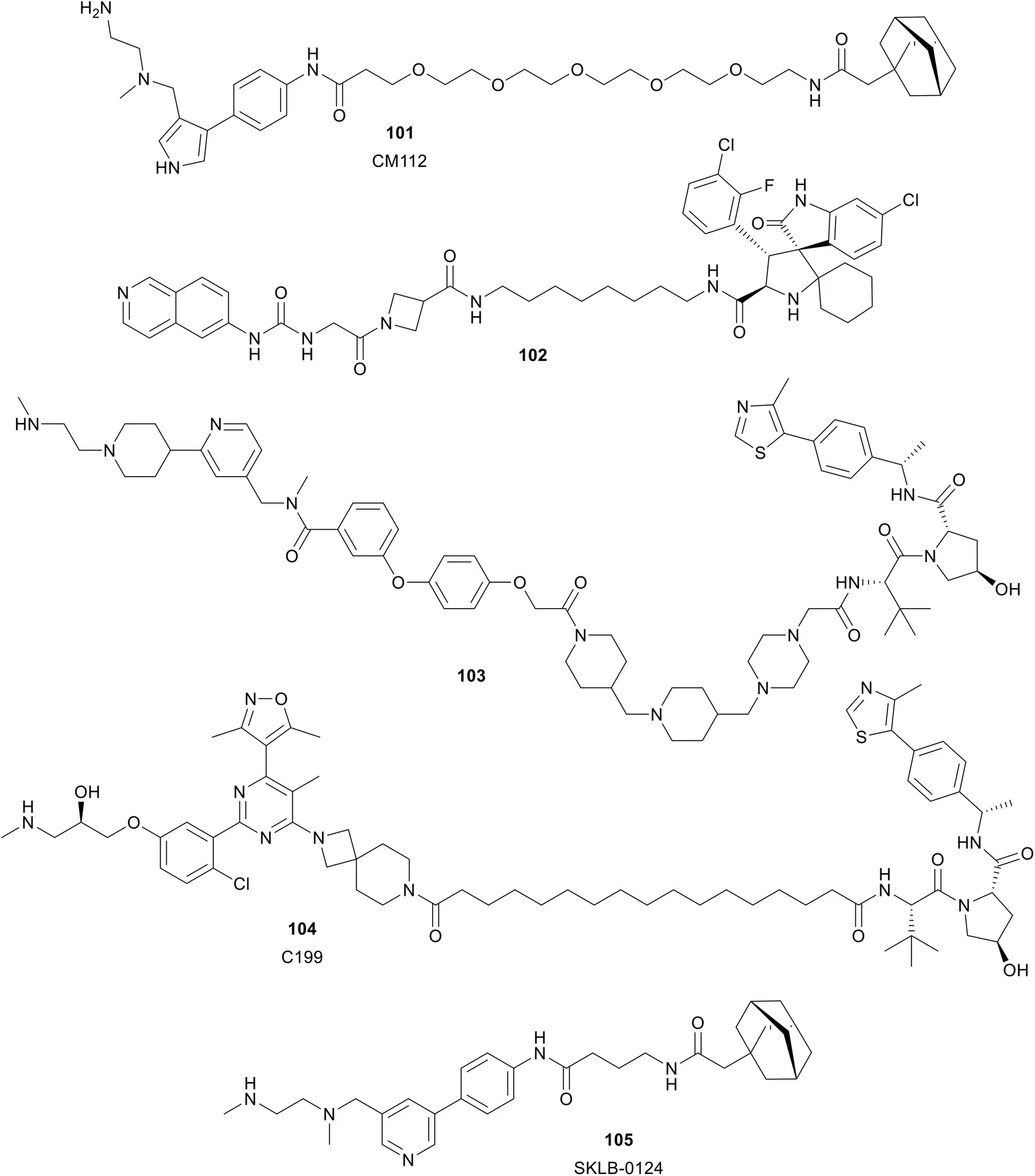
Representative PRMT degraders developed through targeted protein degradation strategies. Compound 101 is a PRMT1 hydrophobic tagging (HyT) degrader, whereas compound 102 is an MDM2 E3 ubiquitin ligase-recruiting PRMT3 PROTAC. Compounds 103 and 104 are VHL E3 ubiquitin ligase-recruiting PRMT4 PROTACs, and compound 105 is a PRMT6 HyT degrader. These molecules highlight the application of both PROTAC™ and hydrophobic tagging approaches for the selective degradation of PRMT family members.

Distinct TPD strategies have been applied to type I PRMT family members, focusing on PRMT1, PRMT3, PRMT4, and PRMT6 (Chart 15).

Regarding PRMT1, two complementary TPD strategies have been explored: PROTACs and HyT degraders. The first systematic optimization campaign was reported by Martin and co-workers, who designed a panel of PRMT1-directed PROTACs by fusing a core pharmacophore derived from 6 to VHL- or CRBN-recruiting elements *via* flexible alkyl or PEG linkers. These heterobifunctional molecules successfully retained cell permeability and potent PRMT1 target inhibition, as demonstrated by reduced global ADMA and concomitantly increased MMA levels, while showing robust CRBN engagement in NanoBRET assays. However, none of the fifteen synthesized PROTAC candidates induced detectable degradation of PRMT1 (or PRMT6) in breast cancer (MCF-7) or pancreatic cancer cell lines at concentrations evaluated up to 10 μM.^221^

In contrast to these initial PROTAC outcomes, a hydrophobic tagging approach provided the first successful proof-of-concept for targeted PRMT1 clearance. Ma *et al.* recently reported compound 101 (CM112), obtained by tethering an adamantane tag to the pan-type I PRMT inhibitor 3*via* a flexible PEG linker. 101 selectively degraded PRMT1 in solid tumour cell lines while sparing PRMT3, PRMT4, and PRMT6. Crucially, 101 retained robust PRMT1 inhibitory activity, successfully reduced cellular ADMA marks, downregulated the established PRMT1 interactor TR3 (whose cellular stability strictly depends on non-catalytic scaffolding functions of PRMT1), and displayed a favourable pharmacokinetic profile *in vivo*.^222^ Together, these studies indicate that PRMT1 is fundamentally degradable but underscore that successful degraders development may require warheads binding to shallower or allosteric surfaces, finely tuned linker geometries, or recruitment of alternative E3 ligase pools to achieve efficient clearance.

Starting from the allosteric inhibitor 31, a first-in-class MDM2 E3 ubiquitin ligase-recruiting PRMT3 degrader (102) was successfully developed. This molecule selectively degraded PRMT3 with a DC_50_ of 2.5 μM and a maximum degradation efficacy *D*_max_ of 90%, effectively reducing asymmetric dimethylation levels. Notably, 102 exhibited superior antiproliferative activity relative to the reference core inhibitor 31 and demonstrated strong synergistic lethality when combined with glycolytic blockade (2-DG) in acute leukemia models.^223^ These findings highlight targeted degradation as an attractive modality to suppress both the catalytic and non-catalytic scaffolding roles of PRMT3, significantly expanding the therapeutic rationale for targeting this isoform.

Targeted protein degradation has also emerged as a compelling strategy to modulate PRMT4/CARM1. This approach is highly appealing given the biological divergence frequently observed between pharmacological PRMT4 catalytic inhibition and complete *Prmt4* genetic knockout in cancer models, which strongly implies critical non-catalytic scaffolding roles that are unaddressed by classical small-molecule inhibitors.^224,225^ The first PRMT4-directed PROTACs were reported in 2023, where the chemical probe 44 was systematically linked to VHL ligands. The lead molecule (103) achieved potent and selective PRMT4 degradation (DC_50_ = 8.1 nM; *D*_max_ = 95%), establishing PRMT4 as a highly tractable target for TPD, though preliminary *in vivo* PK/PD parameters were not disclosed.^226^ More recently, a second-generation bifunctional degrader, 104 (C199), was developed using the high-affinity probe 47 as the PRMT4-binding warhead conjugated to a VHL recruiter. 104 displayed highly selective PRMT4 degradation (DC_50_ = 106 nM; *D*_max_ = 93%) and demonstrated robust target suppression within NCI-H929 mouse xenografts, representing the first successful demonstration of *in vivo* target engagement for a PRMT4-directed degrader.^227^ Together, these studies illustrate that PRMT4 degradation is chemically viable and highlight TPD as a complementary modality to dissect catalytic *versus* non-catalytic disease dependencies.

Similarly, a hydrophobic-tag-derived PRMT6 degrader, 105 (SKLB-0124), was shown to induce selective PRMT6 depletion (EC_50_ = 15.4 μM in HCC827 cells). Notably, 105 significantly outperformed conventional reversible ligands in suppressing methylation-dependent transcriptional outputs across several leukaemia models.^228^ These findings position PRMT6 as highly susceptible to degradation modalities, highlighting the potential of TPD to overcome the limits of traditional occupancy-driven strategies.

### Type II (PRMT5) degraders

**Chart 16:**
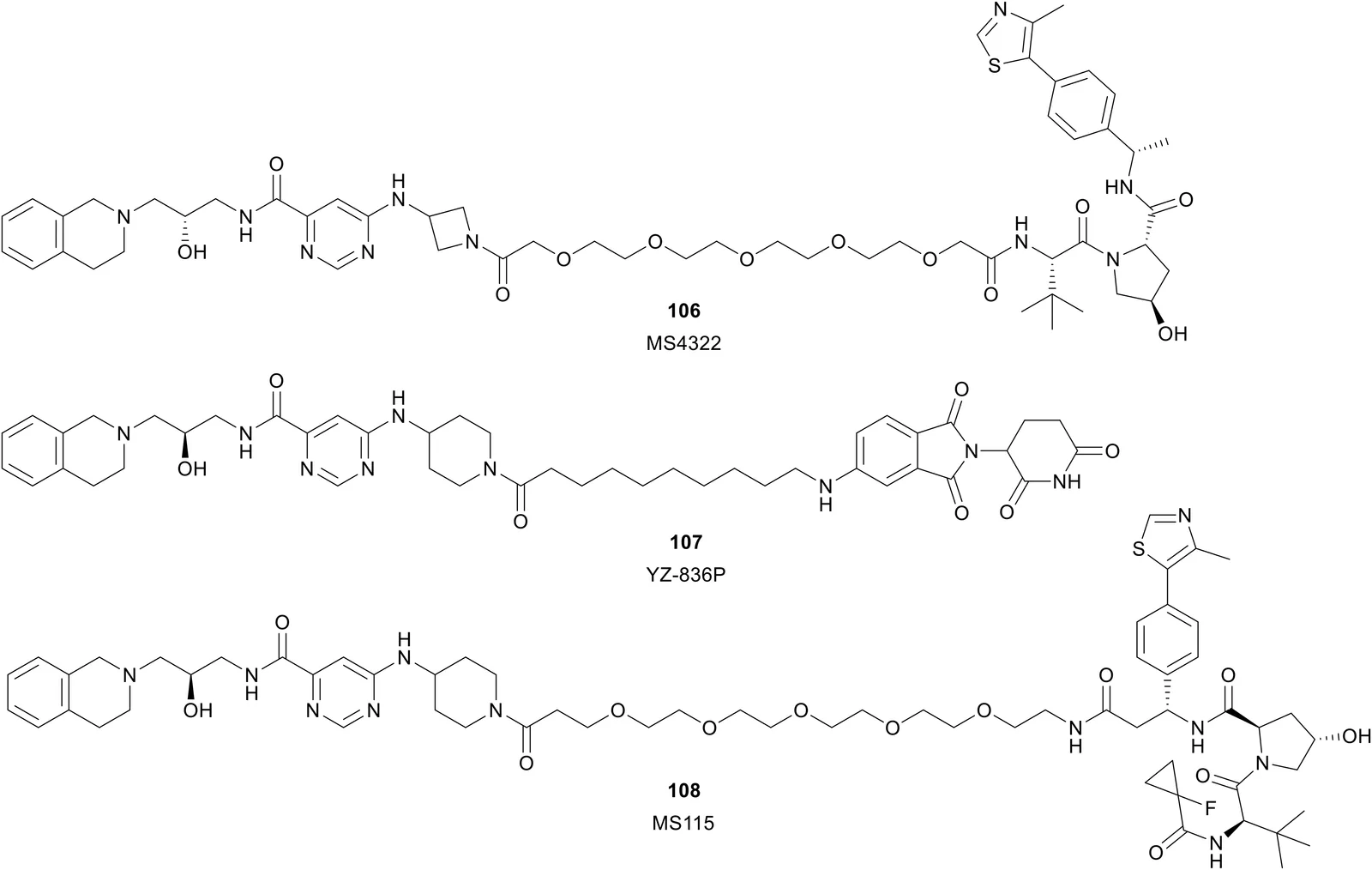
Representative PRMT5-targeting PROTAC™ degraders. Compounds 106 (MS4322), 107 (YZ-38P), and 108 (MS115) were developed by conjugating PRMT5-binding ligands to different E3 ubiquitin ligase recruiters through tailored linker architectures. These degraders illustrate the application of targeted protein degradation strategies to induce selective PRMT5 depletion as an alternative to catalytic inhibition.

In parallel with the development of second-generation MTA-cooperative inhibitors, the PROTAC approach has been extensively exploited as a complementary strategy to target PRMT5, offering the potential to mitigate the dose-limiting haematological toxicities of first-generation inhibitors by leveraging a catalytic mechanism of action (Chart 16). In 2020, Jin and co-workers developed the first-in-class PRMT5 PROTAC, 106 (MS4322), by conjugating the substrate-competitive inhibitor 67 to a VHL E3 ligase ligand. The X-ray co-crystal structure of PRMT5:MEP50 in complex with 67 and SAM helped define the optimal linker attachment site, revealing that the oxetane ring of 67 is highly solvent-exposed and that linker modifications at this vector would minimally impact target binding. To facilitate chemical installation of the spacer, the oxetane ring was replaced with an azetidine core. The resulting PROTAC maintained high biochemical potency (IC_50_ = 18 nM) compared to the parent inhibitor 67 (IC_50_ = 30 nM).

In cellular assays, 106 effectively reduced PRMT5 protein levels in a concentration-dependent manner (DC_50_ value = 1.1 μM) *via* a classic proteasome-dependent pathway in MCF-7 cells, albeit with relatively slow kinetics: significant degradation was observed after 2 days of treatment, reaching maximum target depletion after 6 to 8 days. This depletion translated into a pronounced reduction of global cellular SDMA marks in MCF-7 lines, achieving slightly higher efficiency than 67 alone. Robust PRMT5 degradation was also validated in other distinct cell lineages, including HeLa (cervical), A549 (lung), A172 (glioblastoma), and Jurkat (leukaemia) models, and 106 demonstrated favourable pharmacokinetic properties in mouse models.^229^

In 2024, the chimeric molecule 107 (YZ-836P) was identified as a potent CRBN-recruiting PRMT5 degrader. Structurally, this compound incorporates the advanced inhibitor 69 as the target-binding warhead, with its piperidinyl loop selected as the attachment vector for conjugation to pomalidomide *via* optimized linkers. Cellular studies performed in two distinct TNBC cell lines (HCC1806 and HCC1937) showed that 107 strongly decreased steady-state PRMT5 protein levels in a dose- and time-dependent manner, concomitantly down-regulating KLF5, a critical downstream oncogenic target. Compared to the first-generation degrader 106, PROTAC 107 promoted significantly accelerated kinetics, inducing measurable PRMT5 clearance within 6 hours at 4 μM and reaching optimal degradation within 2 to 3 days. Furthermore, the degrader potently suppressed cell viability and DNA synthesis in these TNBC lines, inducing G1-phase cell cycle arrest, apoptosis, and down-regulation of anti-apoptotic factors. 107 exhibited a highly tolerable safety profile *in vivo*, displaying no significant systemic toxicities while driving pronounced tumour growth inhibition in both patient-derived organoids and breast cancer mouse xenografts.^230^

In 2025, the same consortium that discovered 106 initiated a systematic SAR campaign to engineer highly optimized degraders capable of overcoming the modest degradation potencies observed with early candidates. Two distinct core PRMT5 inhibitors (68 and 69) were evaluated as target-binding warheads, alongside a wide array of rigidified linkers and three distinct VHL ligand variations. From these efforts, PROTAC™ 108 (MS115) emerged as the most effective molecule, inducing rapid and robust PRMT5:MEP50 depletion in a strict time-, concentration-, VHL-, and UPS-dependent manner, outperforming both 106 and 107. Interestingly, 108 inhibits PRMT5 biochemically with a lower initial binding affinity than 106 (IC_50_ of 2.1 μM *versus* 0.69 μM, respectively). This observation is not unexpected in TPD medicinal chemistry, as initial target binding affinities do not always correlate linearly with ultimate degradation clearance, which is driven by cooperative ternary complex formation (POI–PROTAC-E3 ligase). 108 exhibited marked antiproliferative effects across several aggressive breast and prostate cancer cell lines while showing negligible cytotoxicity toward non-transformed cells, and its promising pharmacokinetic profile supports its development for subsequent *in vivo* efficacy evaluations.^231^

## Conclusions

Over the past two decades, research on protein arginine methyltransferases (PRMTs) has led to the identification of a broad spectrum of small-molecule modulators. Early medicinal chemistry efforts largely concentrated on inhibitors targeting either the substrate-binding region or the *S*-adenosyl-l-methionine (SAM) cofactor pocket, exploiting the conserved catalytic framework shared across PRMT family members. These initial studies established the chemical tractability of PRMT enzymes and provided important probe molecules that facilitated mechanistic investigations of PRMT-dependent biological processes. Nevertheless, the high structural similarity within the catalytic core—particularly in the SAM-binding region—has long complicated the development of inhibitors with clear isoform selectivity. More recently, advances in structure-guided design, fragment-based discovery, and computational screening have broadened the chemical diversity of PRMT modulators. Numerous scaffold classes have been reported, encompassing substrate-competitive inhibitors, SAM-competitive ligands, allosteric modulators, and dual-target compounds. For several PRMT isoforms, including PRMT1, PRMT4 (CARM1), PRMT5, and PRMT6, sustained medicinal chemistry optimization has produced compounds displaying nanomolar biochemical potency and good selectivity, together with measurable cellular activity. Beyond their use as chemical probes, these molecules have also laid the groundwork for translational programs aimed at therapeutic development.

Alongside classical occupancy-based inhibition, targeted protein degradation has recently emerged as an alternative strategy to modulate PRMT function. PROTACs and hydrophobic-tag-based degraders illustrate the feasibility of recruiting PRMT proteins to the ubiquitin–proteasome system, thereby promoting their cellular depletion. Although these approaches remain at an early stage, they provide valuable tools to explore both catalytic and scaffolding functions of PRMT enzymes and may ultimately offer improved pharmacological modulation compared with traditional inhibitors.

Despite the progress achieved so far, significant hurdles remain. Achieving robust isoform selectivity within the PRMT family is still difficult due to the conserved nature of the catalytic architecture. In addition, the translation of potent enzymatic inhibitors into molecules with a favourable pharmacokinetic profile and demonstrable *in vivo* efficacy continues to be a primary challenge. Future progress will likely depend on deeper insights into PRMT isoform biology, the identification of context-specific vulnerabilities, and the development of predictive biomarkers to guide therapeutic strategies.

Taken together, the expanding arsenal of PRMT inhibitors and degraders has transformed this enzyme family from an emerging epigenetic target class into a viable platform for chemical biology and drug discovery. Continued integration of structural biology, mechanism-oriented medicinal chemistry, and functional genomics is expected to accelerate the development of next-generation PRMT modulators and to clarify their therapeutic potential across oncology and other disease areas.

## Author contributions

M. V., A. C., B. D. A., C. M., S. C. and G. S. wrote the manuscript. All authors have given approval to the final version of the manuscript.

## Conflicts of interest

There are no conflicts to declare.

## Data Availability

No primary research results, software or code have been included, and no new data were generated or analysed as part of this review.

## References

[cit1] Blanc R. S., Richard S. (2017). Mol. Cell.

[cit2] Clarke T. L., Sanchez-Bailon M. P., Chiang K., Reynolds J. J., Herrero-Ruiz J., Bandeiras T. M., Matias P. M., Maslen S. L., Skehel J. M., Stewart G. S., Davies C. C. (2017). Mol. Cell.

[cit3] Bedford M. T. (2007). J. Cell Sci..

[cit4] Yang Y., Hadjikyriacou A., Xia Z., Gayatri S., Kim D., Zurita-Lopez C., Kelly R., Guo A., Li W., Clarke S. G., Bedford M. T. (2015). Nat. Commun..

[cit5] Lee H. W., Kim S., Paik W. K. (1977). Biochemistry.

[cit6] Tewary S. K., Zheng Y. G., Ho M.-C. (2019). Cell. Mol. Life Sci..

[cit7] Zurita-Lopez C. I., Sandberg T., Kelly R., Clarke S. G. (2012). J. Biol. Chem..

[cit8] Zhang F., Kerbl-Knapp J., Rodriguez Colman M. J., Meinitzer A., Macher T., Vujić N., Fasching S., Jany-Luig E., Korbelius M., Kuentzel K. B., Mack M., Akhmetshina A., Pirchheim A., Paar M., Rinner B., Hörl G., Steyrer E., Stelzl U., Burgering B., Madl T., Rep Cell (2021). Methods.

[cit9] Wolf S. S. (2009). Cell. Mol. Life Sci..

[cit10] Deribe Y. L., Pawson T., Dikic I. (2010). Nat. Struct. Mol. Biol..

[cit11] Wang Y., Bedford M. T. (2023). Biochem. Soc. Trans..

[cit12] Tang J., Frankel A., Cook R. J., Kim S., Paik W. K., Williams K. R., Clarke S., Herschman H. R. (2000). J. Biol. Chem..

[cit13] Lin W. J., Gary J. D., Yang M. C., Clarke S., Herschman H. R. (1996). J. Biol. Chem..

[cit14] Pawlak M. R., Scherer C. A., Chen J., Roshon M. J., Ruley H. E. (2000). Mol. Cell. Biol..

[cit15] Yu Z., Chen T., Hébert J., Li E., Richard S. (2009). Mol. Cell. Biol..

[cit16] Hwang J. W., Cho Y., Bae G.-U., Kim S.-N., Kim Y. K. (2021). Exp. Mol. Med..

[cit17] Xu J., Richard S. (2021). Mol. Cell.

[cit18] Li Z., Wang D., Lu J., Huang B., Wang Y., Dong M., Fan D., Li H., Gao Y., Hou P., Li M., Liu H., Pan Z.-Q., Zheng J., Bai J. (2020). Cell Death Differ..

[cit19] Avasarala S., Van Scoyk M., Karuppusamy Rathinam M. K., Zerayesus S., Zhao X., Zhang W., Pergande M. R., Borgia J. A., DeGregori J., Port J. D., Winn R. A., Bikkavilli R. K. (2015). J. Biol. Chem..

[cit20] Wang Y., Hsu J. M., Kang Y., Wei Y., Lee P. C., Chang S. J., Hsu Y. H., Hsu J. L., Wang H. L., Chang W. C., Li C. W., Liao H. W., Chang S. S., Xia W., Ko H. W., Chou C. K., Fleming J. B., Wang H., Hwang R. F., Chen Y., Qin J., Hung M. C. (2016). Cancer Res..

[cit21] Tang S., Sethunath V., Metaferia N. Y., Nogueira M. F., Gallant D. S., Garner E. R., Lairson L. A., Penney C. M., Li J., Gelbard M. K., Alaiwi S. A., Seo J.-H., Hwang J. H., Strathdee C. A., Baca S. C., AbuHammad S., Zhang X., Doench J. G., Hahn W. C., Takeda D. Y., Freedman M. L., Choi P. S., Viswanathan S. R. (2022). Cell Rep..

[cit22] Brobbey C., Liu L., Yin S., Gan W. (2022). Int. J. Mol. Sci..

[cit23] Fong J. Y., Pignata L., Goy P. A., Kawabata K. C., Lee S. C., Koh C. M., Musiani D., Massignani E., Kotini A. G., Penson A., Wun C. M., Shen Y., Schwarz M., Low D. H., Rialdi A., Ki M., Wollmann H., Mzoughi S., Gay F., Thompson C., Hart T., Barbash O., Luciani G. M., Szewczyk M. M., Wouters B. J., Delwel R., Papapetrou E. P., Barsyte-Lovejoy D., Arrowsmith C. H., Minden M. D., Jin J., Melnick A., Bonaldi T., Abdel-Wahab O., Guccione E. (2019). Cancer Cell.

[cit24] Zheng S., Zeng C., Huang A., Huang F., Meng A., Wu Z., Zhou S. (2022). Biomed. Rep..

[cit25] Mei X., Zeng J., Liu D. F., Zhao Y., Yang H. L., Li Y., Qiu P., Tang M. W. (2021). Ann. Palliat. Med..

[cit26] Cheng D., Yadav N., King R. W., Swanson M. S., Weinstein E. J., Bedford M. T. (2004). J. Biol. Chem..

[cit27] Castellano S., Milite C., Ragno R., Simeoni S., Mai A., Limongelli V., Novellino E., Bauer I., Brosch G., Spannhoff A., Cheng D., Bedford M. T., Sbardella G. (2010). ChemMedChem.

[cit28] Eram M. S., Shen Y., Szewczyk M. M., Wu H., Senisterra G., Li F., Butler K. V., Kaniskan H. Ü., Speed B. A., dela Seña C., Dong A., Zeng H., Schapira M., Brown P. J., Arrowsmith C. H., Barsyte-Lovejoy D., Liu J., Vedadi M., Jin J. (2016). ACS Chem. Biol..

[cit29] Wang C., Jiang H., Jin J., Xie Y., Chen Z., Zhang H., Lian F., Liu Y.-C., Zhang C., Ding H., Chen S., Zhang N., Zhang Y., Jiang H., Chen K., Ye F., Yao Z., Luo C. (2017). J. Med. Chem..

[cit30] Fedoriw A., Rajapurkar S. R., O'Brien S., Gerhart S. V., Mitchell L. H., Adams N. D., Rioux N., Lingaraj T., Ribich S. A., Pappalardi M. B., Shah N., Laraio J., Liu Y., Butticello M., Carpenter C. L., Creasy C., Korenchuk S., McCabe M. T., McHugh C. F., Nagarajan R., Wagner C., Zappacosta F., Annan R., Concha N. O., Thomas R. A., Hart T. K., Smith J. J., Copeland R. A., Moyer M. P., Campbell J., Stickland K., Mills J., Jacques-O'Hagan S., Allain C., Johnston D., Raimondi A., Porter Scott M., Waters N., Swinger K., Boriack-Sjodin A., Riera T., Shapiro G., Chesworth R., Prinjha R. K., Kruger R. G., Barbash O., Mohammad H. P. (2019). Cancer Cell.

[cit31] Sun T., Jin J., Zhou H., Yang J., Xing S., Wang Q., Wang M., Mi Y., Wu H., Wu L., Yao J., Lin J., Miao J., Song Z. (2025). J. Clin. Oncol..

[cit32] Heinke R., Spannhoff A., Meier R., Trojer P., Bauer I., Jung M., Sippl W. (2009). ChemMedChem.

[cit33] Yang H., Ouyang Y., Ma H., Cong H., Zhuang C., Lok W. T., Wang Z., Zhu X., Sun Y., Hong W., Wang H. (2017). Bioorg. Med. Chem. Lett..

[cit34] Wu J., Li D., Wang L. (2024). Eur. J. Med. Chem..

[cit35] Sun Y., Wang Z., Yang H., Zhu X., Wu H., Ma L., Xu F., Hong W., Wang H. (2019). Int. J. Mol. Sci..

[cit36] Wang C., Dong L., Zhao Z., Zhang Z., Sun Y., Li C., Li G., You X., Yang X., Wang H., Hong W. (2022). Front. Chem..

[cit37] Zhao Z., Zhang J., Ren Y., Dong L., Wu H., Hong W., Huang H., Yang X., Pang Z., Wang H. (2023). Bioorg. Med. Chem..

[cit38] Su Y., Lok W. T., Zhang M. M., Gao J. M., He J., Chao N., Yuan H., Ouyang Y., Hong Z., Fan Y., Wang D., Huang Y., Wang H., Yang H. (2025). Eur. J. Med. Chem..

[cit39] Spannhoff A., Heinke R., Bauer I., Trojer P., Metzger E., Gust R., Schüle R., Brosch G., Sippl W., Jung M. (2007). J. Med. Chem..

[cit40] Yan L., Yan C., Qian K., Su H., Kofsky-Wofford S. A., Lee W.-C., Zhao X., Ho M.-C., Ivanov I., Zheng Y. G. (2014). J. Med. Chem..

[cit41] Zhang W. Y., Lu W. C., Jiang H., Lv Z. B., Xie Y. Q., Lian F. L., Liang Z. J., Jiang Y. X., Wang D. J., Luo C., Jin J., Ye F. (2017). Chem. Biol. Drug Des..

[cit42] Yu X. R., Tang Y., Wang W. J., Ji S., Ma S., Zhong L., Zhang C. H., Yang J., Wu X. A., Fu Z. Y., Li L. L., Yang S. Y. (2015). Bioorg. Med. Chem. Lett..

[cit43] Feng Y., Li M., Wang B., Zheng Y. G. (2010). J. Med. Chem..

[cit44] Bissinger E. M., Heinke R., Spannhoff A., Eberlin A., Metzger E., Cura V., Hassenboehler P., Cavarelli J., Schüle R., Bedford M. T., Sippl W., Jung M. (2011). Bioorg. Med. Chem..

[cit45] Xie Y., Zhou R., Lian F., Liu Y., Chen L., Shi Z., Zhang N., Zheng M., Shen B., Jiang H., Liang Z., Luo C. (2014). Org. Biomol. Chem..

[cit46] Zhang J., Ren Y., Teng Y., Wu H., Xue J., Chen L., Song X., Li Y., Zhou Y., Pang Z., Wang H. (2025). Front. Chem..

[cit47] de Lera A. R., Ganesan A. (2016). Clin. Epigenet..

[cit48] Gunnell E. A., Al-Noori A., Muhsen U., Davies C. C., Dowden J., Dreveny I. (2020). Biochem. J..

[cit49] Liu C., Li Y., Liu Z., Cao C., Lin M., Chen X., Yuan M., Fan Y., Gu X., Wang L., Yang F., Ye F., Jin J. (2024). Eur. J. Med. Chem..

[cit50] Tang J., Gary J. D., Clarke S., Herschman H. R. (1998). J. Biol. Chem..

[cit51] Bachand F., Silver P. A. (2004). EMBO J..

[cit52] Swiercz R., Cheng D., Kim D., Bedford M. T. (2007). J. Biol. Chem..

[cit53] Hu Y., Su Y., He Y., Liu W., Xiao B. (2021). Gene.

[cit54] Zhang X., Wang K., Feng X., Wang J., Chu Y., Jia C., He Q., Chen C. (2021). Cell Death Dis..

[cit55] Siarheyeva A., Senisterra G., Allali-Hassani A., Dong A., Dobrovetsky E., Wasney G. A., Chau I., Marcellus R., Hajian T., Liu F., Korboukh I., Smil D., Bolshan Y., Min J., Wu H., Zeng H., Loppnau P., Poda G., Griffin C., Aman A., Brown P. J., Jin J., Al-Awar R., Arrowsmith C. H., Schapira M., Vedadi M. (2012). Structure.

[cit56] Huang L., Wang Z., Narayanan N., Yang Y. (2018). Nucleic Acids Res..

[cit57] Zhi R., Wu K., Zhang J., Liu H., Niu C., Li S., Fu L. (2023). Cancer Sci..

[cit58] Lei Y., Han P., Chen Y., Wang H., Wang S., Wang M., Liu J., Yan W., Tian D., Liu M. (2022). Clin. Transl. Med..

[cit59] Kim D. I., Park M. J., Lim S. K., Park J. I., Yoon K. C., Han H. J., Gustafsson J., Lim J. H., Park S. H. (2015). Diabetes.

[cit60] Siarheyeva A., Senisterra G., Allali-Hassani A., Dong A., Dobrovetsky E., Wasney G. A., Chau I., Marcellus R., Hajian T., Liu F., Korboukh I., Smil D., Bolshan Y., Min J., Wu H., Zeng H., Loppnau P., Poda G., Griffin C., Aman A., Brown Peter J., Jin J., Al-awar R., Arrowsmith C. H., Schapira M., Vedadi M. (2012). Structure.

[cit61] Liu F., Li F., Ma A., Dobrovetsky E., Dong A., Gao C., Korboukh I., Liu J., Smil D., Brown P. J., Frye S. V., Arrowsmith C. H., Schapira M., Vedadi M., Jin J. (2013). J. Med. Chem..

[cit62] Kaniskan H. Ü., Szewczyk M. M., Yu Z., Eram M. S., Yang X., Schmidt K., Luo X., Dai M., He F., Zang I., Lin Y., Kennedy S., Li F., Dobrovetsky E., Dong A., Smil D., Min S.-J., Landon M., Lin-Jones J., Huang X.-P., Roth B. L., Schapira M., Atadja P., Barsyte-Lovejoy D., Arrowsmith C. H., Brown P. J., Zhao K., Jin J., Vedadi M. (2015). Angew. Chem., Int. Ed..

[cit63] Xie Z., Tian Y., Guo X., Xie N. (2024). Cell. Oncol..

[cit64] Chen D., Ma H., Hong H., Koh S. S., Huang S.-M., Schurter B. T., Aswad D. W., Stallcup M. R. (1999). Science.

[cit65] Dashti P., Lewallen E. A., Gordon J. A. R., Montecino M. A., van Leeuwen J., Stein G. S., van der Eerden B. C. J., Davie J. R., van Wijnen A. J. (2023). Bone Rep..

[cit66] Yadav N., Lee J., Kim J., Shen J., Hu M. C.-T., Aldaz C. M., Bedford M. T. (2003). Proc. Natl. Acad. Sci. U. S. A..

[cit67] Cheng D., Vemulapalli V., Lu Y., Shen J., Aoyagi S., Fry C. J., Yang Y., Foulds C. E., Stossi F., Treviño L. S., Mancini M. A., O'Malley B. W., Walker C. L., Boyer T. G., Bedford M. T. (2018). Life Sci. Alliance.

[cit68] Liu L., Lin B., Yin S., Ball L. E., Delaney J. R., Long D. T., Gan W. (2022). Sci. Adv..

[cit69] Quintero C. M., Laursen K. B., Mongan N. P., Luo M., Gudas L. J. (2018). J. Mol. Biol..

[cit70] Lee Y. H., Coonrod S. A., Kraus W. L., Jelinek M. A., Stallcup M. R. (2005). Proc. Natl. Acad. Sci. U. S. A..

[cit71] Vu L. P., Perna F., Wang L., Voza F., Figueroa M. E., Tempst P., Erdjument-Bromage H., Gao R., Chen S., Paietta E., Deblasio T., Melnick A., Liu Y., Zhao X., Nimer S. D. (2013). Cell Rep..

[cit72] Kawabe Y., Wang Y. X., McKinnell I. W., Bedford M. T., Rudnicki M. A. (2012). Cell Stem Cell.

[cit73] Ito T., Yadav N., Lee J., Furumatsu T., Yamashita S., Yoshida K., Taniguchi N., Hashimoto M., Tsuchiya M., Ozaki T., Lotz M., Bedford M. T., Asahara H. (2009). BMC Dev. Biol..

[cit74] Santos M., Hwang J. W., Bedford M. T. (2023). J. Biol. Chem..

[cit75] Suresh S., Huard S., Dubois T. (2021). Trends Cell Biol..

[cit76] Feoli A., Iannelli G., Cipriano A., Milite C., Shen L., Wang Z., Hadjikyriacou A., Lowe T. L., Safaeipour C., Viviano M., Sarno G., Morretta E., Monti M. C., Yang Y., Clarke S. G., Cosconati S., Castellano S., Sbardella G. (2023). J. Med. Chem..

[cit77] Iannelli G., Milite C., Marechal N., Cura V., Bonnefond L., Troffer-Charlier N., Feoli A., Rescigno D., Wang Y., Cipriano A., Viviano M., Bedford M. T., Cavarelli J., Castellano S., Sbardella G. (2022). J. Med. Chem..

[cit78] van Haren M. J., Marechal N., Troffer-Charlier N., Cianciulli A., Sbardella G., Cavarelli J., Martin N. I. (2017). Proc. Natl. Acad. Sci. U. S. A..

[cit79] Cheng D., Valente S., Castellano S., Sbardella G., Di Santo R., Costi R., Bedford M. T., Mai A. (2011). J. Med. Chem..

[cit80] Castellano S., Spannhoff A., Milite C., Dal Piaz F., Cheng D., Tosco A., Viviano M., Yamani A., Cianciulli A., Sala M., Cura V., Cavarelli J., Novellino E., Mai A., Bedford M. T., Sbardella G. (2012). J. Med. Chem..

[cit81] Ragno R., Simeoni S., Castellano S., Vicidomini C., Mai A., Caroli A., Tramontano A., Bonaccini C., Trojer P., Bauer I., Brosch G., Sbardella G. (2007). J. Med. Chem..

[cit82] Zeng H., Wu J., Bedford M. T., Sbardella G., Hoffmann F. M., Bi K., Xu W. (2013). ChemBioChem.

[cit83] Copeland R. A., Olhava E. J., Scott M. P. (2010). Curr. Opin. Chem. Biol..

[cit84] Iyamu I. D., Al-Hamashi A. A., Huang R. (2021). Biomolecules.

[cit85] Cai X.-C., Zhang T., Kim E.-j., Jiang M., Wang K., Wang J., Chen S., Zhang N., Wu H., Li F., dela Seña C. C., Zeng H., Vivcharuk V., Niu X., Zheng W., Lee J. P., Chen Y., Barsyte D., Szewczyk M., Hajian T., Ibáñez G., Dong A., Dombrovski L., Zhang Z., Deng H., Min J., Arrowsmith C. H., Mazutis L., Shi L., Vedadi M., Brown P. J., Xiang J., Qin L.-X., Xu W., Luo M. (2019). eLife.

[cit86] Deng Y., Kim E. J., Song X., Kulkarni A. S., Zhu R. X., Wang Y., Bush M., Dong A., Noinaj N., Min J., Xu W., Huang R. (2024). J. Med. Chem..

[cit87] Huynh T., Chen Z., Pang S., Geng J., Bandiera T., Bindi S., Vianello P., Roletto F., Thieffine S., Galvani A., Vaccaro W., Poss M. A., Trainor G. L., Lorenzi M. V., Gottardis M., Jayaraman L., Purandare A. V. (2009). Bioorg. Med. Chem. Lett..

[cit88] Sack J., Thieffine S., Bandiera T., Fasolini M., Duke G. J., Jayaraman L., Kish K. F., Klei H. E., Purandare A., Rosettani P., Troiani S., Xie D., Bertrand J. A. (2011). Biochem. J..

[cit89] Milite C., Sarno G., Pacilio I., Cianciulli A., Viviano M., Iannelli G., Gazzillo E., Feoli A., Cipriano A., Giovanna Chini M., Castellano S., Bifulco G., Sbardella G. (2024). ChemMedChem.

[cit90] Li X., Zhang L., Xu J., Liu C., Zhang X., Abdelmoneim A. A., Zhang Q., Ke J., Zhang Y., Wang L., Yang F., Luo C., Jin J., Ye F. (2022). J. Chem. Inf. Model..

[cit91] Wan H., Huynh T., Pang S., Geng J., Vaccaro W., Poss M. A., Trainor G. L., Lorenzi M. V., Gottardis M., Jayaraman L., Purandare A. V. (2009). Bioorg. Med. Chem. Lett..

[cit92] Kaniskan H. Ü., Eram M. S., Liu J., Smil D., Martini M. L., Shen Y., Santhakumar V., Brown P. J., Arrowsmith C. H., Vedadi M., Jin J. (2016). MedChemComm.

[cit93] Nakayama K., Szewczyk M. M., dela Sena C., Wu H., Dong A., Zeng H., Li F., Ferreira de Freitas R., Eram M. S., Schapira M., Baba Y., Kunitomo M., Cary D. R., Tawada M., Ohashi A., Imaeda Y., Saikatendu K. S., Grimshaw C. E., Vedadi M., Arrowsmith C. H., Barsyte-Lovejoy D., Kiba A., Tomita D., Brown P. J. (2018). Oncotarget.

[cit94] Zhang Z., Guo Z., Xu X., Cao D., Yang H., Li Y., Shi Q., Du Z., Guo X., Wang X., Chen D., Zhang Y., Chen L., Zhou K., Li J., Geng M., Huang X., Xiong B. (2021). J. Med. Chem..

[cit95] Guo Z., Zhang Z., Yang H., Cao D., Xu X., Zheng X., Chen D., Wang Q., Li Y., Li J., Du Z., Wang X., Chen L., Ding J., Shen J., Geng M., Huang X., Xiong B. (2019). J. Med. Chem..

[cit96] Drew A. E., Moradei O., Jacques S. L., Rioux N., Boriack-Sjodin A. P., Allain C., Scott M. P., Jin L., Raimondi A., Handler J. L., Ott H. M., Kruger R. G., McCabe M. T., Sneeringer C., Riera T., Shapiro G., Waters N. J., Mitchell L. H., Duncan K. W., Ribich S. A. (2017). Sci. Rep..

[cit97] Huang J., Qiao B., Yuan Y., Xie Y., Xia X., Li F., Wang L. (2025). J. Cell. Mol. Med..

[cit98] Peng B.-l., Ran T., Chen X., Ding J.-c., Wang Z.-r., Li W.-j., Liu W. (2024). J. Med. Chem..

[cit99] Frankel A., Yadav N., Lee J., Branscombe T. L., Clarke S., Bedford M. T. (2002). J. Biol. Chem..

[cit100] Gupta S., Kadumuri R. V., Singh A. K., Chavali S., Dhayalan A. (2021). Life.

[cit101] El-Andaloussi N., Valovka T., Toueille M., Steinacher R., Focke F., Gehrig P., Covic M., Hassa P. O., Schär P., Hübscher U., Hottiger M. O. (2006). Mol. Cell.

[cit102] Neault M., Mallette F. A., Vogel G., Michaud-Levesque J., Richard S. (2012). Nucleic Acids Res..

[cit103] Bao J., Di Lorenzo A., Lin K., Lu Y., Zhong Y., Sebastian M. M., Muller W. J., Yang Y., Bedford M. T. (2019). Cancer Res..

[cit104] Stein C., Riedl S., Rüthnick D., Nötzold R. R., Bauer U.-M. (2012). Nucleic Acids Res..

[cit105] Veland N., Hardikar S., Zhong Y., Gayatri S., Dan J., Strahl B. D., Rothbart S. B., Bedford M. T., Chen T. (2017). Cell Rep..

[cit106] Okuno K., Akiyama Y., Shimada S., Nakagawa M., Tanioka T., Inokuchi M., Yamaoka S., Kojima K., Tanaka S. (2018). Carcinogenesis.

[cit107] Sun M., Li L., Niu Y., Wang Y., Yan Q., Xie F., Qiao Y., Song J., Sun H., Li Z., Lai S., Chang H., Zhang H., Wang J., Yang C., Zhao H., Tan J., Li Y., Liu S., Lu B., Liu M., Kong G., Zhao Y., Zhang C., Lin S. H., Luo C., Zhang S., Shan C. (2023). Acta Pharm. Sin. B.

[cit108] Kim N. H., Kim S. N., Seo D. W., Han J. W., Kim Y. K. (2013). Biochem. Biophys. Res. Commun..

[cit109] Herglotz J., Kuvardina O. N., Kolodziej S., Kumar A., Hussong H., Grez M., Lausen J. (2013). Oncogene.

[cit110] Mitchell L. H., Drew A. E., Ribich S. A., Rioux N., Swinger K. K., Jacques S. L., Lingaraj T., Boriack-Sjodin P. A., Waters N. J., Wigle T. J., Moradei O., Jin L., Riera T., Porter-Scott M., Moyer M. P., Smith J. J., Chesworth R., Copeland R. A. (2015). ACS Med. Chem. Lett..

[cit111] Shen Y., Szewczyk M. M., Eram M. S., Smil D., Kaniskan H. Ü., Ferreira de Freitas R., Senisterra G., Li F., Schapira M., Brown P. J., Arrowsmith C. H., Barsyte-Lovejoy D., Liu J., Vedadi M., Jin J. (2016). J. Med. Chem..

[cit112] Shen Y., Li F., Szewczyk M. M., Halabelian L., Park K.-s., Chau I., Dong A., Zeng H., Chen H., Meng F., Barsyte-Lovejoy D., Arrowsmith C. H., Brown P. J., Liu J., Vedadi M., Jin J. (2020). J. Med. Chem..

[cit113] Shen Y., Li F., Szewczyk M. M., Halabelian L., Chau I., Eram M. S., Dela Seña C., Park K.-S., Meng F., Chen H., Zeng H., Dong A., Wu H., Trush V. V., McLeod D., Zepeda-Velázquez C. A., Campbell R. M., Mader M. M., Watson B. M., Jin J. (2021). J. Med. Chem..

[cit114] Zhang Q., Cao J., Zhang Y., Bi Z., Feng Q., Yu L., Li L. (2023). Eur. J. Med. Chem..

[cit115] Lee J., Sayegh J., Daniel J., Clarke S., Bedford M. T. (2005). J. Biol. Chem..

[cit116] Kim J.-D., Park K.-E., Ishida J., Kako K., Hamada J., Kani S., Takeuchi M., Namiki K., Fukui H., Fukuhara S., Hibi M., Kobayashi M., Kanaho Y., Kasuya Y., Mochizuki N., Fukamizu A. (2015). Sci. Adv..

[cit117] Lo L. H., Dong R., Lyu Q., Lai K. O. (2020). Cell Rep..

[cit118] Dong R., Li X., Lai K.-O. (2021). Life.

[cit119] Solari C., Echegaray C. V., Luzzani C., Cosentino M. S., Waisman A., Petrone M. V., Francia M., Sassone A., Canizo J., Sevlever G., Barañao L., Miriuka S., Guberman A. (2016). Biochem. Biophys. Res. Commun..

[cit120] Jeong H. C., Park S. J., Choi J. J., Go Y. H., Hong S. K., Kwon O. S., Shin J. G., Kim R. K., Lee M. O., Lee S. J., Shin H. D., Moon S. H., Cha H. J. (2017). Stem Cells.

[cit121] Lin H., Wang B., Yu J., Wang J., Li Q., Cao B. (2018). J. Cancer.

[cit122] Hernandez S. J., Dolivo D. M., Dominko T. (2017). Oncol. Lett..

[cit123] Zheng K., Zhang Y., Zhang C., Ye W., Ye C., Tan X., Xiong Y. (2022). ACS Chem. Neurosci..

[cit124] Bedford M. T., Clarke S. G. (2009). Mol. Cell.

[cit125] Antonysamy S., Bonday Z., Campbell R. M., Doyle B., Druzina Z., Gheyi T., Han B., Jungheim L. N., Qian Y., Rauch C., Russell M., Sauder J. M., Wasserman S. R., Weichert K., Willard F. S., Zhang A., Emtage S. (2012). Proc. Natl. Acad. Sci. U. S. A..

[cit126] Musiani D., Bok J., Massignani E., Wu L., Tabaglio T., Ippolito M. R., Cuomo A., Ozbek U., Zorgati H., Ghoshdastider U., Robinson R. C., Guccione E., Bonaldi T. (2019). Sci. Signal..

[cit127] Zhao Q., Rank G., Tan Y. T., Li H., Moritz R. L., Simpson R. J., Cerruti L., Curtis D. J., Patel D. J., Allis C. D., Cunningham J. M., Jane S. M. (2009). Nat. Struct. Mol. Biol..

[cit128] Pal S., Vishwanath S. N., Erdjument-Bromage H., Tempst P., Sif S. (2004). Mol. Cell. Biol..

[cit129] Chiang K., Zielinska A. E., Shaaban A. M., Sanchez-Bailon M. P., Jarrold J., Clarke T. L., Zhang J., Francis A., Jones L. J., Smith S., Barbash O., Guccione E., Farnie G., Smalley M. J., Davies C. C. (2017). Cell Rep..

[cit130] Gonsalvez G. B., Tian L., Ospina J. K., Boisvert F. M., Lamond A. I., Matera A. G. (2007). J. Cell Biol..

[cit131] Ren J., Wang Y., Liang Y., Zhang Y., Bao S., Xu Z. (2010). J. Biol. Chem..

[cit132] Barczak W., Jin L., Carr S. M., Munro S., Ward S., Kanapin A., Samsonova A., La Thangue N. B. (2020). Cell Death Dis..

[cit133] Wei T. Y., Juan C. C., Hisa J. Y., Su L. J., Lee Y. C., Chou H. Y., Chen J. M., Wu Y. C., Chiu S. C., Hsu C. P., Liu K. L., Yu C. T. (2012). Cancer Sci..

[cit134] Zheng S., Moehlenbrink J., Lu Y. C., Zalmas L. P., Sagum C. A., Carr S., McGouran J. F., Alexander L., Fedorov O., Munro S., Kessler B., Bedford M. T., Yu Q., La Thangue N. B. (2013). Mol. Cell.

[cit135] Owens J. L., Beketova E., Liu S., Tinsley S. L., Asberry A. M., Deng X., Huang J., Li C., Wan J., Hu C. D. (2020). iScience.

[cit136] Gullà A., Hideshima T., Bianchi G., Fulciniti M., Kemal Samur M., Qi J., Tai Y. T., Harada T., Morelli E., Amodio N., Carrasco R., Tagliaferri P., Munshi N. C., Tassone P., Anderson K. C. (2018). Leukemia.

[cit137] Abe Y., Sano T., Otsuka N., Ogawa M., Tanaka N. (2024). Commun. Biol..

[cit138] Zhu F., Guo H., Bates P. D., Zhang S., Zhang H., Nomie K. J., Li Y., Lu L., Seibold K. R., Wang F., Rumball I., Cameron H., Hoang N. M., Yang D. T., Xu W., Zhang L., Wang M., Capitini C. M., Rui L. (2019). Leukemia.

[cit139] Lin C.-C., Chang T.-C., Wang Y., Guo L., Gao Y., Bikorimana E., Lemoff A., Fang Y. V., Zhang H., Zhang Y., Ye D., Soria-Bretones I., Servetto A., Lee K.-m., Luo X., Otto J. J., Akamatsu H., Napolitano F., Mani R., Cescon D. W., Xu L., Xie Y., Mendell J. T., Hanker A. B., Arteaga C. L. (2024). Nat. Commun..

[cit140] Gaffney D. S., Thuring J. W., Hulpia F., Vesely E., Retzbach E., Etwebi Z., Jiang Z., Francis A., Mattson B., Wong V., Silva M., Hag G. t., Overmeer R., Verissimo C., Boj S. F., Nicolai J., Hixon M., Chu G., DiSandro A., Clancy K., Liddane A., Bachman K., Pocalyko D. (2025). Cancer Res..

[cit141] Nicholas C., Yang J., Peters S. B., Bill M. A., Baiocchi R. A., Yan F., Sïf S., Tae S., Gaudio E., Wu X., Grever M. R., Young G. S., Lesinski G. B. (2013). PLoS One.

[cit142] Chen M., Yi B., Sun J. (2014). J. Biol. Chem..

[cit143] Harris D. P., Bandyopadhyay S., Maxwell T. J., Willard B., DiCorleto P. E. (2014). J. Biol. Chem..

[cit144] Srour N., Khan S., Richard S. (2022). J. Inflammation Res..

[cit145] Litzler L. C., Zahn A., Meli A. P., Hébert S., Patenaude A.-M., Methot S. P., Sprumont A., Bois T., Kitamura D., Costantino S., King I. L., Kleinman C. L., Richard S., Di Noia J. M. (2019). Nat. Commun..

[cit146] Huang R., Guo J., Zhou Q., Huang H., Lin S., Lin Y., Petersen F., Yu X., Ma A. (2025). Sci. Rep..

[cit147] Hu M., Chen X. (2024). RSC Adv..

[cit148] Mavrakis K. J., McDonald E. R., Schlabach M. R., Billy E., Hoffman G. R., deWeck A., Ruddy D. A., Venkatesan K., Yu J., McAllister G., Stump M., deBeaumont R., Ho S., Yue Y., Liu Y., Yan-Neale Y., Yang G., Lin F., Yin H., Gao H., Kipp D. R., Zhao S., McNamara J. T., Sprague E. R., Zheng B., Lin Y., Cho Y. S., Gu J., Crawford K., Ciccone D., Vitari A. C., Lai A., Capka V., Hurov K., Porter J. A., Tallarico J., Mickanin C., Lees E., Pagliarini R., Keen N., Schmelzle T., Hofmann F., Stegmeier F., Sellers W. R. (2016). Science.

[cit149] Bonday Z. Q., Cortez G. S., Grogan M. J., Antonysamy S., Weichert K., Bocchinfuso W. P., Li F., Kennedy S., Li B., Mader M. M., Arrowsmith C. H., Brown P. J., Eram M. S., Szewczyk M. M., Barsyte-Lovejoy D., Vedadi M., Guccione E., Campbell R. M. (2018). ACS Med. Chem. Lett..

[cit150] Xia Q., Zhong R., Zheng J., Zhou X., Zhao X., Wang S., Wang B., Wu Q., Xie C., Kong B., Zhang Q., Huang T. (2025). Cell Rep..

[cit151] Lin H., Wang M., Zhang Y. W., Tong S., Leal R. A., Shetty R., Vaddi K., Luengo J. I. (2019). ACS Med. Chem. Lett..

[cit152] Jensen-Pergakes K., Tatlock J., Maegley K. A., McAlpine I. J., McTigue M., Xie T., Dillon C. P., Wang Y., Yamazaki S., Spiegel N., Shi M., Nemeth A., Miller N., Hendrickson E., Lam H., Sherrill J., Chung C. Y., McMillan E. A., Bryant S. K., Palde P., Braganza J., Brooun A., Deng Y. L., Goshtasbi V., Kephart S. E., Kumpf R. A., Liu W., Patman R. L., Rui E., Scales S., Tran-Dube M., Wang F., Wythes M., Paul T. A. (2022). Mol. Cancer Ther..

[cit153] Rodon J., Rodriguez E., Maitland M. L., Tsai F. Y., Socinski M. A., Berlin J. D., Thomas J. S., Al Baghdadi T., Wang I. M., Guo C., Golmakani M., Clark L. N., Gazdoiu M., Li M., Tolcher A. W. (2024). ESMO Open.

[cit154] Bewersdorf J. P., Mi X., Lu B., Kuykendall A., Sallman D., Patel M., Stevens D., Philipovskiy A., Sutamtewagul G., Masarova L., Keiffer G., Verma A., Bhagwat N., Wang M., Moore A., Rager J., Heiser D., Ro S., Hong W.-J., Abdel-Wahab O., Stein E. M. (2025). Leukemia.

[cit155] Zhang Y., Lin H., Wang M., Angelis D., Hawkins M., Rominger D., Emm T., Luengo J., Ruggeri B., Scherle P., Vaddi K. (2020). Cancer Res..

[cit156] Monga V., Johanns T. M., Stupp R., Chandra S., Falchook G. S., Giglio P., Philipovskiy A., Alnahhas I., Babbar N., Sun W., McKean M. (2023). J. Clin. Oncol..

[cit157] Hing Z. A., Walker J. S., Whipp E. C., Brinton L., Cannon M., Zhang P., Sher S., Cempre C. B., Brown F., Smith P. L., Agostinelli C., Pileri S. A., Skinner J. N., Williams K., Phillips H., Shaffer J., Beaver L. P., Pan A., Shin K., Gregory C. T., Ozer G. H., Yilmaz S. A., Harrington B. K., Lehman A. M., Yu L., Coppola V., Yan P., Scherle P., Wang M., Pitis P., Xu C., Vaddi K., Chen-Kiang S., Woyach J., Blachly J. S., Alinari L., Yang Y., Byrd J. C., Baiocchi R. A., Blaser B. W., Lapalombella R. (2023). Nat. Commun..

[cit158] Kawamura S., Palte R. L., Kim H.-Y., Saurí J., Sondey C., Mansueto M. S., Altman M. D., Machacek M. R. (2022). Bioorg. Med. Chem..

[cit159] Mao R., Shao J., Zhu K., Zhang Y., Ding H., Zhang C., Shi Z., Jiang H., Sun D., Duan W., Luo C. (2017). J. Med. Chem..

[cit160] Rong D., Zhou K., Fang W., Yang H., Zhang Y., Shi Q., Huang Y., Li J., Dong H., Li L., Ding J., Huang X., Wang Y. (2022). J. Med. Chem..

[cit161] Chan-Penebre E., Kuplast K. G., Majer C. R., Boriack-Sjodin P. A., Wigle T. J., Johnston L. D., Rioux N., Munchhof M. J., Jin L., Jacques S. L., West K. A., Lingaraj T., Stickland K., Ribich S. A., Raimondi A., Scott M. P., Waters N. J., Pollock R. M., Smith J. J., Barbash O., Pappalardi M., Ho T. F., Nurse K., Oza K. P., Gallagher K. T., Kruger R., Moyer M. P., Copeland R. A., Chesworth R., Duncan K. W. (2015). Nat. Chem. Biol..

[cit162] Duncan K. W., Rioux N., Boriack-Sjodin P. A., Munchhof M. J., Reiter L. A., Majer C. R., Jin L., Johnston L. D., Chan-Penebre E., Kuplast K. G., Porter Scott M., Pollock R. M., Waters N. J., Smith J. J., Moyer M. P., Copeland R. A., Chesworth R. (2016). ACS Med. Chem. Lett..

[cit163] Gerhart S. V., Kellner W. A., Thompson C., Pappalardi M. B., Zhang X.-P., Montes de Oca R., Penebre E., Duncan K., Boriack-Sjodin A., Le B., Majer C., McCabe M. T., Carpenter C., Johnson N., Kruger R. G., Barbash O. (2018). Sci. Rep..

[cit164] Gounder M. M., Martin-Romano P., Italiano A., Siu L. L., Cassier P. A., Falchook G. S., Lossos I. S., Rasco D. W., Hilton J. F., McKean M. A., Opdam F. L., Strauss J., De Jonge M., Vermaat J. S. P., Crossman T., Zajac M., Tarkar A., Gonzalez Carreras F., Kremer B. E., Barbash O., Segal S., Parasrampuria R., Postel-Vinay S. (2025). Ann. Oncol..

[cit165] Watts J., Minden M. D., Bachiashvili K., Brunner A. M., Abedin S., Crossman T., Zajac M., Moroz V., Egger J. L., Tarkar A., Kremer B. E., Barbash O., Borthakur G. (2024). Ther. Adv. Hematol..

[cit166] Qaddoura S. F., Liu E., Walker B. A., Tran N. T. (2025). Mol. Ther.: Oncol..

[cit167] Prabhu L., Wei H., Chen L., Demir Ö., Sandusky G., Sun E., Wang J., Mo J., Zeng L., Fishel M., Safa A., Amaro R., Korc M., Zhang Z. Y., Lu T. (2017). Oncotarget.

[cit168] Tao H., Yan X., Zhu K., Zhang H. (2019). Chem. Pharm. Bull..

[cit169] Brehmer D., Beke L., Wu T., Millar H. J., Moy C., Sun W., Mannens G., Pande V., Boeckx A., van Heerde E., Nys T., Gustin E. M., Verbist B., Zhou L., Fan Y., Bhargava V., Safabakhsh P., Vinken P., Verhulst T., Gilbert A., Rai S., Graubert T. A., Pastore F., Fiore D., Gu J., Johnson A., Philippar U., Morschhäuser B., Walker D., De Lange D., Keersmaekers V., Viellevoye M., Diels G., Schepens W., Thuring J. W., Meerpoel L., Packman K., Lorenzi M. V., Laquerre S. (2021). Mol. Cancer Ther..

[cit170] Vieito M., Moreno V., Spreafico A., Brana I., Wang J. S., Preis M., Hernández T., Genta S., Hansen A. R., Doger B., Galvao V., Lenox L., Brown R. J., Kalota A., Mehta J., Pastore F., Patel B., Mistry P., Gu J., Lauring J., Patel M. R. (2023). Clin. Cancer Res..

[cit171] Hulpia F., Schepens W., Lepri S., Nicolaï J., Jiang Z., Boj S. F., Bush T. L., Carvalho M.-a., Chen F., Chu G., Clancy K. W., Etwebi Z., Everaerts M., Fan Y., Fernandez Candelaria F. O., Francis A., Hixon M. S., Jardi F., Jin S., Larin E. M., Last S., Leenaerts J. E., Li S., Liddane A. G., Lutter F. H., Lv D., Mattson B., Milligan C. M., Patrick A. N., Patwardhan G. A., Perez-Benito L., Pieters S., Renders E., Retzbach E., Smith-Monroy C., Silva J., Silva M., Sterckx H., ten Hag G., Thäte C., Van Brandt S., Verissimo C. S., Verniest G., Vesely E., Vetrano I., Vinken P., Wang Y., Wong V., Yao X., Yang J., Zijlmans R., Bachman K. E., Pocalyko D., Jimenez J.-M., Gaffney D., Thuring J. W. (2025). J. Med. Chem..

[cit172] Kryukov G. V., Wilson F. H., Ruth J. R., Paulk J., Tsherniak A., Marlow S. E., Vazquez F., Weir B. A., Fitzgerald M. E., Tanaka M., Bielski C. M., Scott J. M., Dennis C., Cowley G. S., Boehm J. S., Root D. E., Golub T. R., Clish C. B., Bradner J. E., Hahn W. C., Garraway L. A. (2016). Science.

[cit173] Zhang M., Ding X., Cao Z., Yang Y., Ding X., Cai X., Zhang M., Aliper A., Ren F., Lu H., Zhavoronkov A. (2025). J. Med. Chem..

[cit174] Cottrell K. M., Briggs K. J., Whittington D. A., Jahic H., Ali J. A., Davis C. B., Gong S., Gotur D., Gu L., McCarren P., Tonini M. R., Tsai A., Wilker E. W., Yuan H., Zhang M., Zhang W., Huang A., Maxwell J. P. (2024). J. Med. Chem..

[cit175] Briggs K. J., Cottrell K. M., Tonini M. R., Tsai A., Zhang M., Whittington D. A., Zhang W., Lombardo S. A., Yoda S., Wilker E. W., Meier S. R., Yu Y., Teng T., Huang A., Maxwell J. P. (2025). Transl. Oncol..

[cit176] Cottrell K. M., Briggs K. J., Tsai A., Tonini M. R., Whittington D. A., Gong S., Liang C., McCarren P., Zhang M., Zhang W., Huang A., Maxwell J. P. (2025). J. Med. Chem..

[cit177] Briggs K. J., Tsai A., Zhang M., Zhang W., Huang A., Andersen J. N., Cottrell K. M. (2025). Cancer Res..

[cit178] Cottrell K. M., Briggs K. J., Tsai A., Liang C., McCarren P., Whittington D. A., Zhang M., Zhang W., Huang A., Andersen J., Maxwell J. P. (2026). J. Med. Chem..

[cit179] Smith C. R., Aranda R., Bobinski T. P., Briere D. M., Burns A. C., Christensen J. G., Clarine J., Engstrom L. D., Gunn R. J., Ivetac A., Jean-Baptiste R., Ketcham J. M., Kobayashi M., Kuehler J., Kulyk S., Lawson J. D., Moya K., Olson P., Rahbaek L., Thomas N. C., Wang X., Waters L. M., Marx M. A. (2022). J. Med. Chem..

[cit180] Engstrom L. D., Aranda R., Waters L., Moya K., Bowcut V., Vegar L., Trinh D., Hebbert A., Smith C. R., Kulyk S., Lawson J. D., He L., Hover L. D., Fernandez-Banet J., Hallin J., Vanderpool D., Briere D. M., Blaj A., Marx M. A., Rodon J., Offin M., Arbour K. C., Johnson M. L., Kwiatkowski D. J., Jänne P. A., Haddox C. L., Papadopoulos K. P., Henry J. T., Leventakos K., Christensen J. G., Shazer R., Olson P. (2023). Cancer Discovery.

[cit181] Smith J. M., Barlaam B., Beattie D., Bradshaw L., Chan H. M., Chiarparin E., Collingwood O., Cooke S. L., Cronin A., Cumming I., Dean E., Debreczeni J. É., del Barco Barrantes I., Diene C., Gianni D., Guerot C., Guo X., Guven S., Hayhow T. G., Hong T., Kemmitt P. D., Lamont G. M., Lamont S., Lynch J. T., McWilliams L., Moore S., Raubo P., Robb G. R., Robinson J., Scott J. S., Srinivasan B., Steward O., Stubbs C. J., Syson K., Tan L., Turner O., Underwood E., Urosevic J., Vazquez-Chantada M., Whittaker A. L., Wilson D. M., Winter-Holt J. J. (2024). J. Med. Chem..

[cit182] Smith J. M., Barlaam B., Beattie D., Bradshaw L., Chan H. M., Cooke S. L., Cronin A., Cumming I., Dean E., Debreczeni J. É., Barco Barrantes I. d., Ferguson D., Gianni D., Grondine M., Lynch J. T., McWilliams L., Moore S., Raubo P., Qu Y., Robb G. R., Tan L., Urosevic J., Vazquez-Chantada M., Wang P. (2026). J. Med. Chem..

[cit183] Belmontes B., Slemmons K. K., Su C., Liu S., Policheni A. N., Moriguchi J., Tan H., Xie F., Aiello D. A., Yang Y., Lazaro R., Aeffner F., Rees M. G., Ronan M. M., Roth J. A., Vestergaard M., Cowland S., Andersson J., Sarvary I., Chen Q., Sharma P., Lopez P., Tamayo N., Pettus L. H., Ghimire-Rijal S., Mukund S., Allen J. R., DeVoss J., Coxon A., Rodon J., Ghiringhelli F., Penel N., Prenen H., Glad S., Chuang C. H., Keyvanjah K., Townsley D. M., Butler J. R., Bourbeau M. P., Caenepeel S., Hughes P. E. (2025). Cancer Discovery.

[cit184] Sarvary I., Vestergaard M., Moretti L., Andersson J., Peiró Cadahía J., Cowland S., Flagstad T., Franch T., Gouliaev A., Husemoen G., Jacso T., Kronborg T., Kuropatnicka A., Nadali A., Madsen M., Nielsen S. r., Pii D., Ryborg S. r., Soede C., Allen J. R., Bourbeau M., Li K., Liu Q., Lo M.-C., Madoux F., Mardirossian N., Moriguchi J., Ngo R., Peng C.-C., Pettus L., Tamayo N., Wang P., Kapoor R., Belmontes B., Caenepeel S., Hughes P., Liu S., Slemmons K. K., Yang Y., Xie F., Ghimire-Rijal S., Mukund S., Glad S. (2025). J. Med. Chem..

[cit185] Cheng X., Hu Z., Mao H., Li Z., Wang Z., Tang Y., Huang S., Huang Y., Yin C., Xing H., Chen S., Jiang Y., Hu T., Zuo J., Yan W., Gu H., Wei P., Xu Y., Zhu Q., Zou Y. (2026). J. Med. Chem..

[cit186] Cheng X., Hu Z., Mao H., Li Z., Wang Z., Tang Y., Huang S., Huang Y., Yin C., Xing H., Chen S., Jiang Y., Hu T., Zuo J., Yan W., Gu H., Wei P., Xu Y., Zhu Q., Zou Y. (2026). J. Med. Chem..

[cit187] Yang Y., Chen Z., Wang Y., Huang X., Hu C., Yang H., Li Y. (2025). ACS Med. Chem. Lett..

[cit188] Ma N., Chen Y., Liang Y., Chen X., Song F., Su Z., Wang Y., Zhou J., Niu B., Hao Y., Li X., Huang W., Zheng M., Zhang S., Teng D. (2026). Bioorg. Chem..

[cit189] Palte R. L., Schneider S. E., Altman M. D., Hayes R. P., Kawamura S., Lacey B. M., Mansueto M. S., Reutershan M., Siliphaivanh P., Sondey C., Xu H., Xu Z., Ye Y., Machacek M. R. (2020). ACS Med. Chem. Lett..

[cit190] McKinney D. C., McMillan B. J., Ranaghan M. J., Moroco J. A., Brousseau M., Mullin-Bernstein Z., O'Keefe M., McCarren P., Mesleh M. F., Mulvaney K. M., Robinson F., Singh R., Bajrami B., Wagner F. F., Hilgraf R., Drysdale M. J., Campbell A. J., Skepner A., Timm D. E., Porter D., Kaushik V. K., Sellers W. R., Ianari A. (2021). J. Med. Chem..

[cit191] Hadjikyriacou A., Yang Y., Espejo A., Bedford M. T., Clarke S. G. (2015). J. Biol. Chem..

[cit192] Shen L., Ma X., Wang Y., Wang Z., Zhang Y., Pham H. Q. H., Tao X., Cui Y., Wei J., Lin D., Abeywanada T., Hardikar S., Halabelian L., Smith N., Chen T., Barsyte-Lovejoy D., Qiu S., Xing Y., Yang Y. (2024). Nat. Commun..

[cit193] Kröll-Hermi A., Stoetzel C., Etard C., Halabelian L., Schaefer E., Scheidecker S., Kahrizi K., Payman J., Geoffroy V., Prasad M., Obringer C., Ruch L., Girard A., Zeng H., Li F., Plassard D., Keime C., Mattioli F., Feger C., Piton A., Fujita A., Matsumoto N., Castro M. A. A., Ae K. C., Ruaud L., Levy J., Dozières B., Tabet A. C., Wentzensen I. M., Santiago-Sim T., Yusupov R., Tveten K., Smeland M. F., Alkhunaizi E., Cowing G., Li C., Wortmann S. B., Feichtinger R. G., Mayr J. A., Gonorazky H., Jing G., Wang X., Wang J., Bierhals T., Grinstein L., Herget T., Ruiz A., Gabau E., Kampmeier A., Kassel O., Kuechler A., Platzer K., Jamra R. A., Woerner A., Idleburg M., Kircher S. G., Laccone F., Golob B., Peterlin B., Čuturilo G., Tasic V., Kolvenbach C. M., Hildebrandt F., Ramos L. L. P., Kok F., Buck C. B., van de Laar I., de Man S. A., Taşdelen E., Sezer A., Büke A., Yavuz Z., Çomoğlu S. S., Costin C., Tran Mau Them F., Lacaze E., Courtin T., Héron D., Keren B., Whalen S., Roume J., Yang Y., Hoffer M. J. V., van Haeringen A., Najmabadi H., Arrowsmith C. H., Strähle U., Dollfus H., Muller J. (2025). Am. J. Hum. Genet..

[cit194] Dong H., He X., Zhang L., Chen W., Lin Y.-C., Liu S.-B., Wang H., Nguyen L. X. T., Li M., Zhu Y., Zhao D., Ghoda L., Serody J., Vincent B., Luznik L., Gojo I., Zeidner J., Su R., Chen J., Sharma R., Pirrotte P., Wu X., Hu W., Han W., Shen B., Kuo Y.-H., Jin J., Salhotra A., Wang J., Marcucci G., Luo Y. L., Li L. (2024). Nat. Cancer.

[cit195] Jiang H., Zhou Z., Jin S., Xu K., Zhang H., Xu J., Sun Q., Wang J., Xu J. (2018). Cancer Sci..

[cit196] Tholander F., Sjöberg B.-M. (2012). Proc. Natl. Acad. Sci. U. S. A..

[cit197] https://www.thesgc.org/chemical-probes/mrk-990

[cit198] Feoli A., Iannelli G., Cipriano A., Milite C., Shen L., Wang Z., Hadjikyriacou A., Lowe T. L., Safaeipour C., Viviano M., Sarno G., Morretta E., Monti M. C., Yang Y., Clarke S. G., Cosconati S., Castellano S., Sbardella G. (2023). J. Med. Chem..

[cit199] Jain K., Clarke S. G. (2019). Arch. Biochem. Biophys..

[cit200] Feng Y., Maity R., Whitelegge J. P., Hadjikyriacou A., Li Z., Zurita-Lopez C., Al-Hadid Q., Clark A. T., Bedford M. T., Masson J. Y., Clarke S. G. (2013). J. Biol. Chem..

[cit201] Haghandish N., Baldwin R. M., Morettin A., Dawit H. T., Adhikary H., Masson J. Y., Mazroui R., Trinkle-Mulcahy L., Côté J. (2019). Mol. Biol. Cell.

[cit202] Gao W.-w., Xiao R.-q., Peng B.-l., Xu H.-t., Shen H.-f., Huang M.-f., Shi T.-t., Yi J., Zhang W.-j., Wu X.-n., Gao X., Lin X.-z., Dorrestein P. C., Rosenfeld M. G., Liu W. (2015). Proc. Natl. Acad. Sci. U. S. A..

[cit203] Bikkavilli R. K., Avasarala S., Vanscoyk M., Sechler M., Kelley N., Malbon C. C., Winn R. A. (2012). Sci. Rep..

[cit204] Karkhanis V., Wang L., Tae S., Hu Y. J., Imbalzano A. N., Sif S. (2012). J. Biol. Chem..

[cit205] Zhu J., Li X., Cai X., Zha H., Zhou Z., Sun X., Rong F., Tang J., Zhu C., Liu X., Fan S., Wang J., Liao Q., Ouyang G., Xiao W. (2021). Mol. Cell.

[cit206] Ahn B. Y., Jeong M. H., Pyun J. H., Jeong H. J., Vuong T. A., Bae J. H., An S., Kim S. W., Kim Y. K., Ryu D., Kim H. J., Cho H., Bae G. U., Kang J. S. (2022). Cell. Mol. Life Sci..

[cit207] Kernohan K. D., McBride A., Xi Y., Martin N., Schwartzentruber J., Dyment D. A., Majewski J., Blaser S., Boycott K. M., Chitayat D. (2017). Clin. Genet..

[cit208] Baldwin R. M., Haghandish N., Daneshmand M., Amin S., Paris G., Falls T. J., Bell J. C., Islam S., Côté J. (2015). Oncotarget.

[cit209] Geng P., Zhang Y., Liu X., Zhang N., Liu Y., Liu X., Lin C., Yan X., Li Z., Wang G., Li Y., Tan J., Liu D.-X., Huang B., Lu J. (2017). FASEB J..

[cit210] Liu F., Wan L., Zou H., Pan Z., Zhou W., Lu X. (2020). Int. J. Biochem. Cell Biol..

[cit211] Cheng D., He Z., Zheng L., Xie D., Dong S., Zhang P. (2018). OncoTargets Ther..

[cit212] Srour N., Villarreal O. D., Hardikar S., Yu Z., Preston S., Miller W. H., Szewczyk M. M., Barsyte-Lovejoy D., Xu H., Chen T., del Rincón S. V., Richard S. (2022). Cell Rep..

[cit213] Liu C., Zou W., Nie D., Li S., Duan C., Zhou M., Lai P., Yang S., Ji S., Li Y., Mei M., Bao S., Jin Y., Pan J. (2022). Cell Metab..

[cit214] Li J., Sun C., Zhang Y., Ding J., Yao P., Shen H., Shi Z., Wang W., Zhu Y., Kuang W., Tavus A., Wang L., Yuan K., Wang X., Yang P. (2025). J. Med. Chem..

[cit215] Burgos E. S., Walters R. O., Huffman D. M., Shechter D. (2017). Chem. Sci..

[cit216] Smil D., Eram M. S., Li F., Kennedy S., Szewczyk M. M., Brown P. J., Barsyte-Lovejoy D., Arrowsmith C. H., Vedadi M., Schapira M. (2015). ACS Med. Chem. Lett..

[cit217] Szewczyk M. M., Ishikawa Y., Organ S., Sakai N., Li F., Halabelian L., Ackloo S., Couzens A. L., Eram M., Dilworth D., Fukushi H., Harding R., dela Seña C. C., Sugo T., Hayashi K., McLeod D., Zepeda C., Aman A., Sánchez-Osuna M., Bonneil E., Takagi S., Al-Awar R., Tyers M., Richard S., Takizawa M., Gingras A.-C., Arrowsmith C. H., Vedadi M., Brown P. J., Nara H., Barsyte-Lovejoy D. (2020). Nat. Commun..

[cit218] Zhong G., Chang X., Xie W., Zhou X. (2024). Signal Transduction Targeted Ther..

[cit219] Békés M., Langley D. R., Crews C. M. (2022). Nat. Rev. Drug Discovery.

[cit220] Xie S., Zhu J., Li J., Zhan F., Yao H., Xu J., Xu S. (2023). J. Med. Chem..

[cit221] Martin P. L., Pérez-Areales F. J., Rao S. V., Walsh S. J., Carroll J. S., Spring D. R. (2024). ChemMedChem.

[cit222] Ma C., Sun H., Shen C., Li X., Shen Y. (2025). Eur. J. Med. Chem..

[cit223] Zou W., Li M., Wan S., Ma J., Lian L., Luo G., Zhou Y., Li J., Zhou B. (2024). Adv. Sci..

[cit224] Li S., Pan W., Tao C., Hu Z., Cheng B., Chen J., Peng X. (2025). J. Med. Chem..

[cit225] Li S., Pan W., Tao C., Hu Z., Cheng B., Chen J., Peng X. (2025). J. Med. Chem..

[cit226] Xie H., Bacabac M. S., Ma M., Kim E.-J., Wang Y., Wu W., Li L., Xu W., Tang W. (2023). J. Med. Chem..

[cit227] Ju Y., Song H., He Y., Lo Y., Fan Z., Lu J. (2025). J. Med. Chem..

[cit228] Yang H., Zhang Q., Zhou S., Hu Z., Tang Q., Li Z., Feng Q., Yu L. (2024). Bioorg. Chem..

[cit229] Shen Y., Gao G., Yu X., Kim H., Wang L., Xie L., Schwarz M., Chen X., Guccione E., Liu J., Bedford M. T., Jin J. (2020). J. Med. Chem..

[cit230] Guo Y., Li Y., Zhou Z., Hou L., Liu W., Ren W., Mi D., Sun J., Dai X., Wu Y., Cheng Z., Wu T., Luo Q., Tian C., Li F., Yu Z., Chen Y., Chen C. (2024). J. Exp. Clin. Cancer Res..

[cit231] Zhong Y., Kim H., Qian C., Xie L., Chen X., Xiong Y., Hu J., Chen M., Guccione E., Shen Y., Jin J. (2025). J. Med. Chem..

